# Boronic ester-templated pre-rotaxanes as versatile intermediates for rotaxane *endo*-functionalisation†

**DOI:** 10.1039/d4sc04879b

**Published:** 2024-10-31

**Authors:** Jingjing Yu, Marius Gaedke, Satyajit Das, Daniel L. Stares, Christoph A. Schalley, Fredrik Schaufelberger

**Affiliations:** a KTH Royal Institute of Technology, Department of Chemistry Teknikringen 30 10044 Stockholm Sweden fresch@kth.se; b Institut für Chemie und Biochemie, Freie Universität Berlin Arnimallee 20 14195 Berlin Germany

## Abstract

We report on the synthesis of [2]rotaxanes from vicinal diols through dynamic covalent boronic ester templates, as well as the use of the boronic ester for rotaxane post-functionalisation. A boronic acid pincer ligand with two alkene-appended arms was condensed with a linear diol-containing thread, and ring-closing metathesis established a pre-rotaxane architecture along with a non-entangled isomer. Advanced NMR spectroscopy and mass spectrometry unambiguously assigned the isomers and revealed that the pre-rotaxane was in equilibrium with its hydrolyzed free [2]rotaxane form. The boronic ester handle in the pre-rotaxane could be synthetically addressed in a multitude of ways to obtain different *endo*-functionalised [2]rotaxanes, including with direct oxidation reactions, protodeboronation, functional group interconversions and Pd-catalysed cross-couplings.

## Abstract 2

Dynamic covalent boronic ester bonds can pre-organise diol-containing threads and V-shaped boronic acid ligands towards mechanical interlocking. After interlocking, the pre-rotaxane could be modified to create many unique [2]rotaxanes architectures.

## Graphical abstract

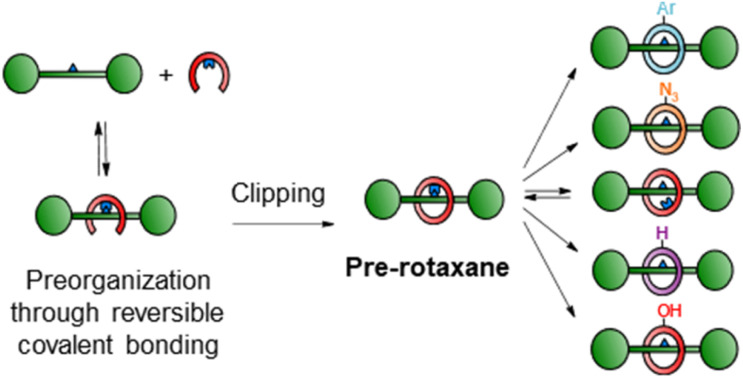

## Introduction

Mechanically interlocked molecules (MIMs) such as rotaxanes, catenanes and molecular knots have long been considered challenging synthetic targets.^1^ Most synthetic routes towards MIMs rely on non-covalent interactions to pre-organize the individual components before establishing the mechanical bond. However, this introduces extraneous functional groups such as ligands for metals (phenanthrolines, bipyridines), or electron-deficient aromatics (naphthalene diimides, viologens) which – depending on the aim for which they are synthesized – add potentially undesired functionality to the final MIMs.^1*a*^ As an alternative, covalent templates have also been used for constructing MIMs and typically display higher robustness and directionality than non-covalent interactions.^2^ This means mechanical bonds can be formed under more challenging conditions, and broader scope and synthetic versatility is often observed with covalent templates as compared to non-covalent. For example, covalent templates have enabled synthesis of MIMs with unusual sizes and shapes,^3^ higher-order sequence-specificity^4^ and previously inconceivable architectures such as all-peptide^5^ or all-benzene^6^ knots and links.

However, covalent templates still suffer the same critical limitations today as when first introduced decades ago,^7^ including harsh cleavage conditions, complicated synthesis and the requisite for exotic templating bonds.^2^ Furthermore, the template itself is often seen as a “necessary evil” in covalent template MIM synthesis. Introducing and eventually removing the templating covalent bond adds additional synthetic steps, lowers yields and typically offers only very limited options for diversification or new synthetic opportunities. This problem is largely arising due to the “catenand effect”,^8^*i.e.* the pronounced increase in steric protection of moieties encapsulated within the binding pocket of mechanical bonds.^9^

Dynamic covalent bonds have “Goldilocks character” that make them uniquely suited as covalent templates, with the bonds being robust enough for efficient covalent template synthesis (under one set of conditions), but labile enough to later liberate the free MIM (under another set of conditions).^10,11^ Boronic esters are classic examples of dynamic covalent bonds and form by exchange between diols and boronic acids.^12^ Reversible boronic acid complexation has been extensively used for biosensing,^13^ catalysis,^14^ self-healing materials,^15^ as well as in covalent organic frameworks,^16^ and there is already precedence for the use of boronic ester self-assembly to pre-organize components towards macrocyclization.^17^ However, while some boron-containing MIMs have been reported,^18^ the use of boron as a structural element or template for assembly of interlocked architectures is not well-established. During the preparation of this manuscript, an example of a rotaxane covalent template using tetrahedral boronate chemistry was published by Trolez and co-workers.^19^ While this highly interesting study highlights the power of boron to act as a gathering element for MIM synthesis, it is based on a capping approach rather than clipping and does not explore the synthetic versatility of the boron handle (since it is not using easily modifiable C–B bonds), nor demonstrate use of the widely accessible vicinal diol-element as a template.

**Fig. 1:**
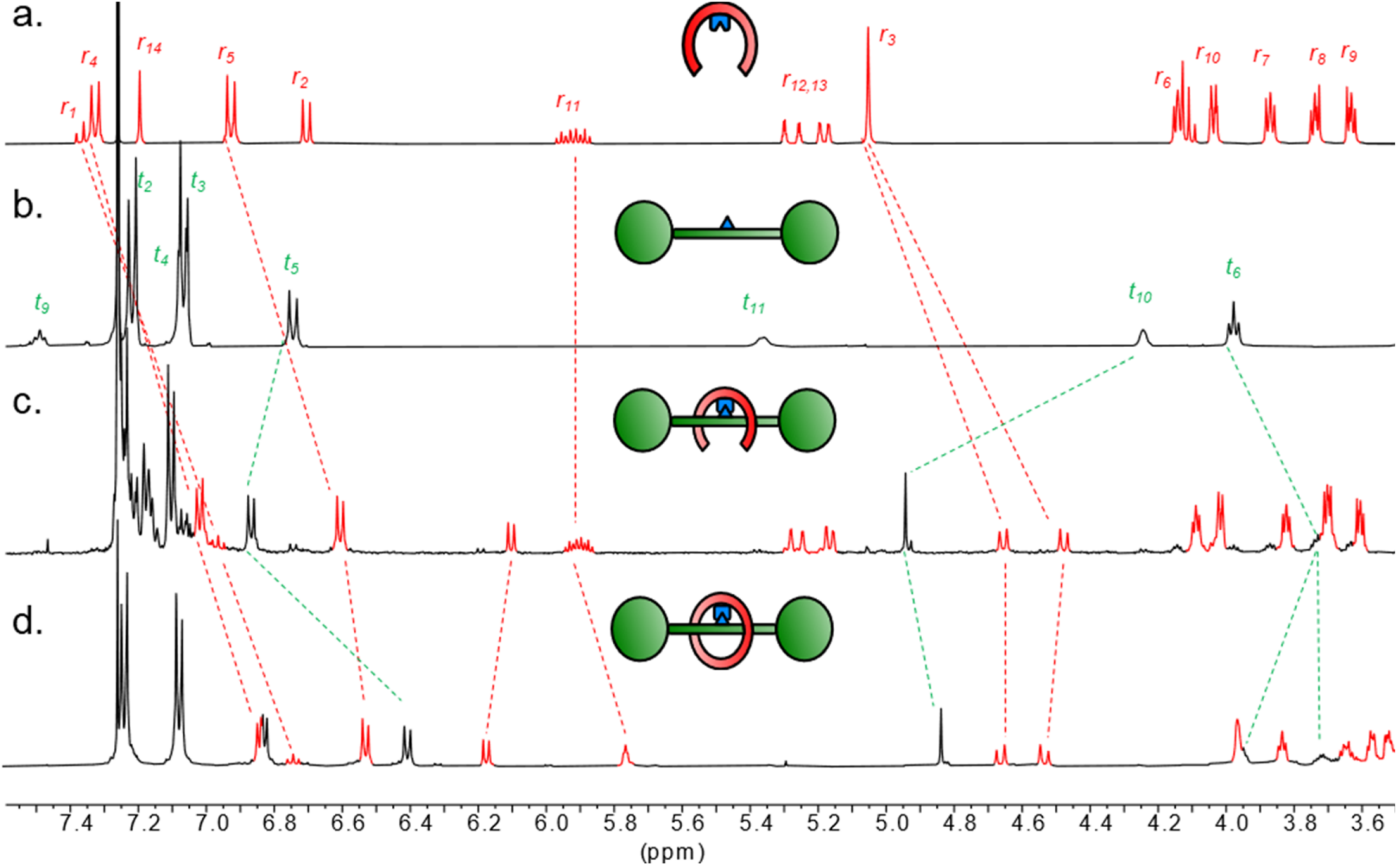
Partial ^1^H NMR spectra (500 MHz, CDCl_3_, 298 K) of (a) boronic acid pincer ligand 1; (b) diol thread 2; (c) boronic ester condensation product 3 (89% conversion); (d) pre-rotaxane 4.

In this report, we demonstrate that dynamic covalent boronic ester bonds circumvent the disadvantages of previous covalent templates and shows little to no catenand effect. We use dynamic covalent templating to pre-organize a diol-containing thread and a V-shaped boronic acid pincer receptor into a “clasp-type” conformation that delivers a [2]rotaxane upon ring closure and cleavage of the dynamic linkage (Fig. 1). Our design enables clipping of the receptor onto the thread. The dynamic covalent bond can then be reversibly broken through hydrolysis or derivatized through deboronylation, functional group interconversion or metal-catalysed cross-coupling chemistry to create a range of [2]rotaxanes from a single intermediate. All derivatisations also occur at the endo-position (*i.e.* on the inside of the macrocycle), which is selectivity not achievable through any other rotaxane post-functionalisation strategies, and of high interest for applications where tailoring the rotaxane cavity is of importance (*i.e.* catalysis, sensing). Hence, this study shows that dynamic boronic ester chemistry is a highly useful tool for [2]rotaxane synthesis and diversification.

## Results and discussion

**Scheme 1:**
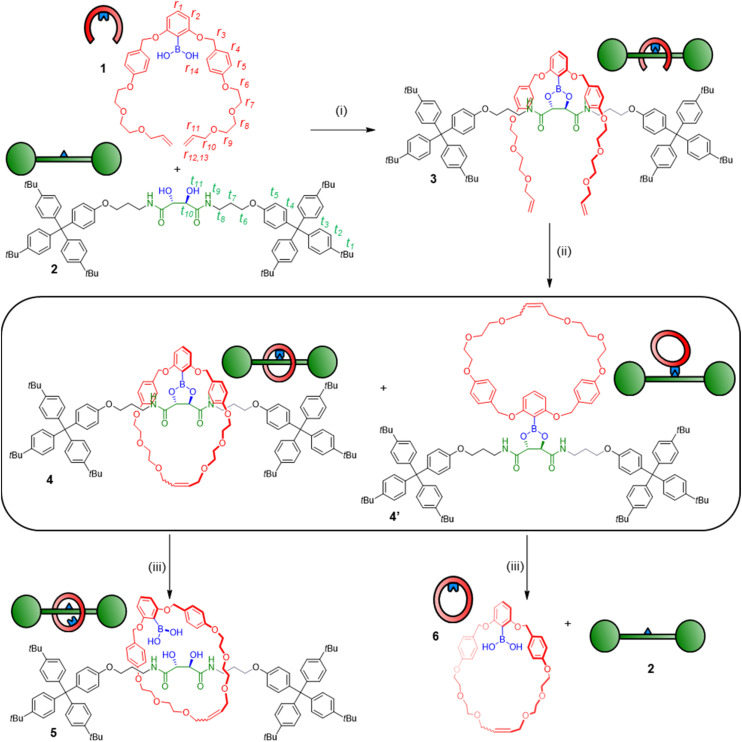
Synthesis of a [2]rotaxane through boronic acid/diol condensation. Reagents and conditions: (i) 1 (1 equiv.), 2 (1 equiv.), toluene, RT, 24 h; (ii) Hoveyda–Grubbs 2nd generation catalyst, CH_2_Cl_2_, RT, 24 h, 16% yield over two steps for 4, 33% yield over two steps for 4′; (iii) H_2_O, CDCl_3_, RT, 1–7 days.

The V-shaped pincer ligand 1 (Scheme 1) bears a boronic acid moiety at its cleft and was synthesized in six steps as outlined in the ESI (Section S3†).^20^ As thread, we synthesized model compound 2 in five steps starting from l-tartaric acid. To ensure a stable thread architecture and facilitate synthesis, we used amide bonds to connect the tartrate unit to the stoppers. The pincer ligand 1 and thread 2 were mixed in anhydrous toluene, leading to spontaneous self-assembly of the dynamic boronic ester 3 in 67–89% conversion, as determined by ^1^H NMR analysis (Fig. 1 and Table S1†).‡‡Condensation yield had a large variability under repeat conditions or even in glovebox settings. Attempts to increase condensation yield by switching solvents and concentrations failed, as did addition of a range of drying agents and use of a Dean–Stark apparatus. Instead of increased conversion to boronic ester, we observed boroxine trimerization of the ligand under such conditions, indicating that the desired cleft-receptor 3 is likely a metastable reaction product. Pronounced ^1^H NMR spectral shifts for key resonances indicated successful complexation. For example, proton t_10_ shifts strongly downfield (Δ*δ* = 0.7 ppm), while t_6_ shifts upfield (Δ*δ* = 0.2 ppm), indicating boronic ester formation and the arms of the pincer shielding the thread *via* the desired clasp-type conformation. Protons r_1_, r_2_, r_4_ and r_5_ on the pincer also shift noticeably, again indicating a more rigidified environment along with boronic ester formation. Aside from an upfield shift of 0.4 ppm, the splitting pattern of proton r_3_ also changes from singlet to a doublet of doublets, due to the diastereotopic protons now residing in a conformationally restricted chiral environment imposed by the tartrate chiral centers. ^11^B NMR spectroscopy gave a shift of 28.9 ppm for 3, which supports assignment of the B atom as a trigonal boronic ester (spectrum S29).^21^ Reversibility of the linkage was confirmed by hydrolysing the complex back to 1 and 2 in water-saturated CDCl_3_ (Fig. S1 and S2†).

The covalently linked clasp-type receptor 3 is pre-organized for rotaxane formation using ring-closing metathesis (RCM).^22^ The flexibility of the structure was deemed necessary for the arms to meet during ring closure as part of the RCM catalytic cycle, where an organometallic Ru complex is covalently attached to one chain terminus.^23^ Indeed, treatment of complex 3 with the Grubbs–Hoveyda 2nd generation catalyst in CH_2_Cl_2_ led to efficient ring closure, as evidenced by ^1^H NMR spectra of the crude mixture (Fig. S3†). The majority of the crude reaction mixture appeared to be composed of two ring-closed species in 2 : 1 ratio (the remainder being unidentified oligo- and polymeric species). Analysis using electrospray ionization high resolution mass spectrometry (ESI-HRMS) indicated both species had the same exact mass of *m*/*z* 1817.0221 (calculated for [C_116_H_139_N_2_O_14_ + Na]^+^: *m*/*z* 1817.0253) as expected for the desired pre-rotaxane product 4 (Fig. 3b, c and S7†).

Clearly, the two compounds have the same elemental composition and are thus the structural isomers 4 and 4′ (Scheme 1), stemming from ring closure around the thread (to generate the pre-rotaxane 4) and outside of the thread (to generate the non-interlocked isomer 4′). Separation of the two compounds was achieved by column chromatography, and we could hence isolate the suspected pre-rotaxane 4 as the minor product. The interlocked nature of 4 was clear already from its physical properties, as the compound was fully stable to chromatographic purification despite the hydrolytically sensitive boronic ester moiety. In contrast, attempts to isolate the major product 4′ in pure form were fruitless, as this non-interlocked isomer hydrolyzed readily during chromatographic purification attempts or after being dissolved in wet organic solvents.

For interlocked compound 4, ^1^H NMR analysis showed full consumption of the terminal alkene protons r_12_/r_13_ in 3 and the characteristic change in both shift and splitting pattern (m to t) of the internal alkene proton r_11_, corresponding to the ring-closed metathesis product (Fig. 1). *E*/*Z* ratio could be approximated as 4 : 1 through NMR analysis of the integrals of r_11_. Large upfield shifts for peaks corresponding to protons on the arms of the pincer receptor (r_3_, r_4_, r_5_) as well as the thread (t_5_, t_6_) indicated the ring closing event produced a tight conformation with close association to the thread, in line with the expected interlocked conformation.

From this NMR analysis, we could now assign compound 4 as the minor product in the reaction mixture. A higher quantity of the exo-macrocyclic conformer 4′ was, correspondingly, also observed. Through rapid silica flash column chromatography we could isolate a mixture of 4′ together with the hydrolysis products, thread 2 and boronic-acid macrocycle 6 (Scheme 1 and Fig. S5, S6†).^24^ After re-isolation with this protocol, thread 2 could also be used as starting material to generate more compound 4.

**Fig. 2:**
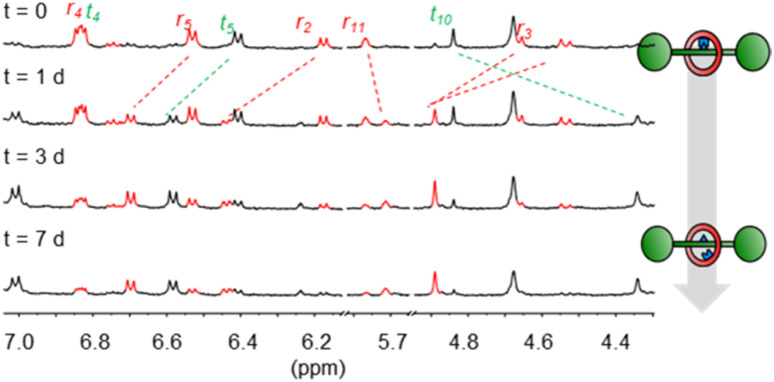
Partial ^1^H NMR spectra (500 MHz, CDCl_3_, 298 K) showing hydrolysis of 4 to generate 5 over time.

In contrast to some previously used covalent templates,^2^ the boronic ester functionality is labile and requires only mild conditions to dissociate. Indeed, pre-rotaxane 4 was found to slowly equilibrate to the free [2]rotaxane 5 when left in water-saturated CDCl_3_ under ambient conditions, indicating that the “catenand effect” is not preventing liberation of the free [2]rotaxane species. After several days, an equilibrium position of 70 : 30 between 5 and 4 was established (Fig. 2). Considering the effective molarity between diol and boronic acid in 5, the shift of the equilibrium position towards the hydrolysis product is somewhat unexpected and indicates that the boronic acid state is strongly favoured over the corresponding ester. In contrast, the non-interlocked nature of 4′ was obvious from its chemical instability. Upon being left in wet CDCl_3_ for 24 h, 4′ had dissociated to free 2 and macrocycle 6, with >95% conversion to these products being observed after 48 h (Scheme 1 and Fig. S5†). By condensing the free thread 2 and macrocycle 6 under conditions similar to those used for the formation of complex 3, we could also regenerate 4′*in situ* (Fig. S6†).

**Fig. 3:**
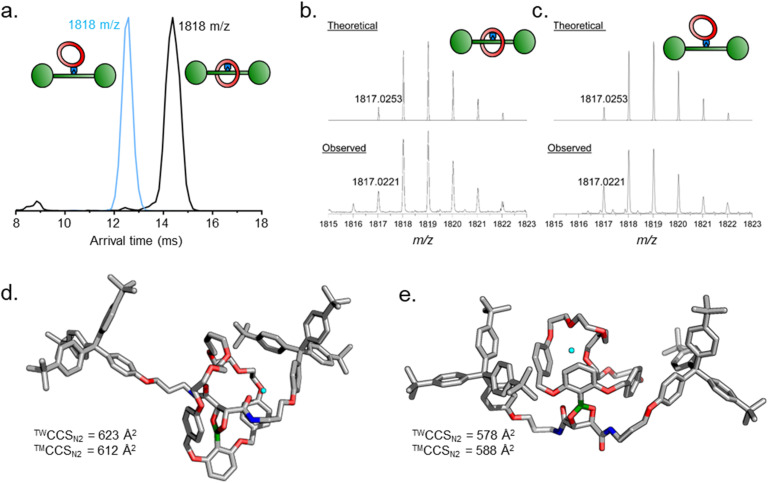
Tandem mass spectrometry investigations of 4 and 4′. (a) Superimposed TW-IMS arrival time distributions for 4 and 4′. (b) HRMS isotopic distribution for 4. (c) HRMS isotopic distribution for 4′. (d) and (e) HF-3c molecular models for (d) [4·Na]^+^ and (e) [4′·Na]^+^ used for calculation of theoretical collisional cross sections (™CCS_N2_) and comparison with experimental collisional cross section values (^TW^CCS_N2_). Na^+^ ion shown in cyan.

Tandem mass spectrometry is a useful tool to further corroborate the identities of 4 and 4′. The isolated samples were first subjected to traveling wave ion mobility mass spectrometry^25^ (TW-IMS), which revealed different arrival times for 4 (14.4 ms) and 4′ (12.5 ms) (Fig. 3a and Table S2†).§§Analysis of the crude reaction mixture also yielded a mixture of species with both arrival times, in line with expectations. Experimental collision cross section (^TW^CCS_N2_) values, determined by calibration with a polyalanine standard (full details in ESI†), were calculated to be 623 ± 3 and 578 ± 4 Å^2^, for 4 and 4′, respectively. Theoretical collisional cross section (™CCS_N2_) were obtained with the trajectory method using HF-3c-optimized structures of the Na^+^ adducts of 4 and 4′ (612 and 588 Å^2^, respectively), and were in good agreement with the experimental values (Fig. 3d and e).^26^ It may seem counter-intuitive that the non-intertwined structure 4′ is smaller in size compared to the interlocked structure 4. The ring in 4′ is however more flexible and can more easily adjust to optimize self-solvation with the axle arms, which is much stronger in the absence of solvent.^27^ Consequently, the axle arms wrap around the ring leading to a more compact structure. These measurements both validate our tentative previous compound assignments and demonstrate the power of TW-IMS to distinguish very closely related isomers such as 4 and 4′.

We further used collision-induced dissociation (CID) experiments to investigate the linkage between ring and thread in the RCM products by following the fragmentation of the hydrolysis products (Fig. S9 and S10†). Hydrolysis of 4′ was induced by dissolving the sample in a 4 : 1 MeCN/H_2_O mixture and incubating for 1 h before measuring. We detected the hydrolysis complex [4′·2H_2_O] (or [2·6]) in the MS and subsequently isolated this peak for fragmentation. This complex between free ring 6 and thread 2 dissociated readily under CID conditions, with essentially full dissociation already at a collision voltage of 20 V, indicating that this species is held together only through weaker interactions such as H-bonds rather than a mechanical bond. After incubating 4 under the same hydrolysis conditions, free [2]rotaxane 5 was observed (*m*/*z* 1854) and selected for fragmentation by CID. Much higher collision voltages (>70 V) were required to induce fragmentation of 5 which interestingly underwent a double condensation to re-form the pre-rotaxane 4 concomitant with two water losses first. Upon further increasing the collision voltage to 90 V, the dissociation of wheel and axle was also observed as a second competing channel. Likely, one of the benzyl ether groups in the wheel is cleaved in the first step of this dissociation reaction. Similarly high collision voltages were needed for the fragmentation of non-hydrolysed 4 and 4′ where no specific thread/ring fragments were observed, as expected for entirely covalently linked molecules (Fig. S11 and S12†). These measurements clearly show 5 to be a mechanically interlocked molecule, while also demonstrating the power of advanced mass spectrometry to solve complex problems in supramolecular chemistry.

The l-tartrate unit used as template is inherently chiral, and hence we also analyzed the rotaxane assembly by circular dichroism (CD) spectroscopy (Fig. S13†). In line with expectations, the achiral boronic acid macrocycle 6 yielded no CD response. The response from free thread 2 was also very small, probably due to the large distance of the stopper chromophore units from the chiral center (*i.e.* no chiral conformations were populated to a meaningful extent). In contrast, the pre-rotaxane 4 displayed pronounced chiral response and a Cotton effect with a maximum at 298 nm, showing transfer of chiral information from the l-tartrate template to the macrocycle component and again indicating a tight association between the MIM components.

**Scheme 2:**
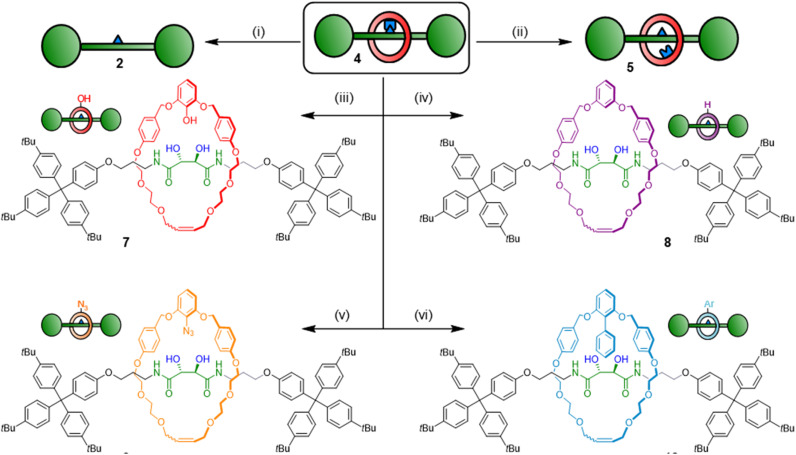
Derivatization of pre-rotaxane 4. Reagents and conditions: (i) H_2_O_2_, NaOH, THF/H_2_O 1 : 1, RT, 1 h, 87%. (ii) H_2_O, CDCl_3_, RT, 7 days (equilibrium yield 70%). (iii) H_2_O_2_, THF/H_2_O 1 : 1, RT, 1 h, 67%. (iv) Cu(OAc)_2_, MeOH/CH_2_Cl_2_ 2 : 1, 55 °C, 16 h, 82%. (v) NaN_3_, Cu(OAc)_2_, DMF, 55 °C, 16 h, 78%. (vi) Bromobenzene, Pd_2_(dba)_3_, RuPhos, NaO*t*Bu, DMF, 110 °C, 24 h, 35%.¶

Pre-rotaxane 4 is assembled through a boronic ester linkage. One advantage of boronic esters in organic chemistry is their versatility as synthetic handles, and naturally we envisioned that our boronic ester template could be used to create many different interlocked architectures *via* post-functionalisation.^28^ Furthermore, the boron-containing endo-position *vis-à-vis* the thread is sterically heavily shielded and difficult to post-functionalise under other conditions. We hence tried exposing 4 to different derivatization conditions (Scheme 2). As previously mentioned, free [2]rotaxane 5 is accessed through exposure to water-saturated CDCl_3_ over extended time periods. Addition of strong acids (H_2_SO_4_ and HCl) or bases (NaOH) led to ring cleavage *via* benzyl ether dissociation. Under optimized conditions, the free thread could be liberated in 87% yield upon treatment with NaOH/H_2_O_2_. More importantly, by using milder conditions to selectively address the boronic ester center we could obtain several different [2]rotaxane derivatives in good yields. Dissolving compound 4 in a 1 : 1 THF/H_2_O mixture with H_2_O_2_ for 1 h induced transformation to phenol rotaxane 7 in 67% isolated yield (spectra S41–S46, S9). Protodeboronation to create the proton-exchanged rotaxane 8 could also be induced by treatment of 4 with Cu(OAc)_2_ in a protic solvent mixture (MeOH/DCM), leading to a yield of 82%. Encouraged by these results, we attempted to exchange the boron centre for other functional groups. As a test reaction, we chose an azide functional group interconversion with NaN_3_ and Cu(OAc)_2_ in DMF, which satisfyingly generated the azide rotaxane in 78% yield after overnight reaction at 55 °C.^29^ Finally, we attempted a Pd-catalysed cross coupling using 4 as substrate, *i.e.* a [2]rotaxane liberation by Suzuki reaction. Conditions developed for highly sterically congested boronic esters turned out to be suitable for this chemistry,^30^ and we could indeed generate a rotaxane with a cross-coupled macrocycle using bromobenzene, Pd_2_(dba)_3_, RuPhos ligand and NaO*t*Bu, though further optimization of this protocol is still needed.¶¶The Suzuki protocol generates cross-coupled rotaxane 10 with protodeboronation product 8 as an inseparable mixture in 55 : 45 ratio. Separation difficulties likely arise from the very similar polarities of the two molecules. These post-assembly modification experiments demonstrate that we can selectively address the pre-rotaxane scaffold in many different ways: macrocycle cleavage to liberate the concealed thread, thermodynamic ring-thread equilibration or derivatization of the macrocycle to obtain kinetically trapped [2]rotaxanes 7–10 with different endo-substituents on the ring component. This usage of the MIM template as a functional handle to derivatise and obtain a wide range of interlocked architectures under both thermodynamic and kinetic control is unprecedented and indicates that boronic ester templates could find use in constructing diverse and complex MIMs that are difficult or impossible to obtain with other methods.

## Conclusions

In summary, we have demonstrated that dynamic covalent boronic ester bonds that form between boronic acids and vicinal diols can template rotaxane formation, and that the resulting pre-rotaxane is a versatile synthetic intermediate for creation of a range of *endo*-functionalised [2]rotaxanes bearing different functionality. The dynamic linkage brings the two components into proximity, and the V-shaped boronic acid ligand is pre-organized to obtain an interlocked product in the ring closing step. The isolated pre-rotaxane is chemically stabilized by the mechanical bond, but could still be easily derivatized in several ways through judicious choice of conditions. This post-functionalisation strategy allows exquisite fine-tuning of the interior of the macrocycle. Considering the importance of tailoring the size, shape and functionality of the cavity generated by the mechanical bond in for example mechanically interlocked sensors,^31^ ligands^32^ and catalysts,^33^ this methodology will likely be useful to researchers in these fields.

This constitutes a proof-of-concept for the use of the boronic ester motif as template and derivatisation handle for mechanical bond formation. The use of dynamic covalent bonds for this purpose is critical, as bond cleavage in this system is facile and efficient, circumventing the stabilizing “catenand effect” that has hindered template removal in previous examples of covalently templates MIMs.^2,9^

While the compounds in this work are largely artificial, it should also be pointed out that the core binding motif here is the natural product l-tartrate. Polyhydroxylated scaffolds and vicinal diols are ubiquitous in nature (RNA, carbohydrates, natural products *etc.*), meaning it might in the future be possible to use other diol-containing native biomolecules for MIM formation.^34^ Work along these lines is currently underway in our laboratories.

## Data availability

Experimental procedures, optimization data, NMR analysis and mass spectra can be found in the ESI.†

## Author contributions

J. Y. planned and performed the synthetic work. J. Y., M. G., S. D. and F. S. did measurements and analysis of the synthetic data. D. L. S. and C. A. S. performed the tandem mass spectrometry and computational investigations. F. S. conceived the project, directed the work and wrote the manuscript. The manuscript was edited and proof-read by all authors.

## Conflicts of interest

There are no conflicts to declare.

## Supplementary Material

SC-015-D4SC04879B-s001
