# Supplementary material for: Boronic ester-templated pre-rotaxanes as versatile intermediates for rotaxane endo-functionalisation
Source: Chem Sci. 2024 Oct 31;15(46):19443–51. doi: 10.1039/d4sc04879b (PMC11575644; doi:10.1039/d4sc04879b)
Supplement: SC-015-D4SC04879B-s001 [file SC-015-D4SC04879B-s001.pdf]

Supplementary Information (SI) for Chemical Science.
This journal is © The Royal Society of Chemistry 2024

Boronic Ester-Templated Pre-Rotaxanes as Versatile Intermediates for Rotaxane Endo-functionalisation

Jingjing Yu[1], Marius Gaedke[1], Satyajit Das[1], Daniel L. Stares[2], Christoph A. Schalley[2], Fredrik Schaufelberger[1]*

1. KTH Royal Institute of Technology, Department of Chemistry, Teknikringen 30, 10044 Stockholm, Sweden
2. Institut für Chemie und Biochemie, Freie Universität Berlin, Arnimallee 20, 14195 Berlin, Germany.

*E-mail: fresch@kth.se

-Supporting Information-

Table of Contents

S1. Abbreviations	S3
S2. General experimental	S4
S3. Reaction schemes	S6
S4. Experimental procedures	S9
S5. Supplementary data	S19
S6. Mass Spectra	S28
S7. NMR Spectra	S34
S8. Supplementary information references	S56

S1. ABBREVIATIONS

Abbreviations: COSY correlation spectroscopy; CID collision induced dissociation; DEPT distortionless enhancement by polarization transfer; DMF *N*,*N*-dimethylformamide; DIPEA *N,N*-diisopropylethylamine; DMSO dimethylsulfoxide; ESI electrospray ionization; HMBC heteronuclear multiple bond correlation; HRMS high resolution mass spectrometry; HSQC heteronuclear single quantum coherence spectroscopy; MeCN acetonitrile; NMR Nuclear magnetic resonance; RCM ring closing metathesis; RT room temperature; TFA trifluoroacetic acid; THF tetrahydrofuran; TLC thin layer chromatography; $^{TM}CCS_{N2}$ theoretical experimental collisional cross section calculated using the trajectory method; $^{TW}CCS_{N2}$ experimental collisional cross section; TW-IMS travelling wave ion mobility spectrometry

S2. GENERAL EXPERIMENTAL

Unless stated otherwise, reagents were obtained from commercial sources and used without purification. Reactions were carried out in anhydrous solvents and under an N_2 atmosphere. Anhydrous solvents were obtained by passing the solvent through an activated alumina column in a Glass Contour solvent dispensing system and stored over molecular sieves Anhydrous THF was collected from redistillation with freshly-cut sodium wire and benzophenone indicator. Compounds **S1**,[1] **S6**[2], **S7**[2], **S8**[3], **S9**[3] and **S10**[4] were synthesized as previously described. ^{1}H NMR spectra were recorded on a Bruker Avance DMX 500 MHz NMR spectrometer and a Bruker Ascend 400 spectrometer (400 MHz). Chemical shifts are reported in parts per million (ppm) from high to low frequency using the residual solvent peak as the internal reference ($CDCl_3$ = 7.26 ppm). All ^{1}H resonances are reported to the nearest 0.01 ppm. The multiplicity of ^{1}H signals are indicated as: s = singlet; d = doublet; t = triplet; q = quartet; m = multiplet; br = broad; app = apparent; or combinations thereof. Coupling constants (*J*) are quoted in Hz and reported to the nearest 0.1 Hz. Where appropriate, averages of the signals from peaks displaying multiplicity were used to calculate the value of the coupling constant. ^{13}C NMR spectra were recorded on the same spectrometers with the central resonance of the solvent peak as the internal reference ($CDCl_3$ = 77.16 ppm). All ^{13}C resonances are reported to the nearest 0.01 ppm. For new compounds, DEPT, COSY, HSQC and HMBC experiments were used to aid spectral assignment. Fully characterized compounds were chromatographically homogeneous except where indicated otherwise in experimental descriptions. Computational models were optimized using HF-3c implemented in the ORCA software version 5.0.4.[5-7]

Flash column chromatography was carried out using Silica 60 Å (particle size 40–63 µm, Merck, Sweden) as the stationary phase. TLC was performed on precoated silica gel plates (0.25 mm thick, 60 F_{254}, Merck, Germany) and visualized using both short and long wave ultraviolet light in combination with standard laboratory stains (basic potassium permanganate, acidic ammonium molybdate). High-resolution mass spectrometry was performed on an LC-MS-QTOF 6530C instrument (Agilent).

TW-IMS and CID measurements: Ion mobility mass spectrometry measurements were performed on a Synapt G2-S traveling wave ion-mobility mass spectrometer Q-TOF (Waters) equipped with a Z-spray electrospray ionization source. Ion mobility samples were prepared with a final concentration of 25 µM in MeCN and were injected with a flow rate of 10 µL/min. Hydrolysis was initiated by preparing a 25 µM sample in 4:1 MeCN/H_2O. A capillary voltage of 3.75 kV was used with the sample cone and source offset both set to 25 eV. The source and desolvation temperatures were both set to 40°C. N_2 was used as the collision gas. The drift

cell was operated with N_2 as the drift gas and was turned on 45 minutes prior to measuring to allow the pressures to settle. $^{TW}CCS_{N2}$ were determined with a polyalanine calibrant solution and performed with 5 different wave height and velocities according to a literature procedure.[8] Theoretical $^{TM}CCS_{N2}$ values were calculated using the trajectory method implemented in IMoS software (Larriba Lab).[9] Arrival time distributions were fitted with a modified gaussian equation in Origin pro 2020 (OriginLab corporation) to determine precise arrival times. CID was performed in transfer cell with N_2 as collision gas.

S3. REACTION SCHEMES

3.1. Synthesis of tartrate diol thread and boronic open ring

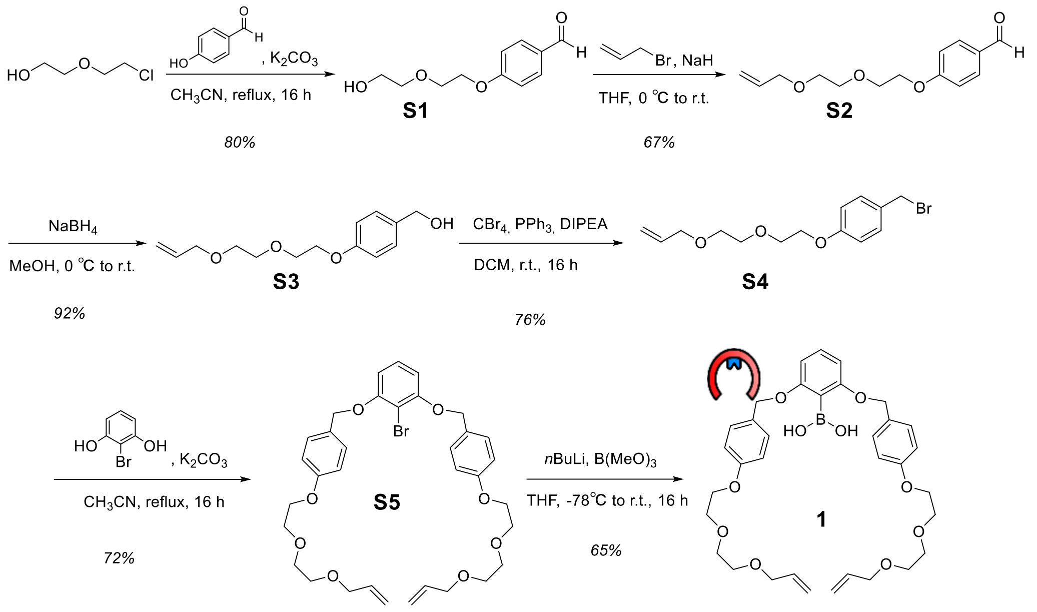

Scheme S1. Synthesis of boronic acid pincer ligand **1**.

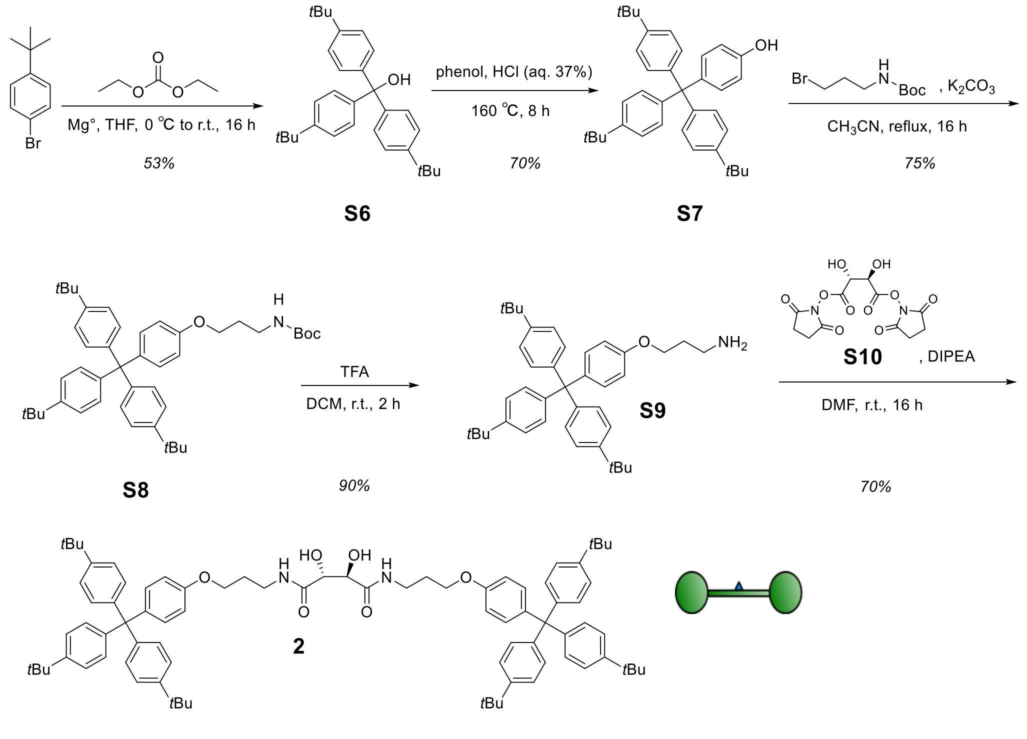

Scheme S2. Synthesis of tartrate diol thread **2**.

3.2. Synthesis of quasi[1]rotaxane and [2]rotaxane

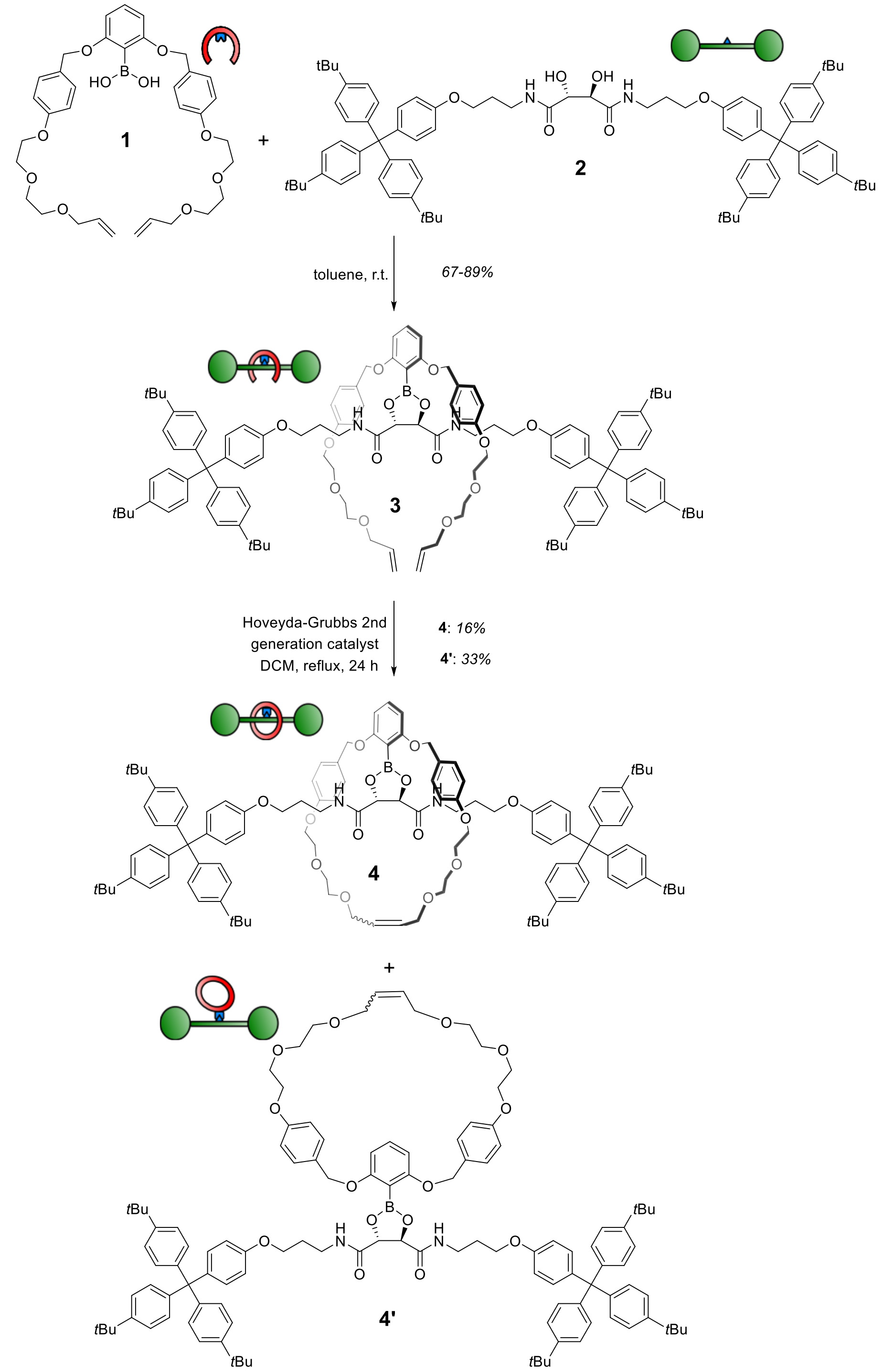

Scheme S3. Synthesis of quasi[1]rotaxane **4** and isomer **4'**.

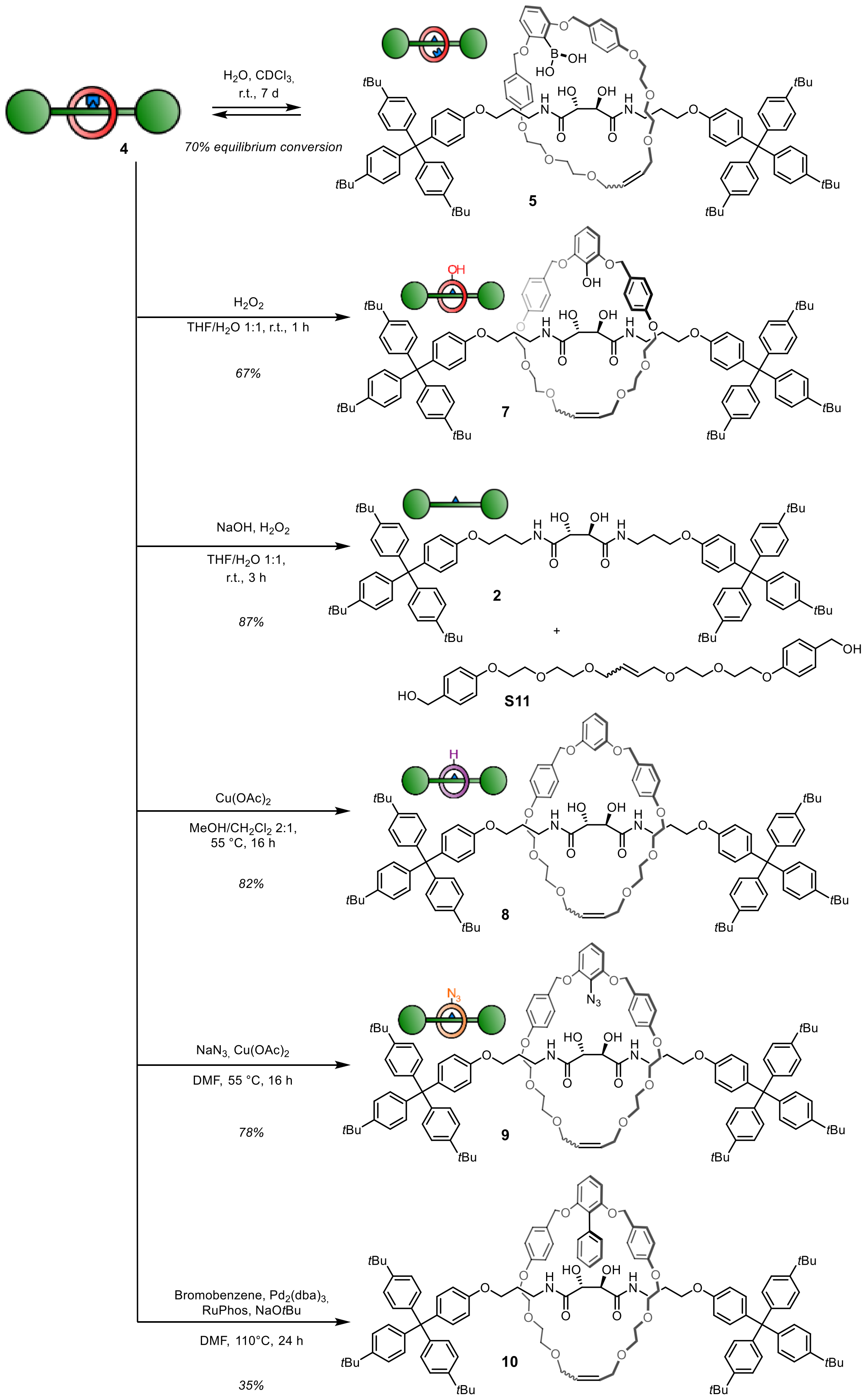

Scheme S4. Derivatization reactions of quasi[1]rotaxane **4**.

S4. EXPERIMENTAL PROCEDURES

S4.1 Synthetic procedures and characterization details

S2

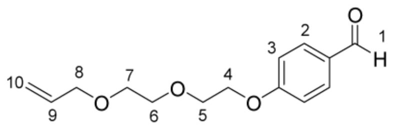

Sodium hydride (60% in mineral oil; 4.9 g, 122 mmol) was placed in a 200 mL, three-necked flask. The system was filled with dry nitrogen gas and cooled to 0 °C, after which THF (45 mL) was added. Compound **S1** (13 g, 50 mmol) was added dropwise to the suspension under stirring. After hydrogen gas evolution finished, allyl bromide (10.6 mL, 124 mmol) was added dropwise at 0 °C, and the mixture was stirred for 16 h at room temperature. Afterwards, the system was cooled to 0 °C and water (50 mL) was slowly added to quench the reaction. The mixture was transferred into a separating funnel and extracted repeatedly with ethyl acetate. The organic phase was washed with brine and dried over $MgSO_4$, filtered and concentrated. The residue was purified with column chromatography (silica gel; hexane/ethyl acetate, 2:1), to obtain compound **S2** (10 g, 67% yield) as a colorless oil. **^{1}H NMR** (500 MHz, $CDCl_3$): δ 9.88 (s, 1H, H_1), 7.82 (d, J = 8.5 Hz, 2H, H_2), 7.02 (d, J = 8.5 Hz, 2H, H_3), 5.84-5.98 (m, 1H, H_9), 5.27 (dd, J = 17.5 Hz, 2.0 Hz, 1H, $H_{trans10}$), 5.18 (dd, J = 10.5 Hz, 1.5 Hz, 1H, H_{cis10}), 4.16-4.27 (m, 2H, H_4), 4.03 (dt, J = 5.5 Hz, 1.5 Hz, 2H, H_8), 3.85-3.92 (m, 2H, H_5), 3.70-3.77 (m, 2H, H_6), 3.58-3.65 (m, 2H, H_7); **^{13}C NMR** (126 MHz, $CDCl_3$): δ 190.92, 163.99, 134.78, 132.07, 130.20, 117.35, 115.02, 72.43, 71.10, 69.65, 69.58, 67.91; **HRMS** (ESI^+) *m/z* calcd for $C_{14}H_{18}O_4$ $[M+Na]^+$: 273.1103, found 273.1102 $[M+Na]^+$.

S3

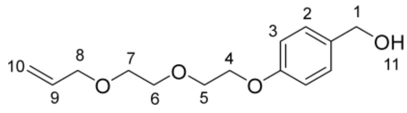

To a solution of compound **S2** (2.0 g, 8.0 mmol) in methanol was slowly added $NaBH_4$ (605 mg, 16 mmol) at 0 °C, after which the mixture was stirred for 1 h at room temperature. The reaction was quenched by slow addition of water while cooling the solution with an ice bath, and the reaction solution was extracted by CH_2Cl_2 (3 × 50 mL), and washed with water. The organic layer was dried over $MgSO_4$, filtered and concentrated under reduced pressure. Compound **S3** was obtained as a colorless oil (1.84 g, 92% yield) without further purification. **^{1}H NMR** (500 MHz, $CDCl_3$): δ 7.28 (d, J = 8.5 Hz, 2H, H_2), 6.91 (d, J = 8.5 Hz, 2H, H_3), 5.84-6.00 (m, 1H, H_9), 5.28 (dd, J = 21.5 Hz, 2.0 Hz, 1H, $H_{trans10}$), 5.18 (dd, J = 12.5 Hz, 1.5 Hz, 2H, H_{cis10}), 4.61 (d, J = 5.6 Hz, 1H, H_1), 4.11-4.17 (m, 2H, H_4), 4.04 (dt, J = 7.0 Hz, 1.5 Hz, 2H, H_8), 3.83-3.90 (m, 2H, H_5), 3.69-3.77 (m, 2H, H_6), 3.59-3.66 (m, 2H, H_7); **^{13}C NMR** (126 MHz, $CDCl_3$): δ 158.58, 134.86, 133.47, 128.73, 117.32, 114.86, 72.43, 71.03, 69.93, 69.60, 67.65, 65.19; **HRMS** (ESI^+) *m/z* calcd for $C_{14}H_{20}O_4$ $[M+Na]^+$: 275.1254, found 275.1265 $[M+Na]^+$.

S4

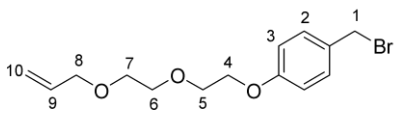

To a mixture of compound **S3** (1.0 g, 3.97 mmol), carbon tetrabromide (1.580 g, 4.76 mmol) and DIPEA (880 μL, 4.76 mmol) was injected anhydrous THF (100 mL), then triphenyl phosphine (1.040 g, 3.970 mmol) dissolved in anhydrous THF (50 ml) was slowly injected into the mixture at 0 °C. The mixture was slowly warmed to room temperature and stirred overnight under N_2 atmosphere. Afterwards, the reaction was quenched by addition of water (200 ml), and the reaction solution was extracted with ethyl acetate (3 × 100 mL) and washed with brine. The organic layer was dried over $MgSO_4$, filtered and concentrated under reduced pressure. The residue was purified with column chromatography (silica gel; hexane/ethyl acetate, 2:1), and compound **S4** was obtained as a colorless oil (947 mg, 76% yield). **^{1}H NMR** (500 MHz, $CDCl_3$): δ 7.31 (d, *J* = 8.5 Hz, 2H, H_2), 6.88 (d, *J* = 8.5 Hz, 2H, H_3), 5.85-5.98 (m, 1H, H_9), 5.28 (dd, *J* = 17.5 Hz, 1.5 Hz, 1H, $H_{trans10}$), 5.18 (dd, *J* = 10.5 Hz, 1.5 Hz, 1H, H_{cis10}), 4.5 (s, 2H, H_1), 4.10-4.16 (m, 2H, H_4), 4.03 (dt, *J* = 6.0 Hz, 1.5 Hz, 2H, H_8), 3.83-3.89 (m, 2H, H_5), 3.70-3.75 (m, 2H, H_6), 3.60-3.65 (m, 2H, H_7); **^{13}C NMR** (126 MHz, $CDCl_3$): δ 159.04, 134.85, 130.54, 130.26, 117.32, 115.04, 72.44, 71.04, 69.84, 69.60, 67.66, 34.06; **HRMS** (ESI^+) *m/z* calcd for $C_{14}H_{19}BrO_3$ $[M+Na]^+$: 337.0410 and 339.0390, found 337.0419 and 339.0401 $[M+Na]^+$.

S5

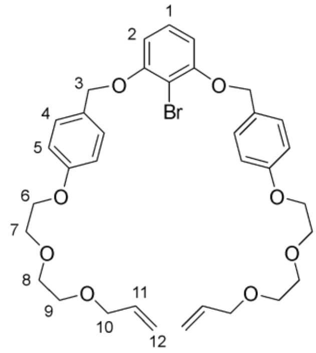

To a solution of compound **S4** (1.0 g, 3.185 mmol), in acetonitrile (30 mL) was added 2-bromoresorcinol (249 mg, 1.327 mmol) and K_2CO_3 (1.0 g, 7.962 mmol). The mixture was heated to reflux and stirred under N_2 atmosphere overnight. Afterwards, the reaction solution was filtered and washed with acetone (100 ml), and the filtrate was concentrated under vacuum. The obtained residue was purified with column chromatography (silica gel; hexane/ethyl acetate, 2:1), The compound **S5** was obtained as a liquid oil (626 mg, 72% yield). **^{1}H NMR** (500 MHz, $CDCl_3$): δ 7.38 (d, *J* = 8.5 Hz, 4H, H_4), 7.14 (t, *J* = 8.4 Hz, 1H, H_1), 6.93 (d, *J* = 8.5 Hz, 4H, H_5), 6.60 (d, *J* = 8.5 Hz, 2H, H_2), 5.85-5.99 (m, 2H, H_{11}), 5.28 (dd, *J* = 17.5 Hz, 2.0 Hz, 2H, $H_{trans12}$), 5.18 (dd, *J* = 10.5 Hz, 1.5 Hz, 2H, H_{cis12}), 5.08 (s, 4H, H_3), 4.10-4.18 (m, 4H, H_6), 4.04 (dt, *J* = 6.0 Hz, 1.5 Hz, 4H, H_{10}), 3.83-3.90 (m, 4H, H_7), 3.70-3.77 (m, 4H, H_8), 3.59-3.66 (m, 4H, H_9); **^{13}C NMR** (126 MHz, $CDCl_3$): δ 158.71, 156.62, 134.86, 129.05, 128.78, 128.75, 128.11, 117.34, 114.83, 107.04, 72.44, 71.03, 70.98, 69.92, 69.61, 67.61; **HRMS** (ESI^+) *m/z* calcd for $C_{34}H_{41}BrO_8$ $[M+Na]^+$: 679.1877 and 681.1857, found 679.1893 and 681.1881 $[M+Na]^+$.

1

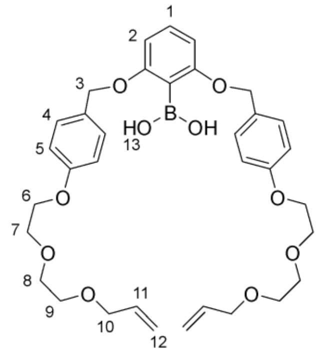

Compound **S5** (200 mg, 0.305 mmol) was dissolved in rigorously anhydrous tetrahydrofuran (5 mL, see general experimental for drying protocol) and cooled to –78 °C. *n*-butyllithium (146 μl, 2.5 M in hexanes, 1.2 mmol) was added dropwise and the mixture was stirred at –78 °C for another 30 min. Anhydrous trimethyl borate (68 μl, 0.610 mmol) was added and the mixture stirred for 16 h while warming to room temperature. After quenching by addition of water and ethyl acetate (50 mL each), the layers were separated. The aqueous layer was extracted with ethyl acetate (3 × 30 mL) and the combined organic layers were washed with brine (30 mL), dried with $MgSO_4$, filtered and concentrated. The remaining residue was purified with chromatography on silica gel (hexane/ethyl acetate 10:1 → 2:1), and the product was obtained as a transparent oil (124 mg, 65 %). **^{1}H NMR** (500 MHz, $CDCl_3$): δ 7.36 (*t*, *J* = 8.5 Hz, 1H, H_1), 7.33 (*d*, *J* = 8.5 Hz, 4H, H_4), 7.20 (s, 2H, H_{13}), 6.93 (*d*, *J* = 8.5 Hz, 4H, H_5), 6.71 (*d*, *J* = 8.5 Hz, 2H, H_2), 5.85-5.98 (m, 2H, H_{11}), 5.28 (dd, *J* = 21.5 Hz, 2.0 Hz, 2H, $H_{trans12}$), 5.18 (dd, *J* = 12.5 Hz, 1.5 Hz, 2H, H_{cis12}), 5.05 (*s*, 4H, H_3), 4.12-4.17 (*m*, 4H, H_6), 4.04 (dt, *J* = 6.0 Hz, 1.5 Hz, 4H, H_{10}), 3.83-3.90 (*m*, 4H, H_7), 3.70-3.77 (*m*, 4H, H_8), 3.59-3.67 (*m*, 4H, H_9); **^{13}C NMR** (126 MHz, $CDCl_3$): δ 171.30, 164.97, 159.28, 134.87, 133.02, 129.68, 127.87, 117.34, 115.20, 106.01, 72.45, 71.22, 71.03, 69.86, 69.60, 67.64; **^{11}B NMR** (160 MHz, $CDCl_3$): δ 29.2; **HRMS** (ESI$^+$) *m/z* calcd for $C_{34}H_{43}BO_{10}$ $[M+Na]^+$: 645.2841, found 645.2864 $[M+Na]^+$.

2

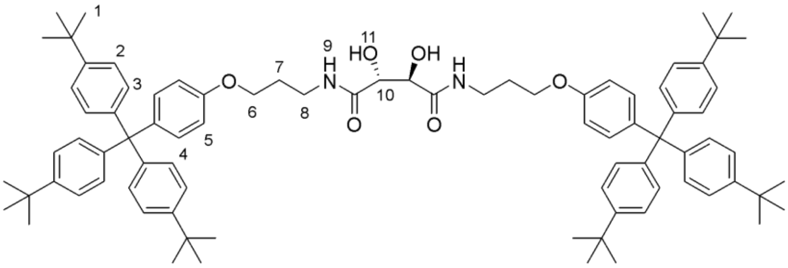

Compound **S10** (70 mg, 0.203 mmol) and compound **S9** (250 mg, 0.445 mmol) were dissolved in DMF (25 mL) and DIPEA (150 μl, 0.809 mmol) was added. The mixture was stirred at room temperature for overnight, after which the reaction mixture was concentrated under vacuum. The residue was extracted with ethyl acetate and washed with brine, and the combined organic layers were dried with Na_2SO_4, filtered and concentrated. The residue was purified with column chromatography (silica gel, hexane/ethyl acetate 1:1), and the product **2** was obtained as a white solid (175 mg, 70%). **^{1}H NMR** (500 MHz, $CDCl_3$): δ 7.49 (t, *J* = 6.0 Hz, 2H, H_9), 7.22 (d, *J* = 10.5 Hz, 12H, H_2), 7.06 (d, *J* = 9.0 Hz, 4H, H_4), 7.07 (d, *J* = 10.5 Hz, 12H, H_3), 6.74 (d, *J* = 9.0 Hz, 4H, H_5), 5.34-5.39 (m, 2H, H_{11}), 4.21-4.27 (m, 2H, H_{10}), 3.98 (t, *J* = 5.5 Hz, 4H, H_6), 3.38-3.51 (m, 4H, H_8), 1.89-2.00 (m, 4H,

H_7), 1.29 (s, 54H, H_1); **^{13}C NMR** (126 MHz, $CDCl_3$): δ 174.14, 156.45, 148.46, 144.24, 140.12, 132.47, 130.87, 124.19, 113.02, 70.29, 66.01, 63.20, 37.27, 34.43, 31.53, 28.94; **HRMS** (ESI$^+$) *m/z* calcd for $C_{84}H_{104}N_2O_6$ $[M+K]^+$: 1275.7526, found 1275.7536 $[M+K]^+$.

3

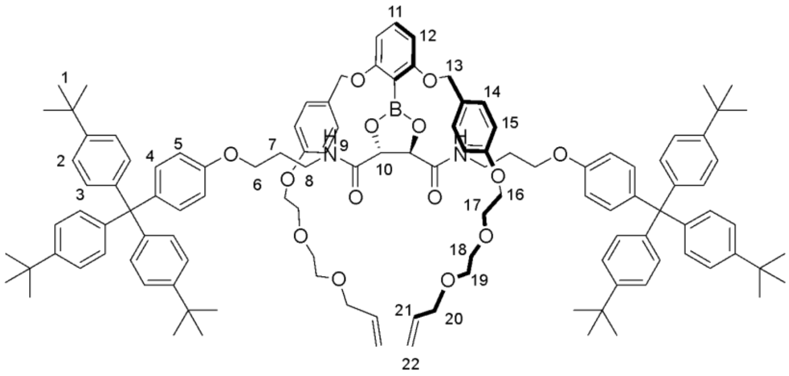

Compound **1** (15.0 mg, 0.024 mmol) and compound **2** (30 mg, 0.024 mmol) were dissolved in anhydrous toluene (2 mL) and stirred at room temperature for 24h, until the condensation equilibrium was reached. Condensation was monitored by ^{1}H NMR analysis, through withdrawal of reaction aliquots and sampling into $CDCl_3$ (integrity of the compound was confirmed through secondary analysis with other NMR solvents). After equilibrium was reached, the solvent was removed under vacuum. The obtained crude residue was directly used in next step without further purification. Through analysis via ^{1}H NMR spectroscopy, these optimized conditions yielded conversions of 70-89% of **3**, with variability between experiments. **^{1}H NMR** (500 MHz, $CDCl_3$): δ 7.23 (d, J = 8.5 Hz, 12H, H_2), 7.21 (d, J = 8.5 Hz, 4H, H_{14}), δ 7.10 (d, J = 8.5 Hz, 12H, H_3), 7.02 (d, J = 8.5 Hz, 4H, H_4), 7.02 (m, 2H, H_9), 6.96 (t, J = 8.5 Hz, 1H, H_{11}), 6.87 (d, J = 8.5 Hz, 4H, H_{15}), 6.61 (d, J = 8.5 Hz, 4H, H_5), 6.10 (d, J = 8.5 Hz, 2H, H_{12}), 5.85-5.96 (m, 2H, H_{21}), 5.26 (dd, J = 17.5 Hz, 2.0 Hz, 2H, $H_{trans22}$), 5.17 (dd, J = 10.5 Hz, 1.5 Hz, 2H, H_{cis22}), 4.94 (s, 2H, H_{10}), 4.66 (d, J = 10.5 Hz, 2H, H_{13}), 4.48 (d, J = 10.5 Hz, 2H, H_{13}), 4.09 (t, J = 4.5 Hz, 4H, H_{16}), 4.02 (dt, J = 5.5 Hz, 1.5 Hz, 4H, H_{20}), 3.82 (t, J = 4.5 Hz, 4H, H_{17}), 3.72-3.76 (m, 4H, H_6), 3.68-3.72 (m, 4H, H_{18}), 3.59-3.62 (m, 4H, H_{19}), 3.06-3.21 (m, 4H, H_8), 1.60-1.67 (m, 4H, H_7), 1.29 (s, 54H, H_1); **^{13}C NMR** (126 MHz, $CDCl_3$) δ 170.18, 164.41, 159.11, 156.62, 148.44, 144.33, 139.69, 138.00, 134.86, 132.37, 130.85, 129.57, 129.17, 125.44, 124.22, 117.31, 114.90, 113.08, 105.76, 79.14, 72.42, 71.03, 70.74, 69.85, 69.58, 67.68, 65.09, 63.18, 36.24, 34.44, 31.54, 29.85; **^{11}B NMR** (160 MHz, $CDCl_3$): δ 28.9.

4

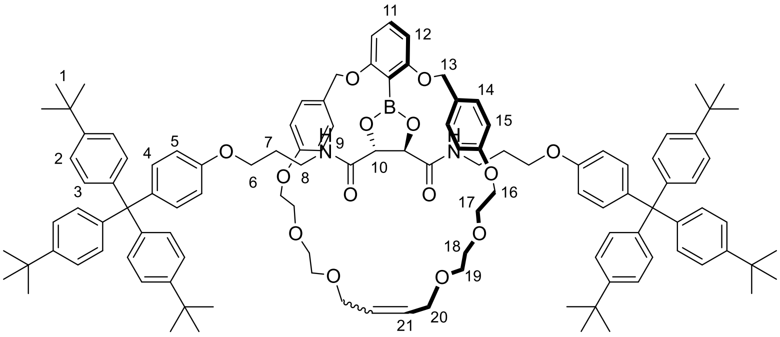

The crude mixture containing complex **3** (45 mg) from the protocol described above was redissolved into degassed CH_2Cl_2 (20 mL). Hoveyda-Grubbs 2nd generation catalyst (10% mol, 1.5 mg) in degassed CH_2Cl_2 (5 mL) was then injected into the reaction solution. The mixture was stirred under refluxing conditions for 24 h, after which the reaction was quenched by addition of vinyl acetate (2 mL). The solution was concentrated under vacuum, and the crude directly purified by chromatography (silica gel, hexane/ethyl acetate 1:1). Quasi[1]rotaxane **4** was then obtained as transparent oil (7.0 mg, 16% over two steps). Regioisomer ratio for the alkene metathesis was determined as E/Z 4:1 by inspection of the H_{20} proton region (E isomer appears at 5.79 – 5.77 ppm, Z isomer at 5.77 – 5.75 ppm). **^{1}H NMR** (500 MHz, $CDCl_3$): δ 7.25 (m, 2H, H_9), 7.24 (d, J = 8.5 Hz, 12H, H_2), 7.08 (d, J = 8.5 Hz, 12H, H_3), 6.84 (d, J = 8.5 Hz, 4H, H_{14}), 6.82 (d, J = 9.0 Hz, 4H, H_4), 6.75 (t, J = 8.0 Hz, 1H, H_{11}), 6.53 (d, J = 8.5 Hz, 4H, H_{15}), 6.41 (d, J = 9.0 Hz, 4H, H_5), 6.18 (d, J = 8.0 Hz, 2H, H_{12}), 5.79 – 5.75 (m, 2H, H_{21}, *E/Z* ≈ 4/1, see above), 4.84 (s, 2H, H_{10}), 4.67 (*d*, J = 11.5 Hz, 2H, H_{13}), 4.54 (*d*, J = 11.5 Hz, 2H, H_{13}), 3.96-3.99 (m, 4H, H_{20}), 3.93-3.96 (m, 2H, H_6) 3.84 (t, J = 5.0 Hz, 4H, H_{16}), 3.70-3.75 (m, 2H, H_6), 3.62-3.68 (m, 4H, H_{17}), 3.56-3.59 (m, 4H, H_{18}), 3.51-3.55 (m, 4H, H_{19}), 3.47-3.51 (m, 2H, H_8), 3.09-3.26 (m, 2H, H_8), 1.79-1.94 (m, 4H, H_7), 1.29 (s, 54H, H_1). **^{13}C NMR** (126 MHz, $CDCl_3$): δ 169.73, 162.58, 158.61, 156.28, 148.39, 144.39, 139.60, 132.06, 130.85, 129.97, 129.69, 129.38, 128.72, 124.19, 115.20, 114.52, 113.16, 108.15, 78.70, 71.59, 71.48, 70.94, 70.07, 69.64, 67.64, 66.60, 63.15, 37.58, 34.45, 31.57, 29.84; **HRMS** (ESI$^+$) *m/z* calcd for $C_{116}H_{139}BN_2O_{14}$ $[M+Na]^+$: 1818.0212, found 1818.0258 $[M+Na]^+$.

4'

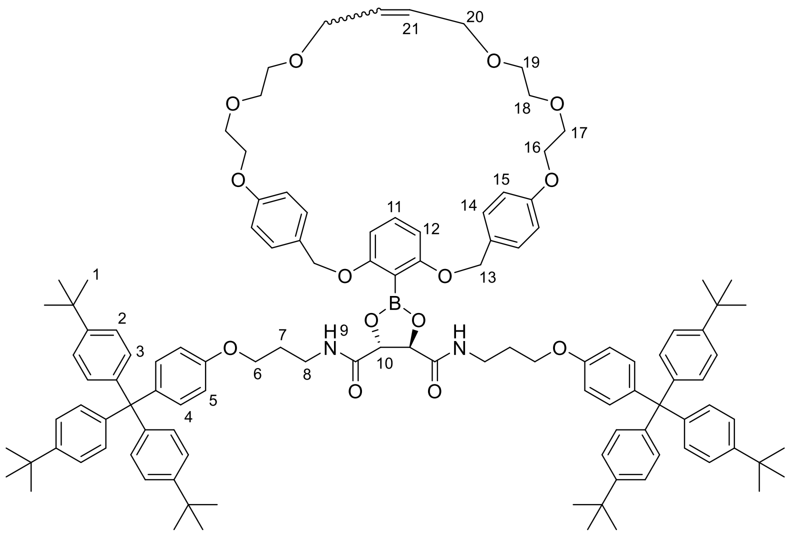

Through the procedure described for synthesis of compound **4** above, the non-interlocked isomer **4'** was also obtained as a white solid (15 mg, 33% mass balance over two steps, could only be isolated with impurities in the form of hydrolysis products **2** and **6**). This compounds could also be obtained by direct condensation of 1:1 ratio of macrocycle **6** and thread **2**. **^{1}H NMR** (500 MHz, $CDCl_3$): δ 7.48 (t, J = 6.0 Hz, 12H, H_9), 7.23 (d, J = 8.5 Hz, 12H, H_2), 7.07 (d, J = 8.5 Hz, 12H, H_3), 7.06 (d, J = 8.5 Hz, 4H, H_{14}), 7.03 (d, J = 9.0 Hz, 4H, H_4), 6.92 (t, J = 8.5 Hz, 1H, H_{11}), 6.79 (d, J = 8.5 Hz, 4H, H_{15}), 6.65 (d, J = 9.0 Hz, 4H, H_5), 6.19 (d, J = 8.5 Hz, 2H, H_2), 5.77 (t, J = 1.5 Hz, 2H, H_{21}), 5.06 (*s*, 2H, H_{10}), 4.94 (*s*, 4H, H_{13}), 4.02-4.07 (*m*, 2H, H_{16}), 3.97-4.01 (*m*, 4H, H_{20}), 3.93 (t, J = 6.0 Hz, 4H, H_6), 3.76-3.80 (*m*, 4H, H_{17}), 3.64-3.68 (*m*, 4H, H_{18}), 3.54-3.57 (*m*, 4H, H_{19}), 3.34-3.44 (*m*, 4H, H_8). 1.90-1.99 (*m*, 4H, H_7), 1.29 (*s*, 54H, H_1); **^{13}C NMR** (126 MHz, $CDCl_3$): 170.43, 162.60, 158.50, 156.53, 148.43, 144.27, 139.81, 137.98, 132.36, 130.84, 129.64, 129.16, 128.35, 125.43, 124.18, 114.97, 113.03, 105.95, 79.33, 71.40, 71.05, 69.79, 69.66, 67.69, 65.07, 63.17, 36.88, 34.42, 31.53, 29.84 ; **HRMS** (ESI^+) *m/z* calcd for $C_{116}H_{139}BN_2O_{14}$ $[M+Na]^+$: 1818.0212, found 1818.0258 $[M+Na]^+$.

6

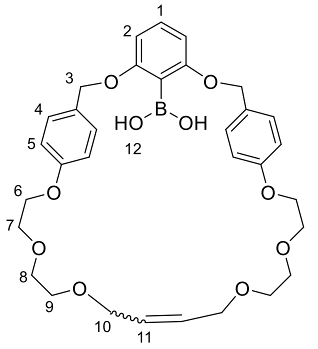

Through the procedure described for synthesis of compound **4** above, compound **6** was formed as a byproduct and could be separated out on the silica column as a transparent oil (3 mg, 23% over two steps). **^{1}H NMR** (500 MHz, $CDCl_3$): δ 7.36 (s, 2H, H_{12}), 7.22 (d, J = 8.5 Hz, 4H, H_4), 7.15 (t, J = 8.5 Hz, 1H, H_1), 6.86 (d, J = 8.5 Hz, 4H, H_5), 6.54 (d, J = 8.5 Hz, 2H, H_2), 5.77 (t, J = 1.5 Hz, H_{11}), 5.21 (s, 4H, H_3), 4.08-4.14 (m, 4H, H_6), 3.97-4.03 (m, 4H, H_{10}), 3.76-3.85 (m, 4H, H_7), 3.63-3.71 (m, 4H, H_8), 3.51-3.60 (m, 4H, H_9); **^{13}C NMR** (126 MHz, $CDCl_3$): δ 163.98, 158.90, 132.41, 129.73, 129.57, 129.22, 128.11, 115.16, 107.17, 71.44, 71.06, 70.57, 69.87, 69.65, 67.79; **HRMS** (ESI^+) *m/z* calcd for $C_{32}H_{39}BO_{10}$ $[M+Na]^+$: 617.2528, found 617.2584 $[M+Na]^+$.

7

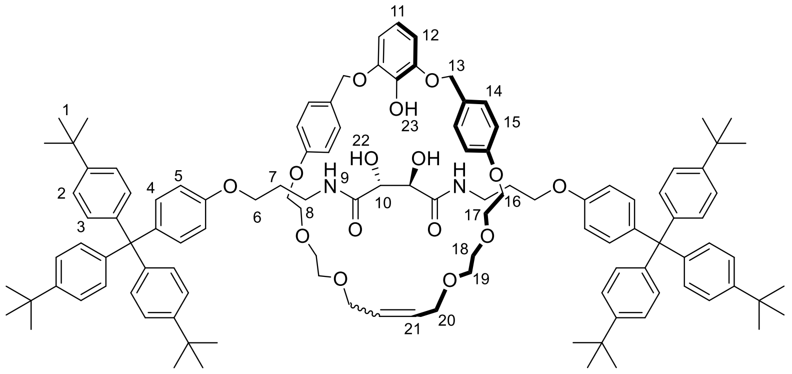

To a solution of compound **4** (1.8 mg, 0.001 mmol) in THF (200 μL) and water (200 μL) was added 30% H_2O_2 (aq, 2 μL), the mixture was stirred at room temperature for 3 h. After that, the reaction solution was extracted by ethyl acetate (50 ml x 3) and washed with brine. The combined organic layers were dried with Na_2SO_4, filtered and concentrated. The residue was purified with preparative silica gel TLC (CH_2Cl_2/MeOH 25:1) and the product was obtained as a white solid (1.2 mg, 67%). δ **^{1}H NMR** (500 MHz, $CDCl_3$): δ 7.76 (s, 1H, H_{23}), 7.23 (d, J = 8.5 Hz, 12H, H_2), 7.16-7.20 (m, 2H, H_9), 7.12 (d, J = 8.0 Hz, 4H, H_{14}), 7.09 (d, J = 8.5 Hz, 12H, H_3), 7.01 (d, J = 8.5 Hz, 4H, H_4), 6.64 (d, J = 8.0 Hz, 4H, H_{15}), 6.55 (d, J = 8.5 Hz, 4H, H_5), 6.40-6.47 (m, 3H, H_{11}, H_{12}), 6.06-5.99 (m, 2H, H_{22}), 5.72 (t, J = 3.0 Hz, 2H, H_{21}, E/Z ≈ 3/1), 4.98 (s, 4H, H_{13}), 4.26 (d, J = 7.5 Hz, 2H, H_{10}), 3.85-3.94 (m, 8H, H_{16}, H_{20}), 3.67 (t, J = 5.0 Hz, 4H, H_{17}), 3.58-3.62 (m, 4H, H_6), 3.54-3.58 (m, 4H, H_{18}), 3.45-3.50 (m, 4H, H_{19}), 3.09-3.20 (m, 4H, H_8), 1.61-1.66 (m, 4H, H_7), 1.29 (s, 54H, H_1). **^{13}C NMR** (126 MHz, $CDCl_3$): δ 175.59, 172.62, 158.58, 156.53, 148.43, 146.17, 144.35, 139.71, 137.28, 132.26, 130.86, 130.17, 129.67, 124.20, 118.83, 114.69, 113.16, 108.67, 71.63, 71.39, 71.35, 70.83, 69.82, 69.70, 67.47, 65.69, 63.20, 36.87, 34.44, 31.55, 29.85; **HRMS** (ESI$^+$) *m/z* calcd for $C_{116}H_{142}N_2O_{15}$ $[M+Na]^+$: 1827.0336, found 1827.0340 $[M+Na]^+$.

8

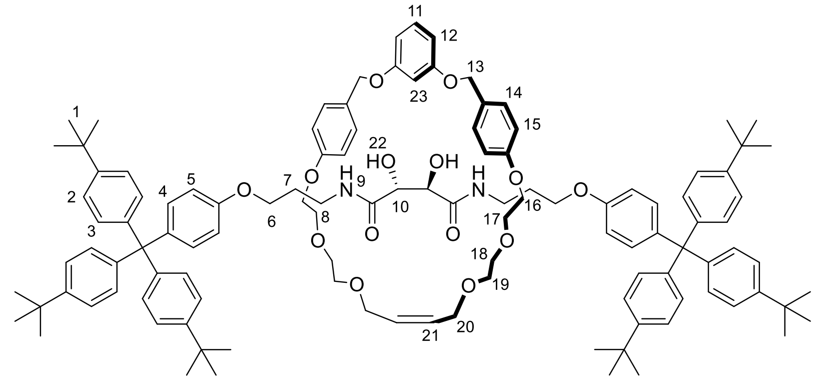

To a solution of compound **4** (5.0 mg, 0.003 mmol) in CH_2Cl_2 (0.5 mL) and MeOH (1 mL) was added copper(II) acetate (0.5 mg, 0.003 mmol) and the mixture was stirred at 55 °C for 16 h. After that, the reaction solution was extracted by CH_2Cl_2 (30 ml x 3) and washed with brine. The combined organic layers were dried with Na_2SO_4, filtered and concentrated. The residue was purified with preparative silica gel TLC (CH_2Cl_2/MeOH 25:1) and the product was obtained as a white solid (4.0 mg, 82%). **^{1}H NMR** (500 MHz, $CDCl_3$): δ 7.22 (d, J = 9.0 Hz, 12H, H_2), 7.06-7.11(m, 16H, H_{14}, H_3), 6.99-7.01 (m, 5H, H_4, H_{11}), 6.70 (d, J = 8.5 Hz, 4H, H_{15}), 6.58 (d, J = 9.0 Hz, 4H, H_5), 6.44 (dd, J = 8.0, 2.0 Hz, 2H, H_{12}), 6.24 (t, J = 2.5 Hz, 1H, H_{23}), 5.71 (t, J = 3.0 Hz, 2H, H_{21}, E/Z ≈ 3/1), 4.89 (s, 4H, H_{13}), 4.31-4.35 (m, 2H, H_{10}), 3.93 (t, J = 5.0 Hz, 4H, H_{16}), 3.86-3.89 (m, 4H, H_{20}), 3.68 (t, J = 5.0 Hz, 4H, H_{17}), 3.59-3.64 (m, 4H, H_6), 3.55-3.59 (m, 4H, H_{18}), 3.45-3.50 (m, 4H, H_{19}), 3.09-3.21 (m, 4H, H_8), 1.62-1.66 (m, 4H, H_7), 1.29 (s, 54H, H_1). **^{13}C NMR** (126 MHz, $CDCl_3$): δ 172.60, 159.77, 158.31, 156.54, 148.43, 144.35, 139.69, 132.25, 130.85, 130.17, 129.89, 129.53, 128.70, 124.20, 114.80, 113.17, 107.35, 103.35, 71.70, 71.37, 70.84, 69.87, 69.72, 69.64, 67.43, 65.67, 63.20, 36.90, 34.44, 31.54, 29.85; **HRMS** (ESI$^+$) m/z calcd for $C_{116}H_{142}N_2O_{14}$ [M+Na]$^+$: 1811.0392, found 1811.0384 [M+Na]$^+$.

9

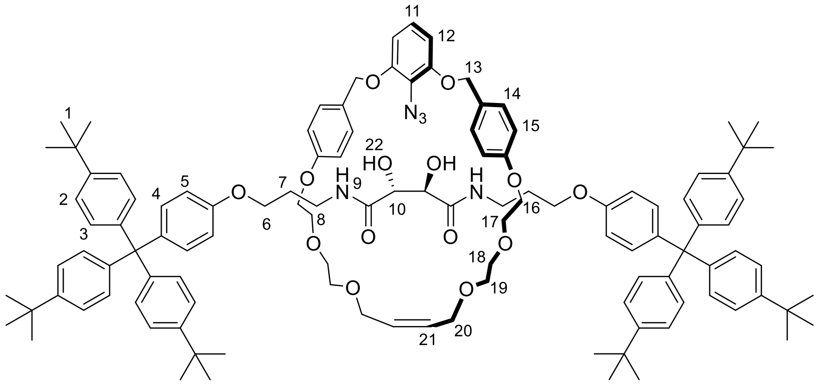

To a solution of compound **4** (5.0 mg, 0.003 mmol) in DMF (1.5 mL) was added copper(II) acetate (0.5 mg, 0.003 mmol), and sodium azide (0.3 mg, 0.004 mmol), after which the mixture was stirred at 55 °C for 16 h. The reaction solution was extracted with ethyl acetate and washed with brine (30 ml x 5). The combined organic layers were dried with Na_2SO_4, filtered and concentrated. The residue was purified with preparative silica gel TLC (CH_2Cl_2/MeOH 25:1) and the product was obtained as a white solid (4.0 mg, 78%). **^{1}H NMR** (500 MHz, $CDCl_3$): 7.23 (d, J = 8.5 Hz, 12H, H_2), 7.12

(d, J = 8.5 Hz, 4H, H_{14}), 7.09 (d, J = 8.5 Hz, 12H, H_3), 7.02 (d, J = 9.0 Hz, 4H, H_4), 6.62 (d, J = 8.5 Hz, 4H, H_{15}), 6.56 (t, J = 8.5 Hz, 1H, H_{11}), 6.53 (d, J = 9.0 Hz, 4H, H_5), 6.32 (d, J = 8.5 Hz, 2H, H_{12}), 5.69 (t, J = 3.0 Hz, 2H, H_{21}, E/Z ≈ 3/1), 5.09 (s, 4H, H_{13}), 4.40 (d, J = 4.0 Hz, 2H, H_{10}), 3.79-3.88 (m, 8H, H_{20}, H_{16}), 3.63-3.68 (m, 4H, H_{17}), 3.52-3.57 (m, 4H, H_{18}), 3.42-3.51 (m, 8H, H_6, H_{19}), 3.05-3.15 (m, 4H, H_8), 1.50-1.54 (m, 4H, H_7), 1.29 (s, 54H, H_1). **^{13}C NMR** (126 MHz, $CDCl_3$): δ 172.59, 158.50, 156.45, 151.49, 148.44, 144.35, 139.76, 132.24, 130.85, 130.17, 129.89, 129.43, 129.23, 128.73, 124.22, 114.75, 113.13, 107.24, 71.72, 71.28, 70.80, 70.06, 69.71, 67.39, 65.74, 63.20, 36.07, 34.45, 31.54, 29.85. **HRMS** (ESI$^+$) *m/z* calcd for $C_{116}H_{141}N_5O_{14}$ [M+Na]$^+$: 1852.0405, found 1852.0396 [M+Na]$^+$.

10

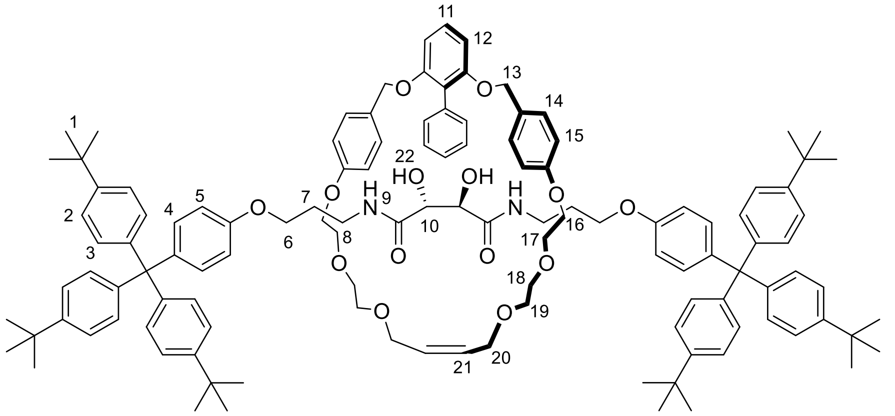

To a solution of compound **4** (5.0 mg, 0.003 mmol) in DMF (1.5 mL) was added bromobenzene (1.2 mg, 0.009 mmol), RuPhos (0.1 mg), $Pd_2(dba)_3$ (0.1 mg) and sodium *tert*-butoxide (0.8 mg, 0.009 mmol), and the mixture was stirred at at 110 ℃ for 24h under N_2 atmosphere. After that, the reaction solution was extracted by ethyl acetate and washed with brine (30 ml x 5). The combined organic layers were dried with Na_2SO_4, filtered and concentrated. The residue was purified with preparative silica gel TLC (CH_2Cl_2/MeOH 25:1). The product could only be obtained as a white solid in a mixture with the protodeboronation side product **8** in a 55:45 ratio (2.5 mg, 35% yield of **10**). **^{1}H NMR** (500 MHz, $CDCl_3$, based on mixture with product **8**): δ 7.30-7.40 (m, 5H, H_{23}, H_{24}, H_{25}), 7.23 (d, J = 9.0 Hz, 12H, H_2), 7.10 (d, J = 9.0 Hz, 12H, H_3), 7.07 (d, J = 9.0 Hz, 4H, H_{14}), 7.04 (d, J = 9.0 Hz, 4H, H_4), 6.99 (d, J = 9.0 Hz, 4H, H_{15}), 6.78 (*t, J* = 8.5 Hz, 1H, H_{11}), 6.56 (d, J = 9.0 Hz, 4H, H_5), 6.39 (d, J = 9.0 Hz, 2H, H_{12}), 5.66-5.70 (m, 2H, H_{21}, E/Z ≈ 3/1), 4.96 (s, 4H, H_{13}), 4.16 (d, J = 7.5 Hz, 2H, H_{10}), 3.82-3.86 (m, 4H, H_{16}), 3.74-3.81 (m, 4H, H_{20}), 3.62-3.66 (m, 4H, H_{17}), 3.51-3.56 (m, 4H, H_{18}), 3.58-3.62 (m, 4H, H_6), 3.40-3.46 (m, 4H, H_{19}), 3.05-3.13 (m, 4H, H_8), 1.61-1.66 (m, 4H, H_7), 1.29 (s, 54H, H_1). **HRMS** (ESI$^+$) *m/z* calcd for $C_{122}H_{146}N_2O_{14}$ [M+Na]$^+$: 1886.0666, found 1886.0686 [M+Na]$^+$.

Macrocycle cleavage from compound 4

To a solution compound **4** (2.0 mg, 0.001 mmol) in THF (150 μL) and water (150 μL) was added 2N NaOH aqueous solution (60 μL) and 30% H_2O_2 (aq, 100 μL), and the mixture was stirred at room temperature for 1 h. After that, the reaction solution was quenched by adding 2N HCl aq (60 μL), extracted with ethyl acetate (20 ml x 3) and washed with brine. The combined organic layers were dried with Na_2SO_4, filtered and concentrated. The residue was purified with preparative silica gel TLC (CH_2Cl_2/MeOH 25:1) and thread **2** was recycled as a white solid (1.2 mg, 87%). The cleaved ring residue could not be isolated, but was identified as **S11** from HRMS and NMR analysis of the crude mixture (which showed a 1:1 ratio between **S11** and thread **2**). **^{1}H NMR** (500 MHz, $CDCl_3$) of **S11**: δ 7.28 (d, *J* = 8.0 Hz, 4H, H_3), 6.90 (d, *J* = 8.0 Hz, 4H, H_4), 5.79 (m, 2H, H_{10}), 4.61 (s, 4H, H_2), 4.13 (t, *J* = 4.5 Hz, 4H, H_5), 4.02 (m, 4H, H_9), 3.86 (m, 4H, H_6), 3.72 (m, 4H, H_7), 3.61 (m, 4H, H_8). **HRMS** (ESI^+) *m/z* calcd for $C_{26}H_{36}O_8$ $[M+Na]^+$: 499.2308, found 499.2303 $[M+Na]^+$.

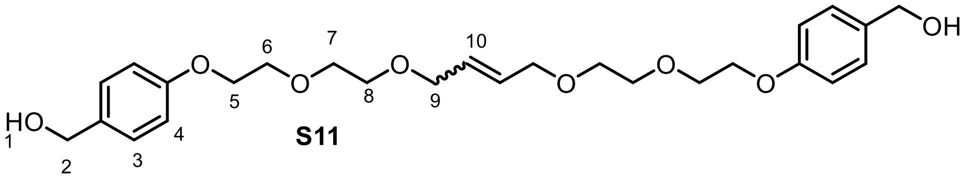

S11

S5. SUPPLEMENTARY DATA

5.1. Screening of conditions for boronic ester condensation

Table S1. Condensation experiments between pincer ligand **1** and thread **2**.[a]

Entry	Solvent	T (°C)	c (mM)	Desiccant	24 h conversion[b]	48 h conversion[b]
1	toluene	r.t.	10	/	67%	70%
2	1,4-dioxane	r.t.	10	/	59%	52%
3	CH_2Cl_2	r.t.	10	/	58%	60%
4	THF	r.t.	10	/	54%	56%
5	THF	r.t.	1	/	40%	48%
6	THF	r.t.	5	/	49%	55%
8	THF	r.t.	30	/	57%	57%
9	toluene	40	10	/	65%	63%
10	toluene	80	10	/	N/A[c]	N/A[c]
11	toluene	r.t.	10	Na_2SO_4	61%	63%
12	toluene	r.t.	10	4Å MS	67%	64%

[a] Conditions: **1** (0.002 mmol), **2** (0.002 mmol), solvent (200 μL), r.t. [b] Conversion obtained by ^{1}H NMR analysis of a reaction aliquot added into $CDCl_3$ (0.55 mL). [c] No complex **3** observed due to boroxazine formation.

5.2. Hydrolysis of condensation complex 3

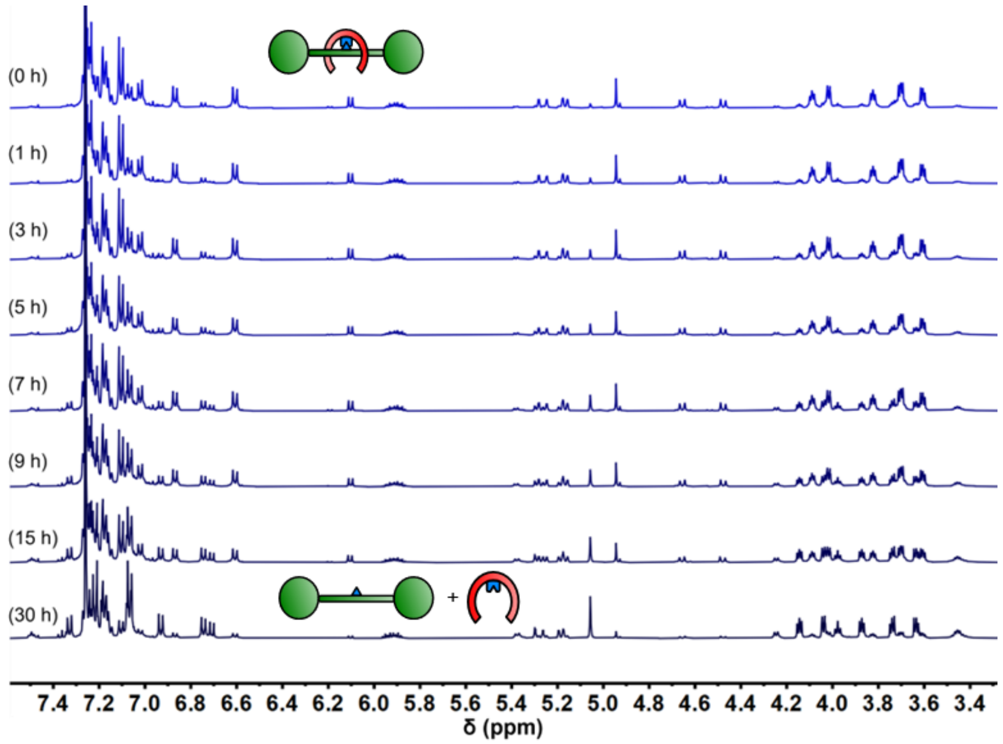

Figure S1. Partial ^{1}H NMR spectra (500 MHz, $CDCl_3$, 298 K) of the gradual hydrolysis of condensation complex **3** when incubated in water-saturated $CDCl_3$ after the time indicated next to each spectrum**.**

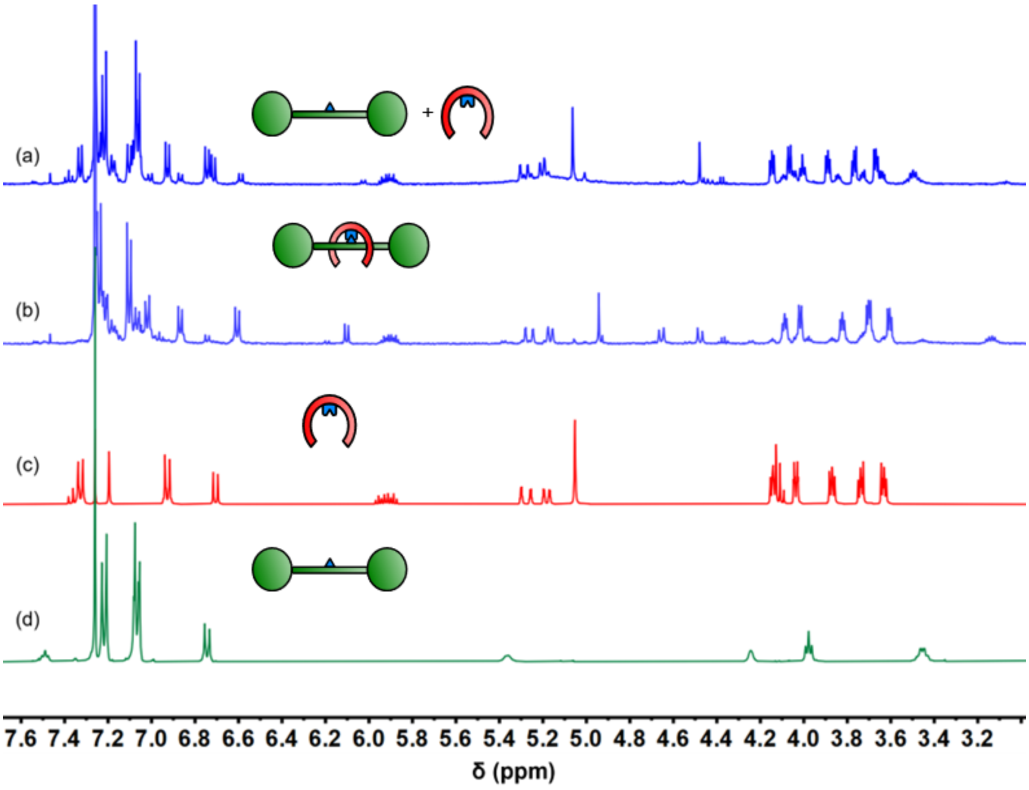

Figure S2. Partial ^{1}H NMR spectra (500 MHz, $CDCl_3$, 298 K) showing (a) immediate hydrolysis of condensation complex **3** after adding 5 eq CF_3COOH (b) condensation complex **3** before hydrolysis (c) boronic acid pincer **1,** (d) diol thread **2**.

5.3. RCM crude mixture analysis

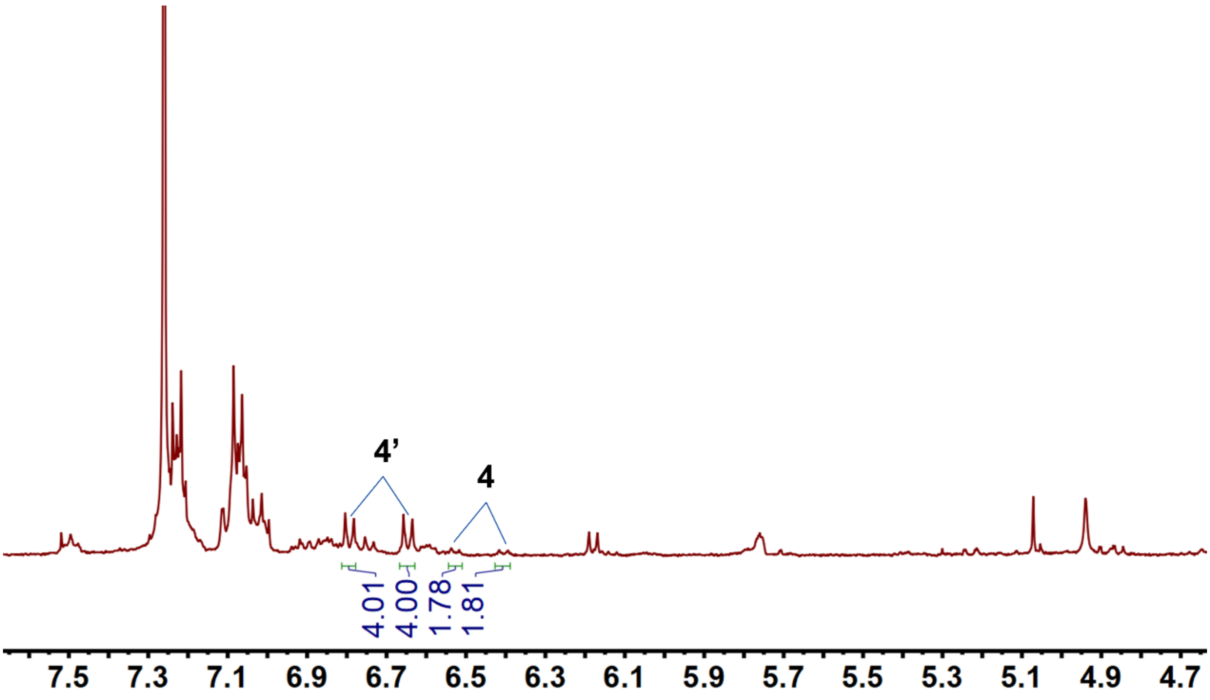

Figure S3. Partial ^{1}H NMR spectra (500 MHz, $CDCl_3$, 298 K) of crude reaction mixture after RCM on condensation complex **3**.

5.4. Hydrolysis of quasi[1]rotaxane 4

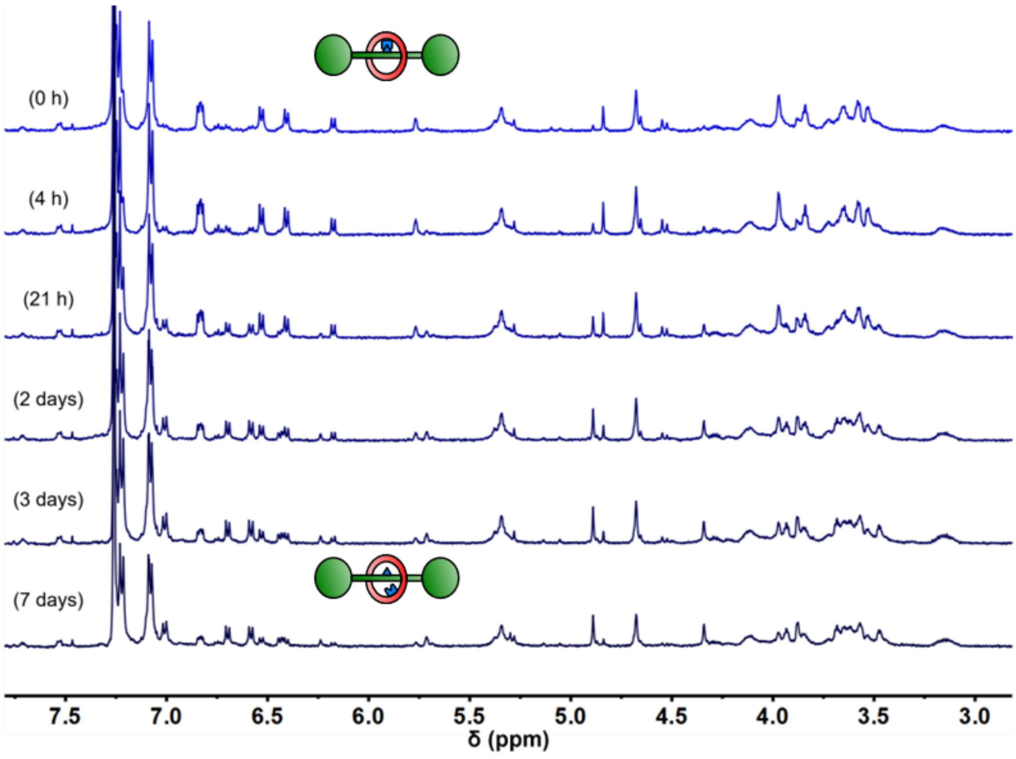

Figure S4. Partial ^{1}H NMR spectra (500 MHz, $CDCl_3$, 298 K) of the gradual hydrolysis of quasi[1]rotaxane **4** when incubated in water-saturated $CDCl_3$ after the time indicated next to each spectrum.

5.5. Hydrolysis of non-interlocked isomer 4’

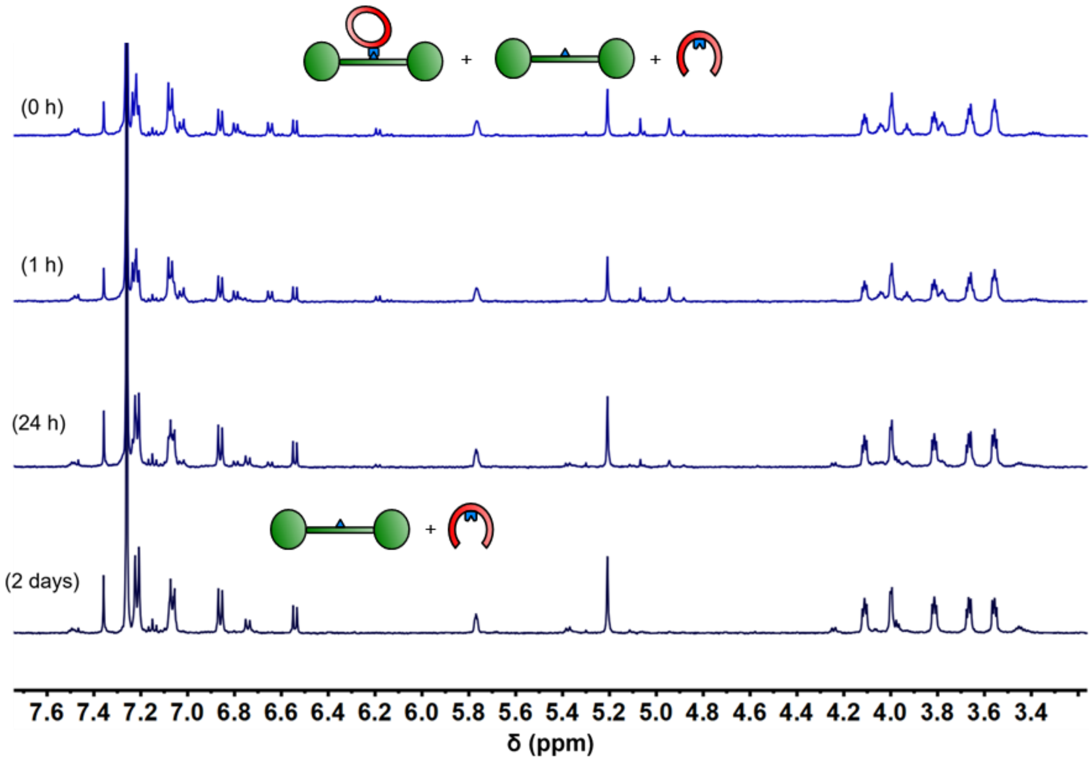

Figure S5. Partial ^{1}H NMR spectra (500 MHz, $CDCl_3$, 298 K) of hydrolysis of the non-interlocked isomer **4’** (isolated as a mixture together with hydrolysis products **1** and **2**) after incubation in water-saturated $CDCl_3$ for the time indicated next to each spectrum**.**

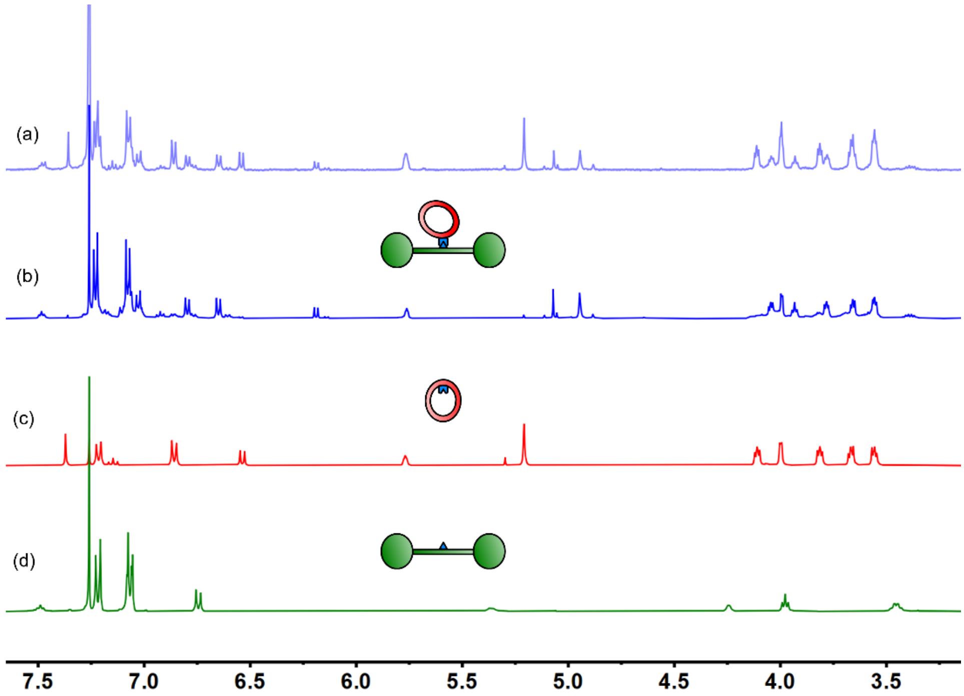

Figure S6. Partial ^{1}H NMR spectra (500 MHz, $CDCl_3$, 298 K) of (a) the non-interlocked isomer **4’** (isolated as a mixture together with hydrolysis products **1** and **2**), (b) the non-interlocked isomer **4’** formed by condensation of **6** and **2** in anhydrous toluene for 24h, (c) boronic acid macrocycle **6**, and (d) thread **2**.

5.6. Macrocycle cleavage from compound 4

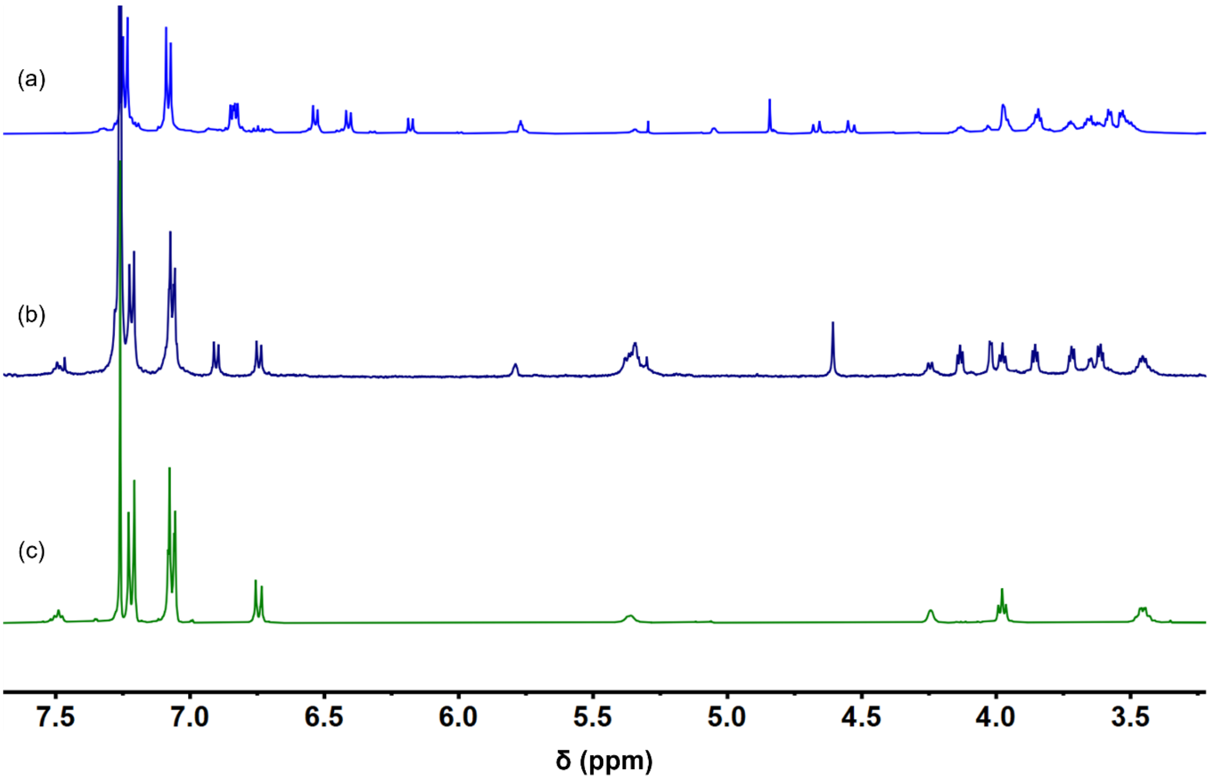

Figure S7. Partial ^{1}H NMR spectra (500 MHz, $CDCl_3$, 298 K) of (a) quasi[1]rotaxane **4**, (b) crude mixture obtained after treatment of **4** with NaOH and H_2O_2, (c) diol thread **2.**

5.7. TW-IMS measurements

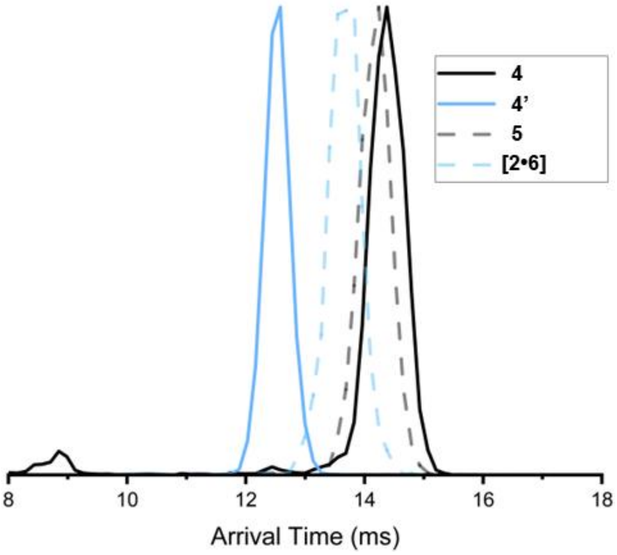

Figure S8. TW-IMS spectra showing arrival times of the four main species **4**, **4’**, **5** and [**2**•**6**]. See general experimental section for measurement details.

Table S2. Summary of TW-IMS arrival times.

Compound	Arrival time (ms)
**4**	14.4
**4’**	14.3
**5**	12.5
**[2•6]**	13.7

5.8. CID measurements

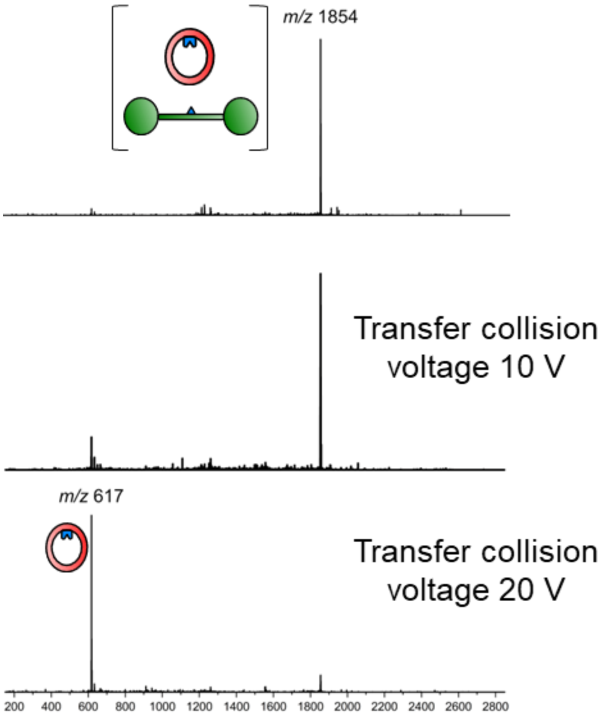

Figure S9. MS/MS (+ESI) spectra of [**2•6**], with mass selection *m/z* 1854 (top) and after application of transfer collision voltages of 10 V (middle) and 20 V (bottom) to fragment the parent ion.

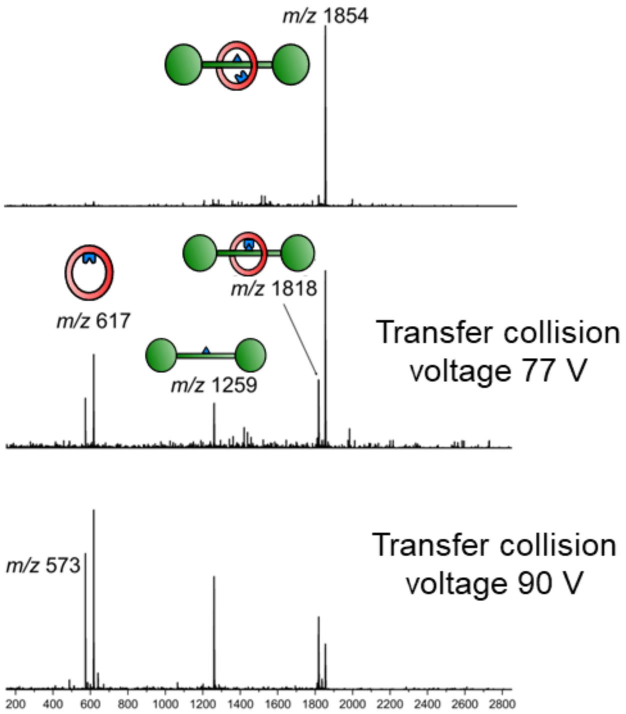

Figure S10. MS/MS (+ESI) spectra of [**5**], with mass selection *m/z* 1854 (top) and after application of transfer collision voltages of 77 V (middle) and 90 V (bottom) to fragment the parent ion.

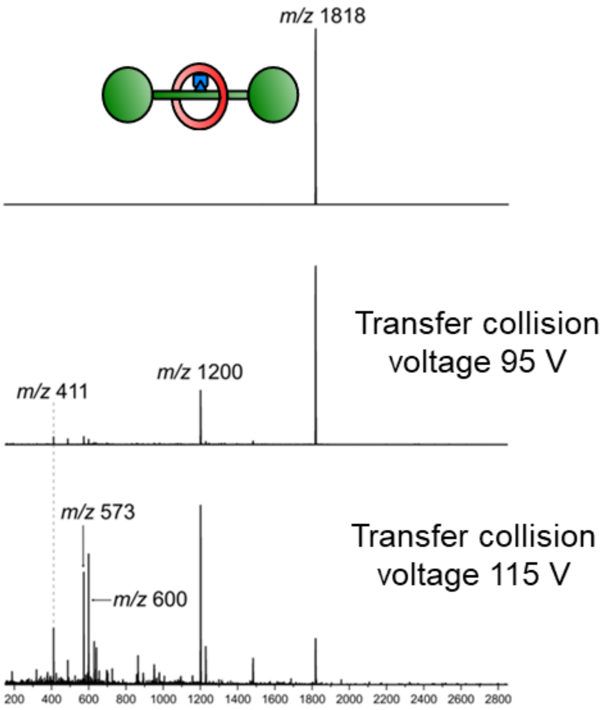

Figure S11. MS/MS (+ESI) spectra of [**4**], with mass selection *m/z* 1854 (top) and after application of transfer collision voltages of 95 V (middle) and 115 V (bottom) to fragment the parent ion.

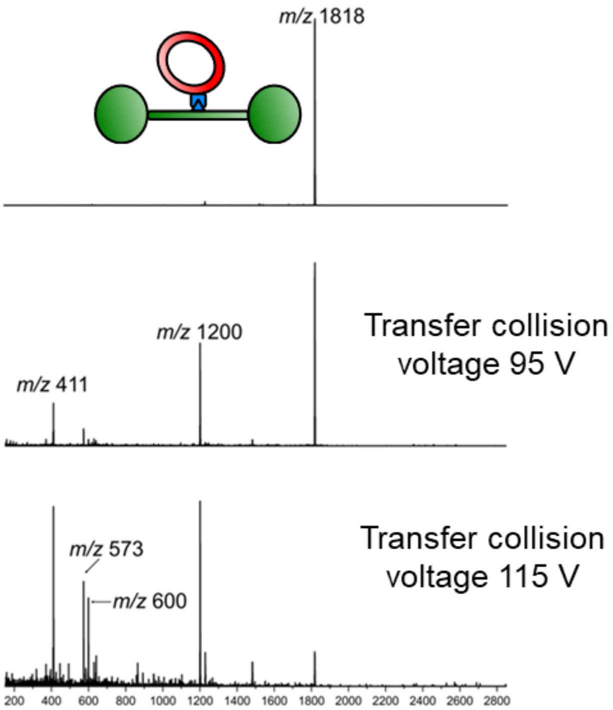

Figure S12. MS/MS (+ESI) spectra of [**4'**], with mass selection *m/z* 1854 (top) and after application of transfer collision voltages of 95 V (middle) and 115 V (bottom) to fragment the parent ion.

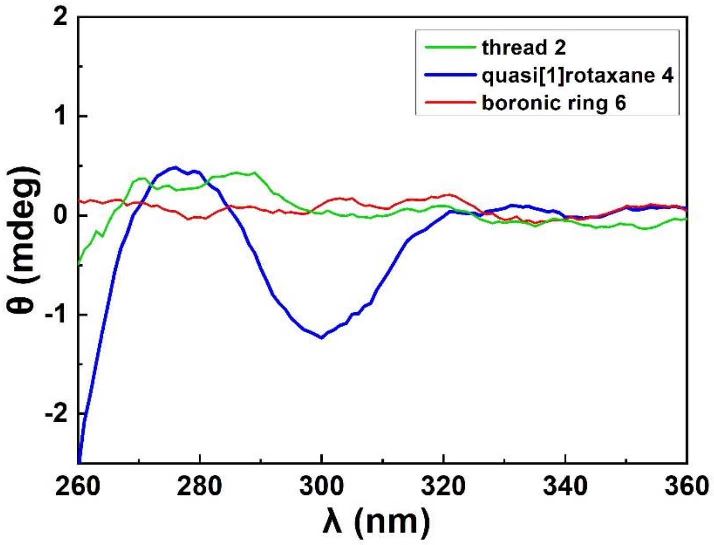

Figure S13. CD spectra (1×10^{-4} M, CH_2Cl_2, 298 K, smoothed) of thread **2**, quasi[1]rotaxane **4** and boronic acid ring **6**.

S6. MASS SPECTRA

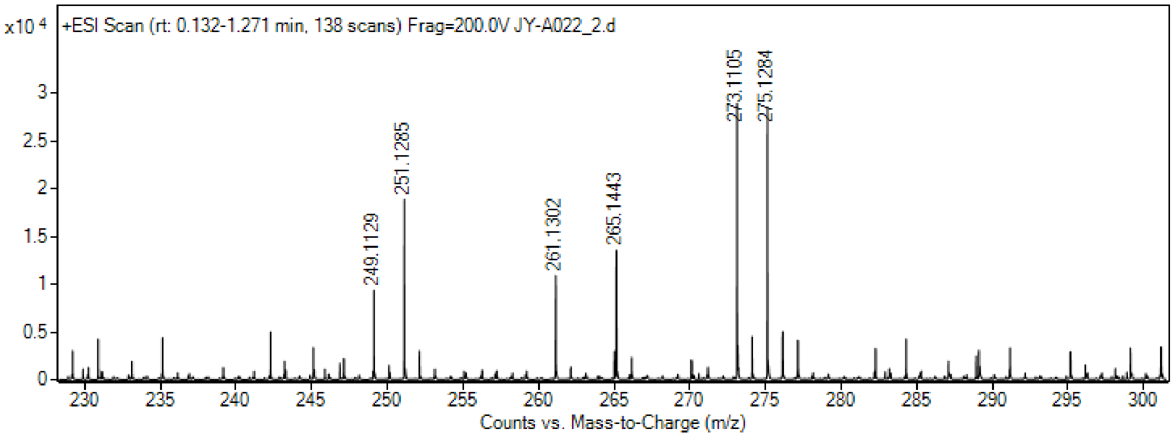

Spectrum S1. High-resolution ESI-MS (positive mode) of **S2**.

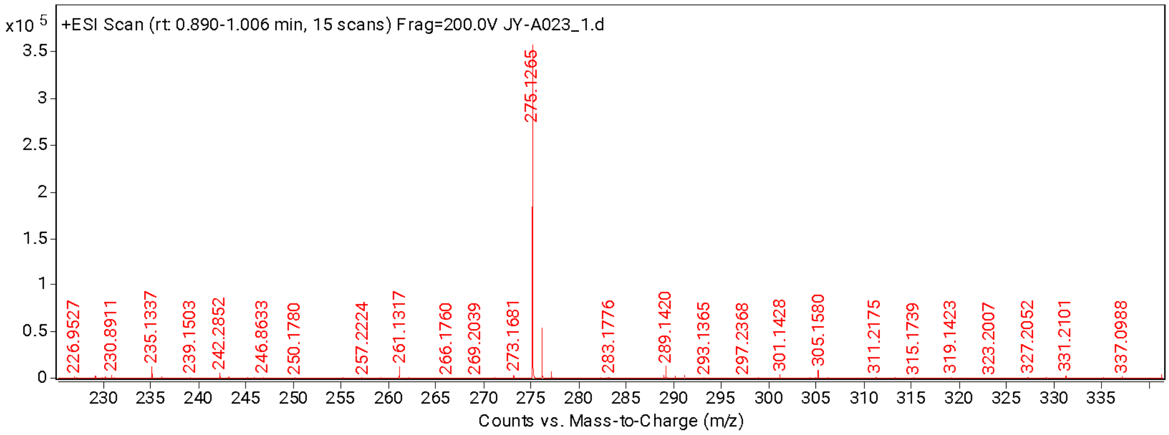

Spectrum S2. High-resolution ESI-MS (positive mode) of **S3**.

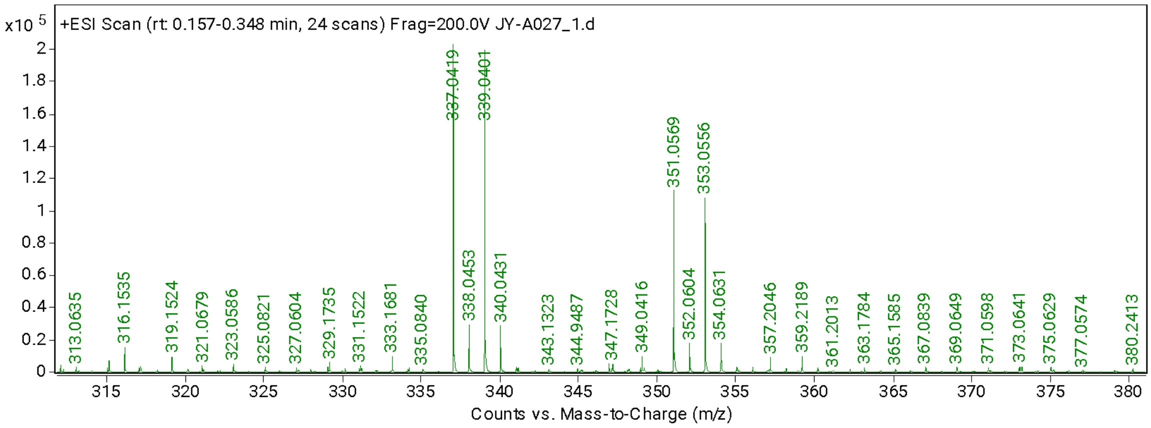

Spectrum S3. High-resolution ESI-MS (positive mode) of **S4**.

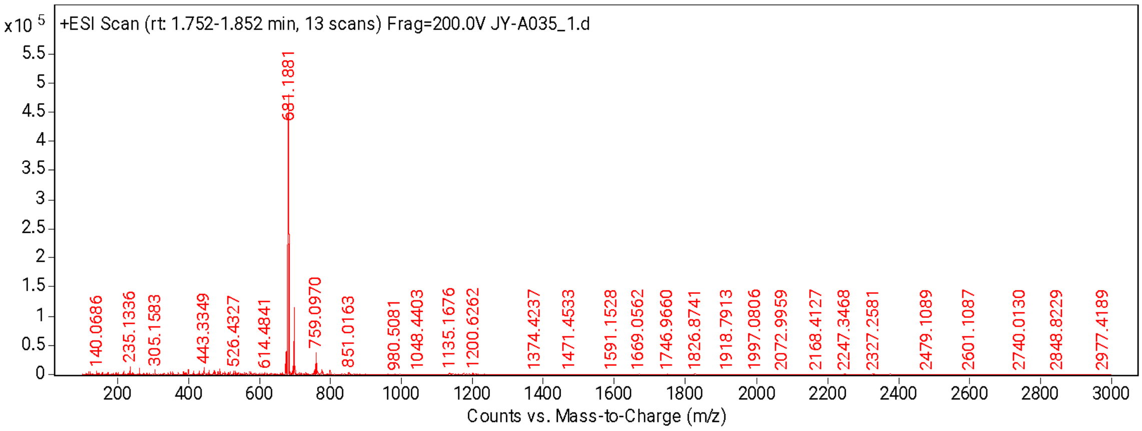

Spectrum S4. High-resolution ESI-MS (positive mode) of **S5**.

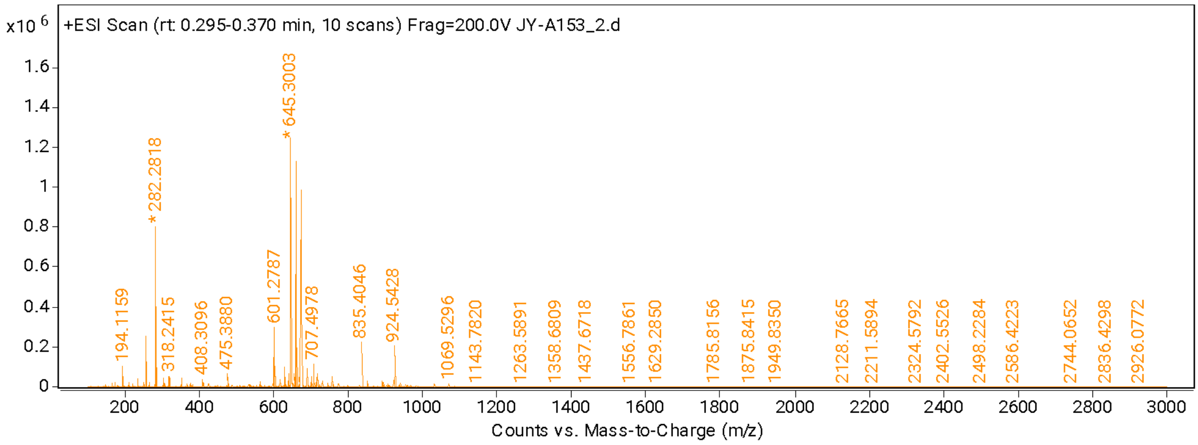

Spectrum S5. High-resolution ESI-MS (positive mode) of **1**.

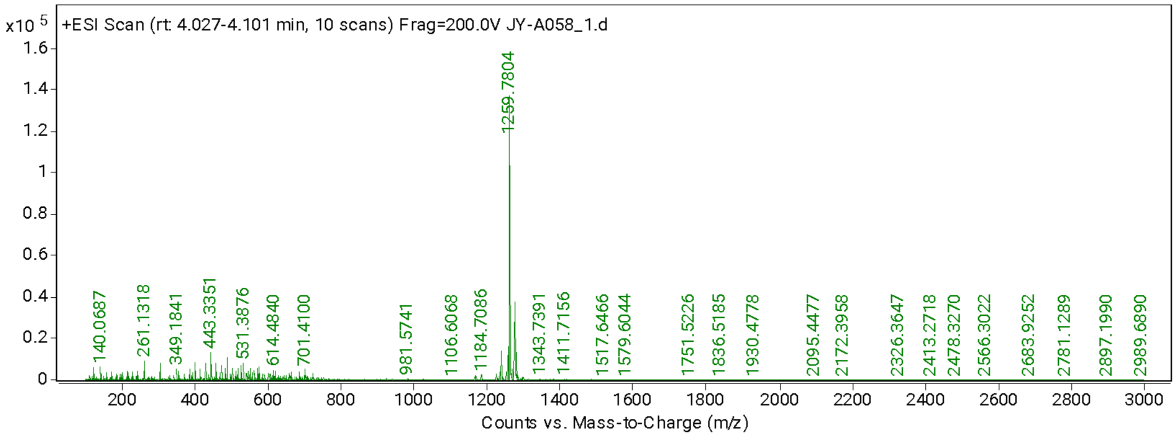

Spectrum S6. High-resolution ESI-MS (positive mode) of **2**.

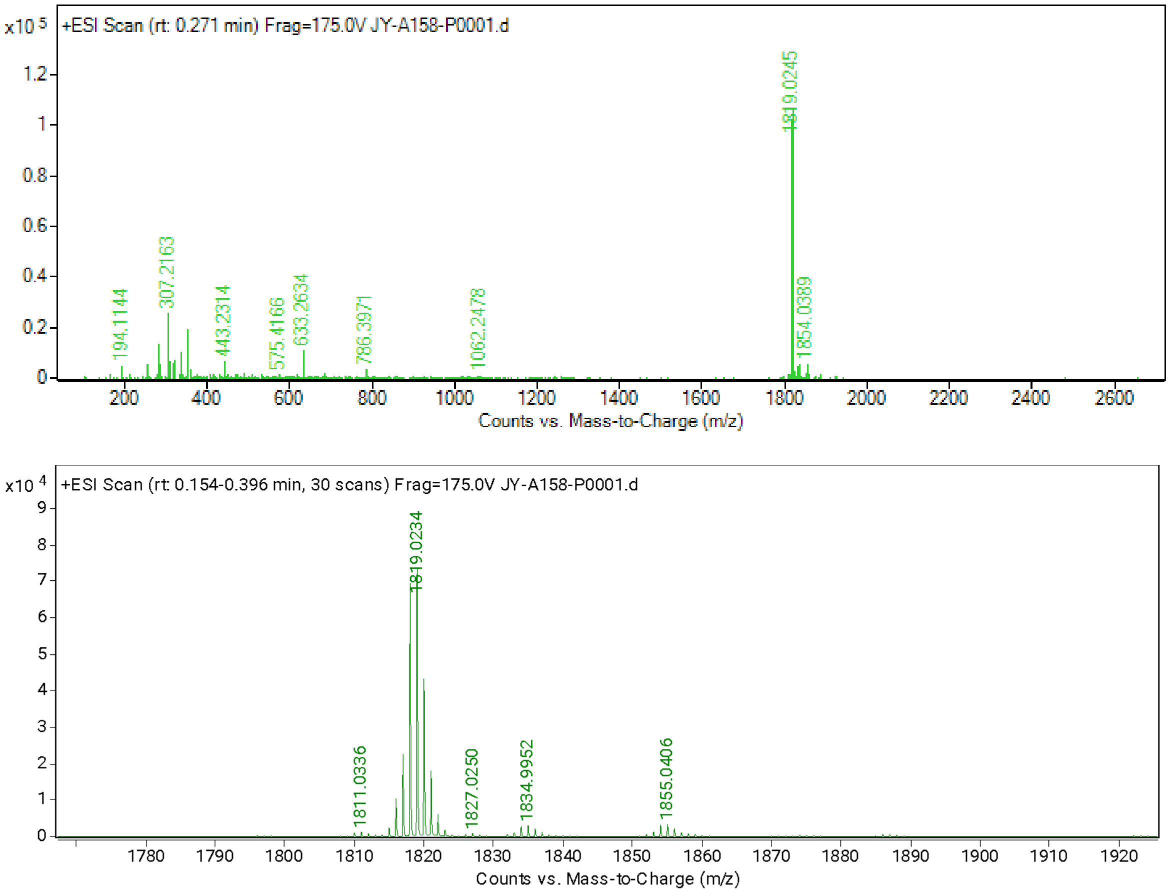

Spectrum S7. High-resolution ESI-MS (positive mode) of **4** (top: full spectrum; bottom: zoom).

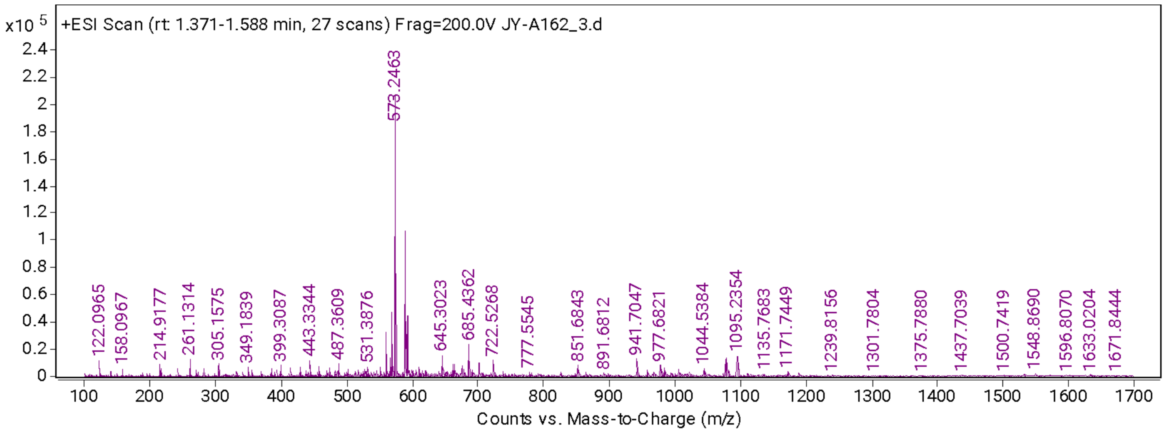

Spectrum S8. High-resolution ESI-MS (positive mode) of **6**.

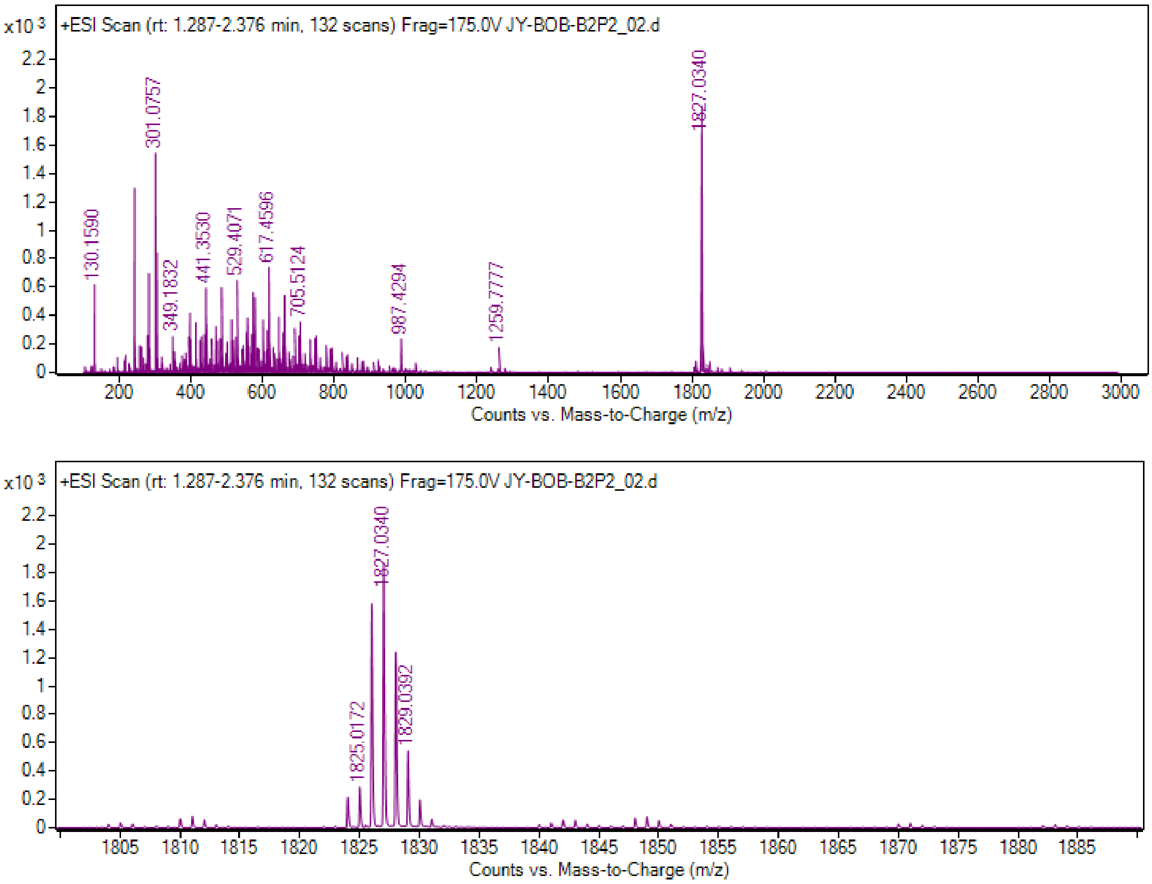

Spectrum S9. High-resolution ESI-MS (positive mode) of **7** (top: full spectrum; bottom: zoom).

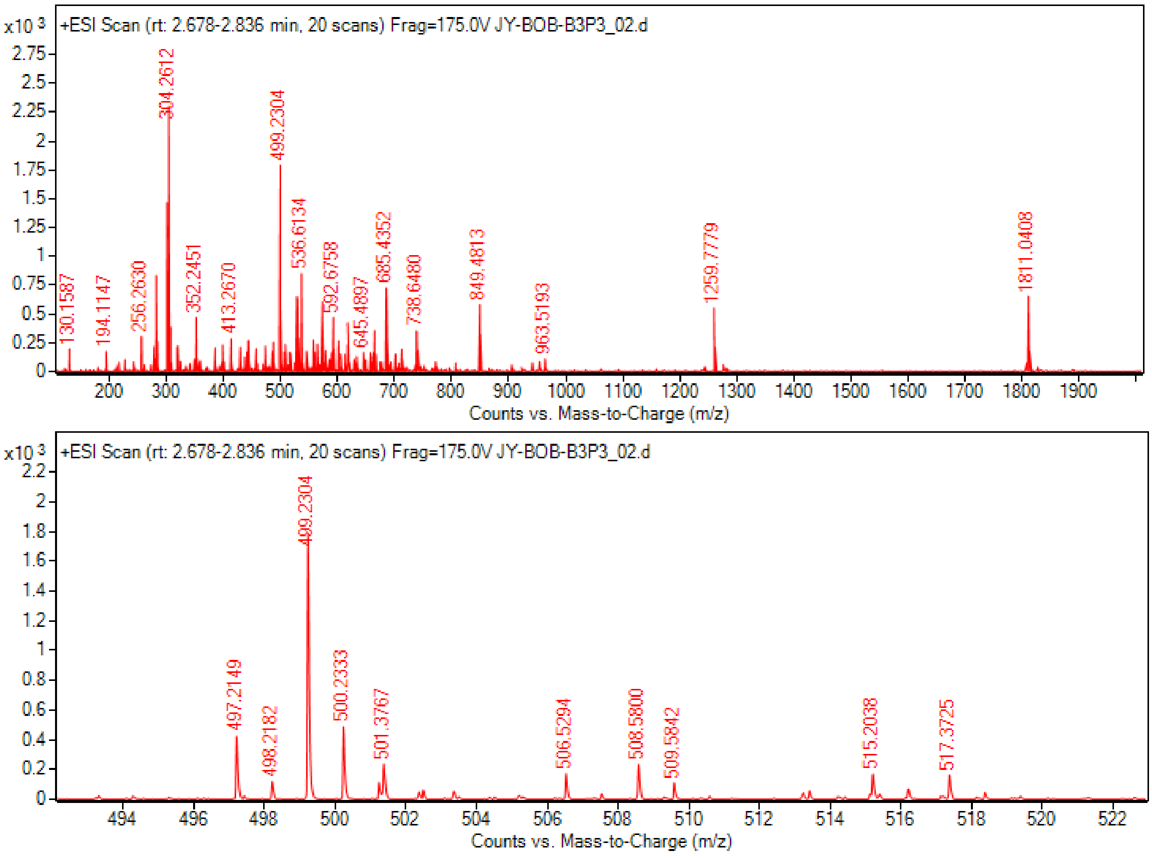

Spectrum S10. High-resolution ESI-MS (positive mode) of ring residue **S11** (top: full spectrum; bottom: zoom).

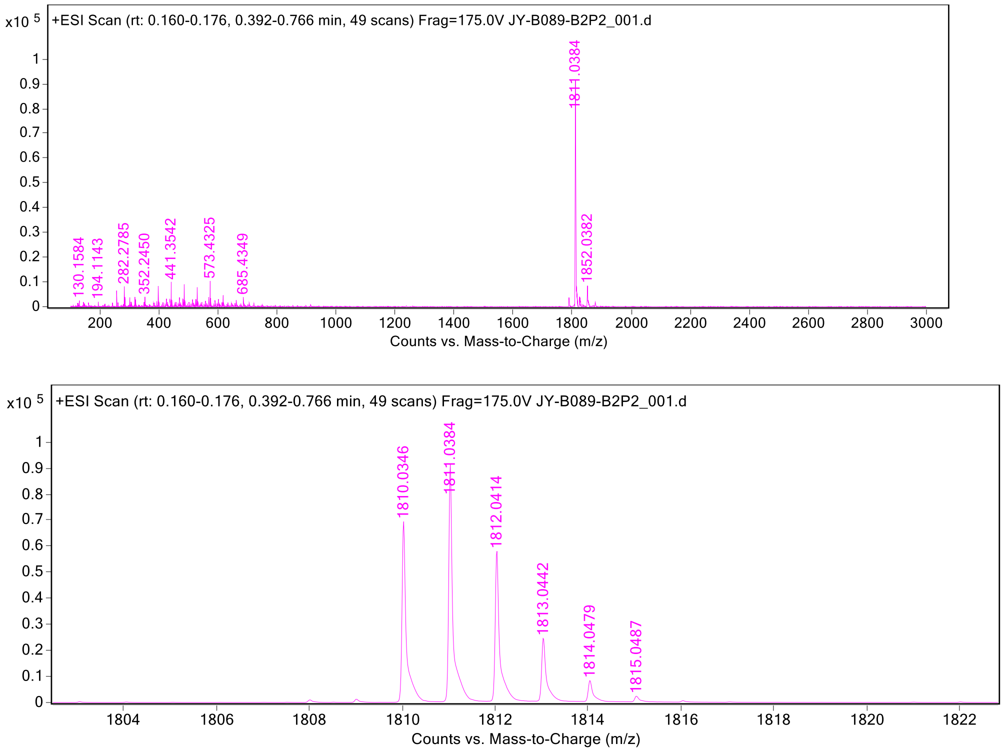

Spectrum S11. High-resolution ESI-MS (positive mode) of **8** (top: full spectrum; bottom: zoom).

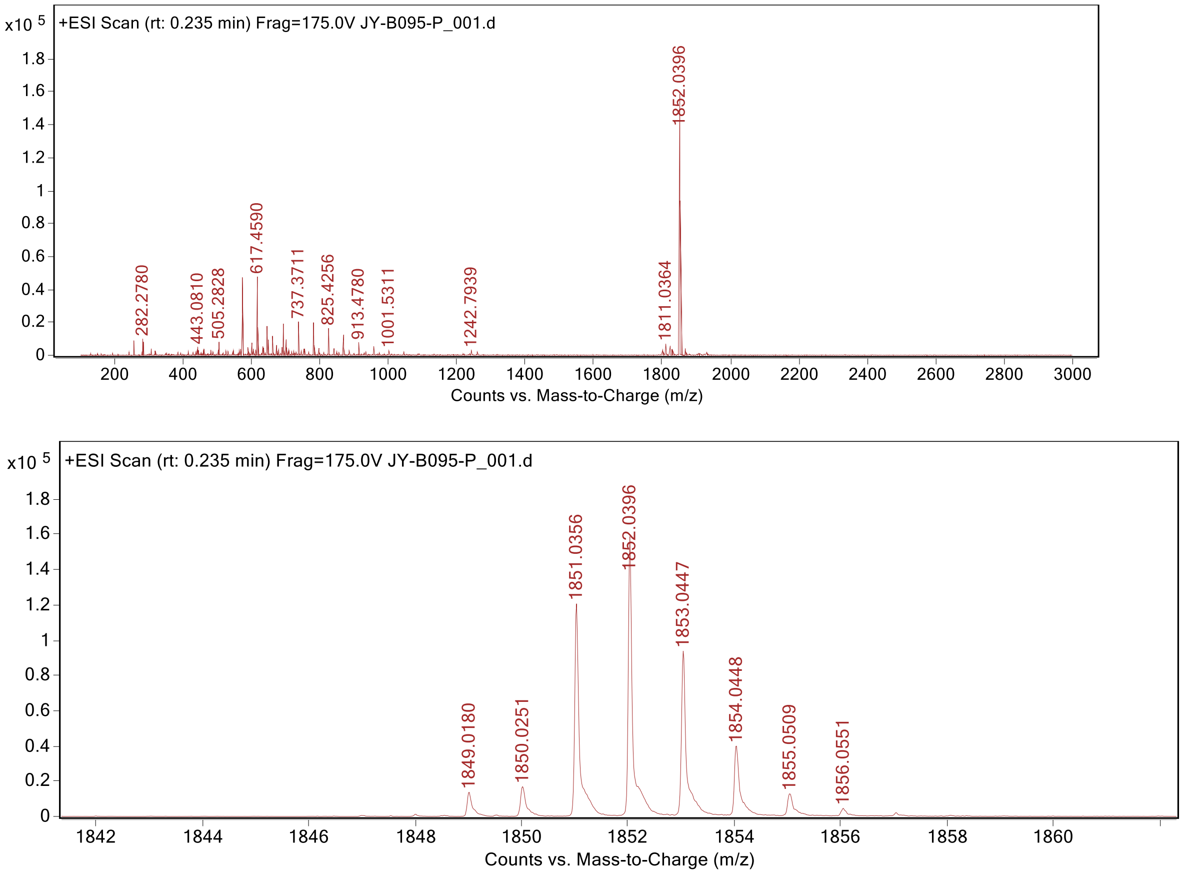

Spectrum S12. High-resolution ESI-MS (positive mode) of **9** (top: full spectrum; bottom: zoom).

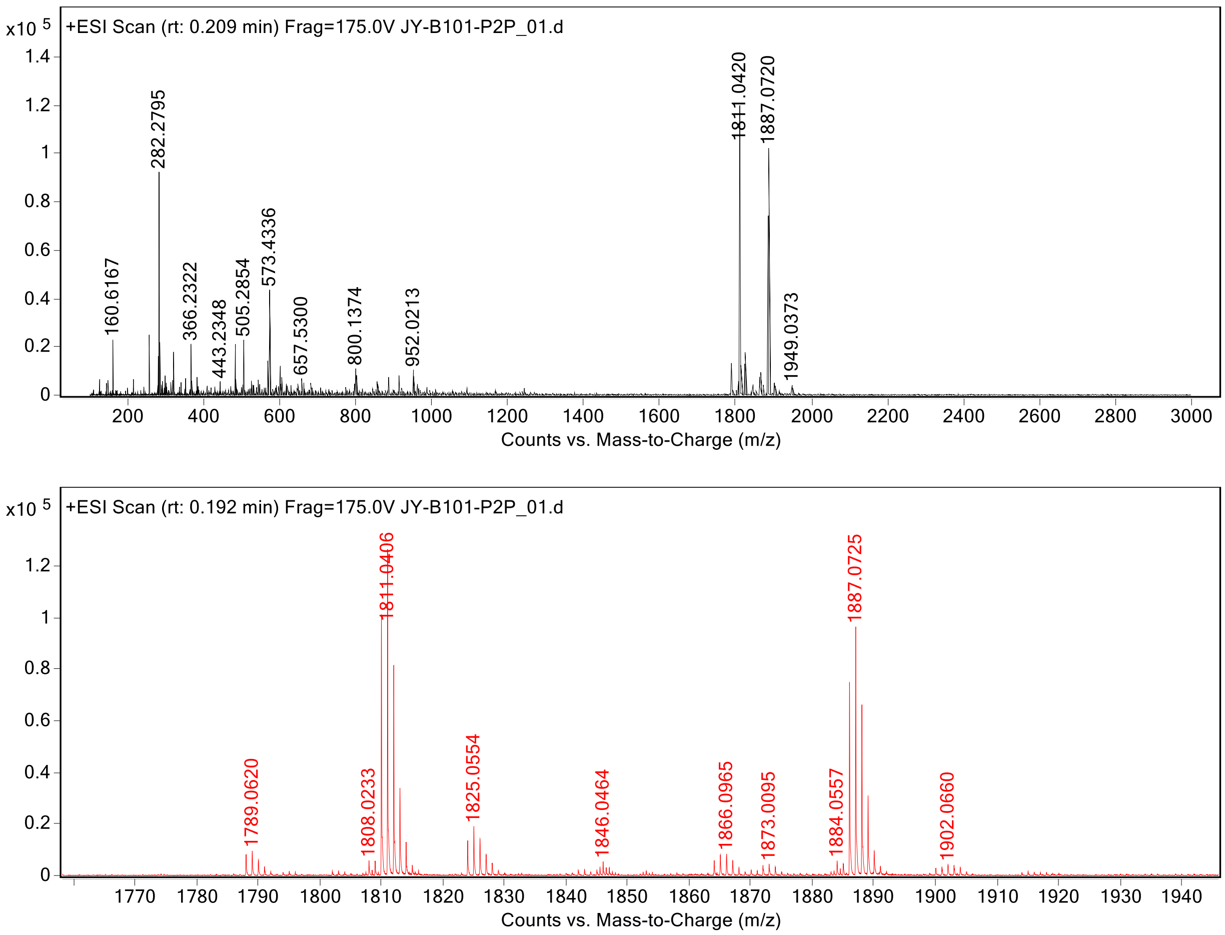

Spectrum S13. High-resolution ESI-MS (positive mode) of **10** (top: full spectrum; bottom: zoom).

S7. NMR SPECTRA

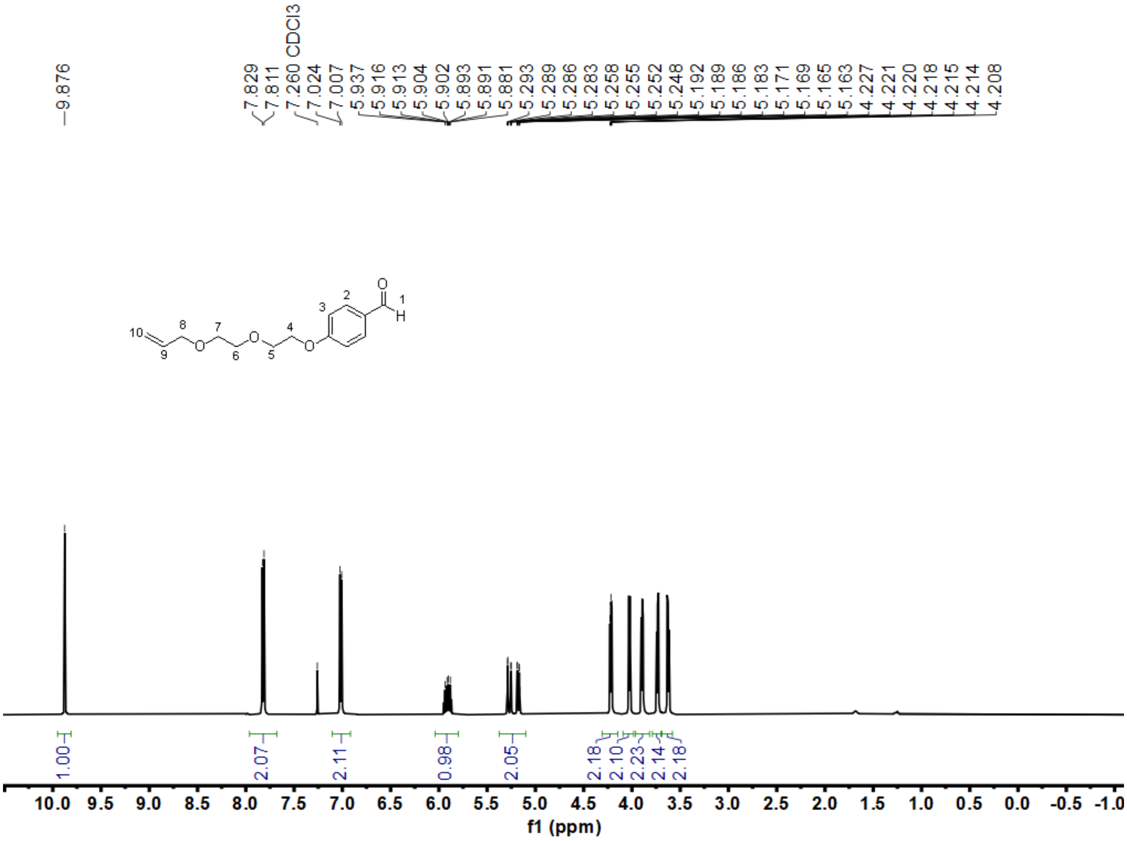

Spectrum S14. ^{1}H NMR spectrum (500 MHz, $CDCl_3$, 298 K) of **S2**.

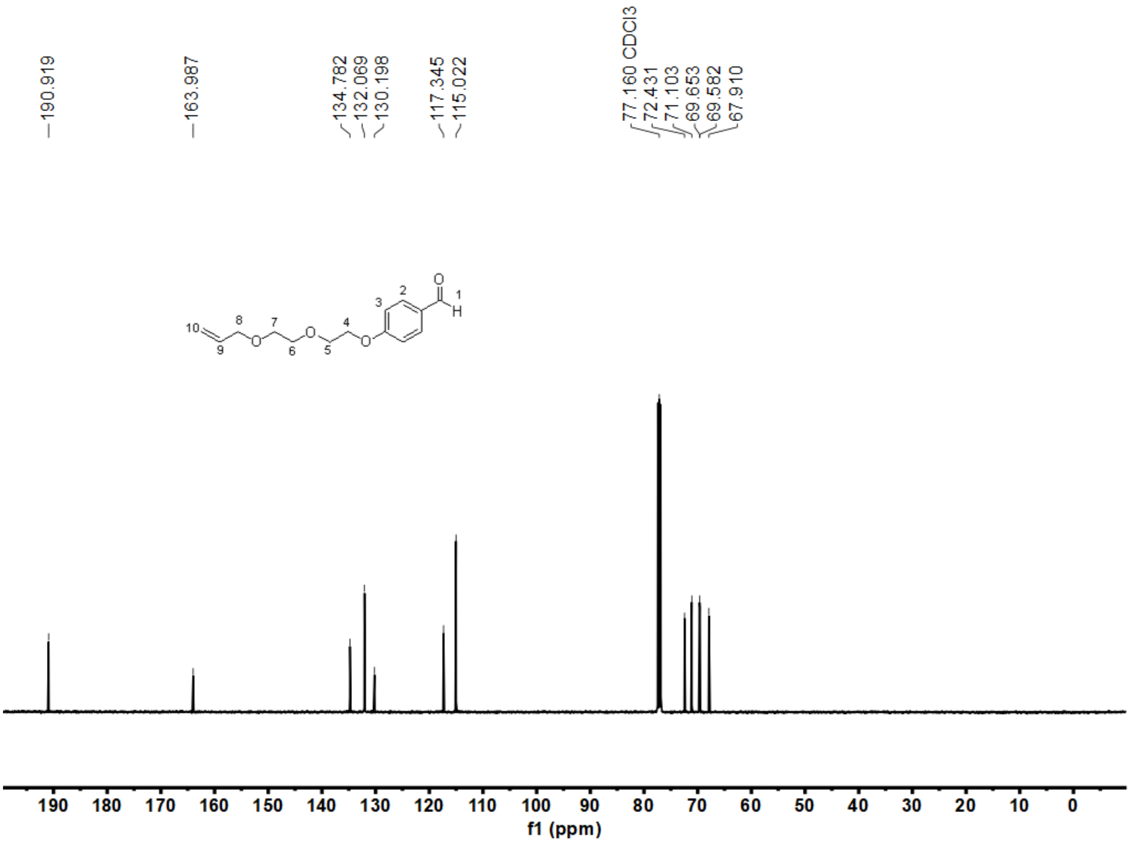

Spectrum S15. ^{13}C NMR spectrum (126 MHz, $CDCl_3$, 298 K) of **S2**.

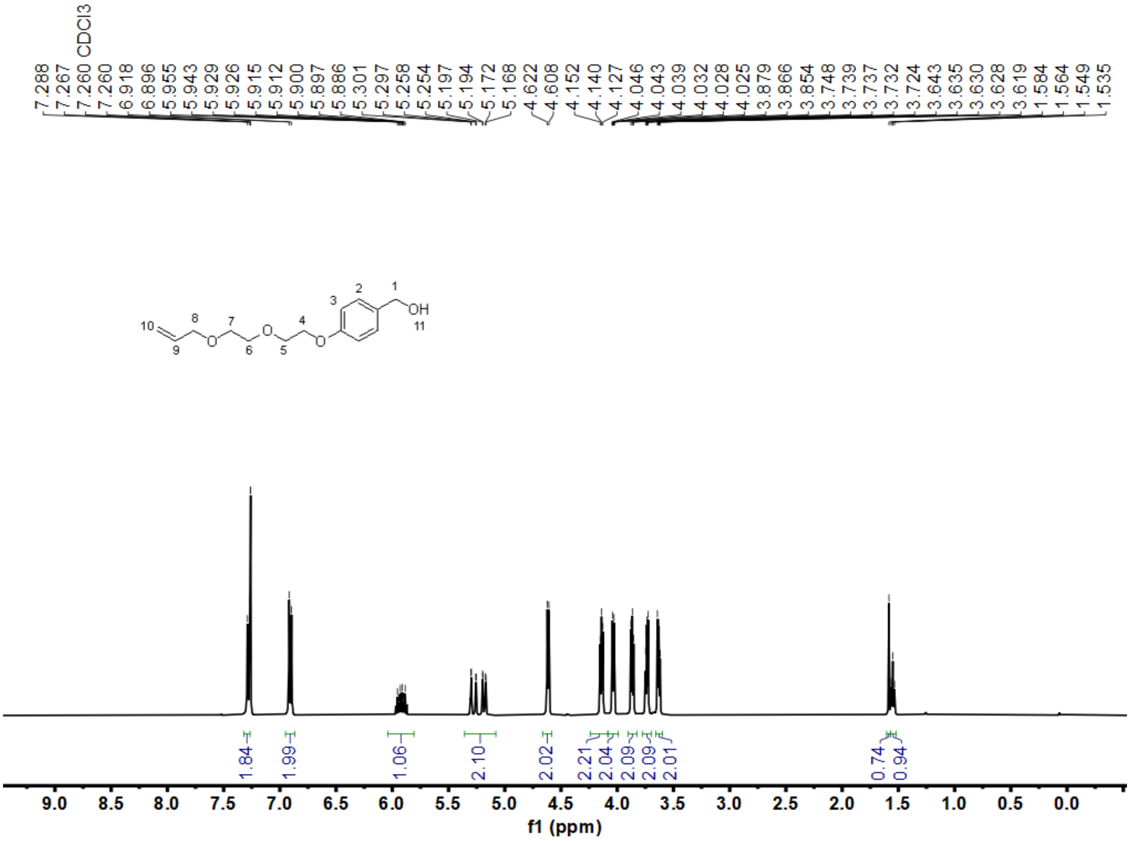

Spectrum S16. ^{1}H NMR spectrum (500 MHz, $CDCl_3$, 298 K) of **S3**.

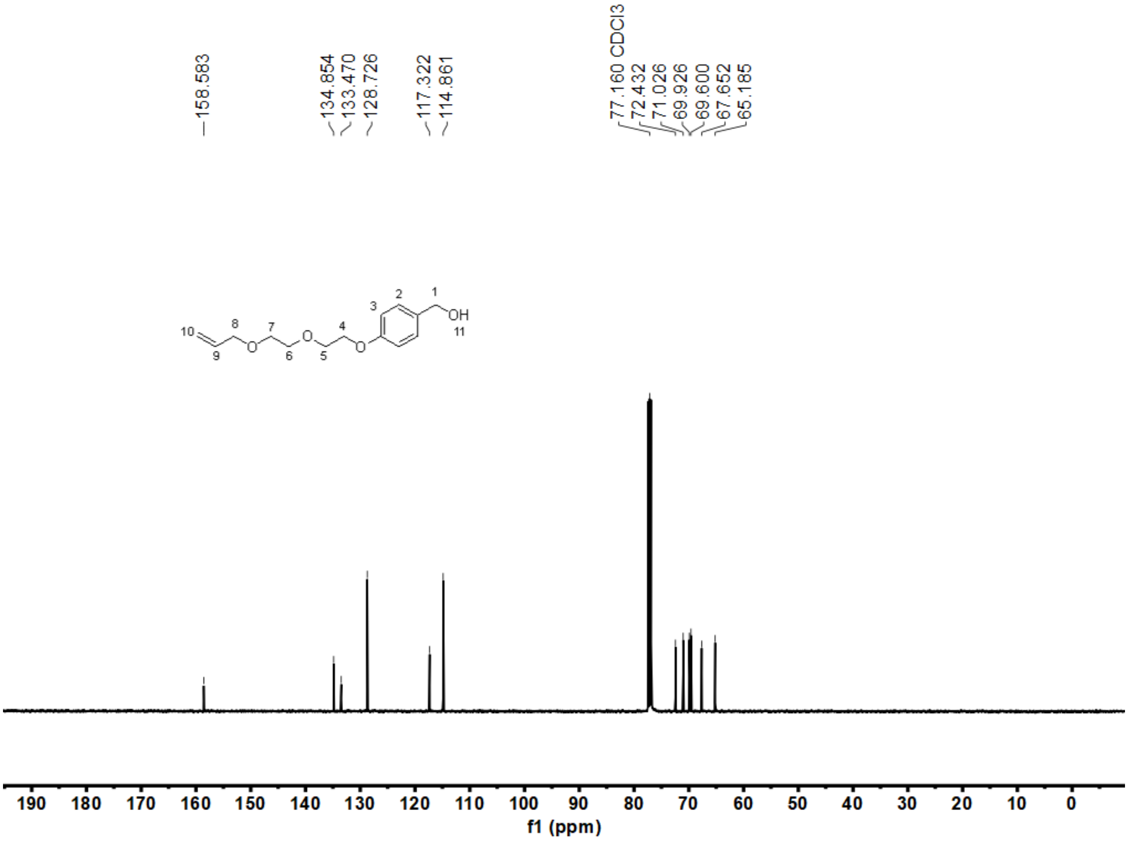

Spectrum S17. ^{13}C NMR spectrum (126 MHz, $CDCl_3$, 298 K) of **S3**.

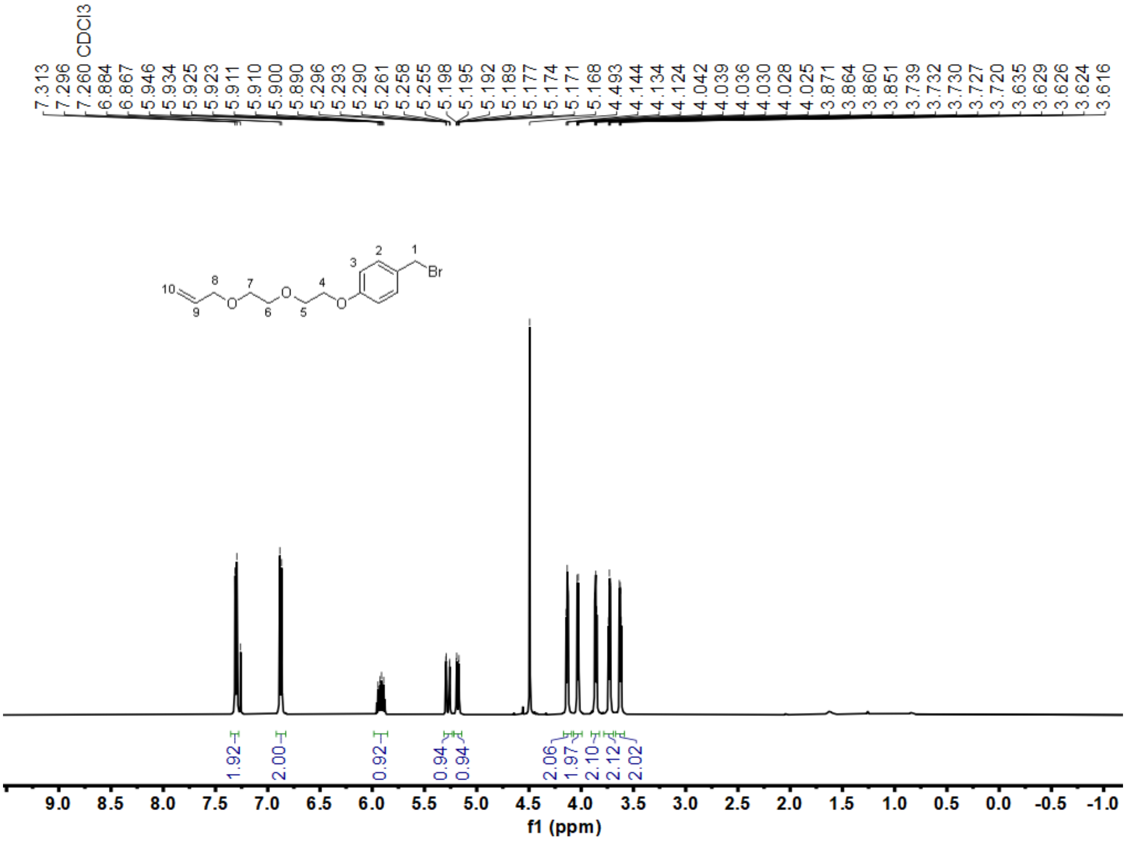

Spectrum S18. ^{1}H NMR spectrum (500 MHz, $CDCl_3$, 298 K) of **S4**.

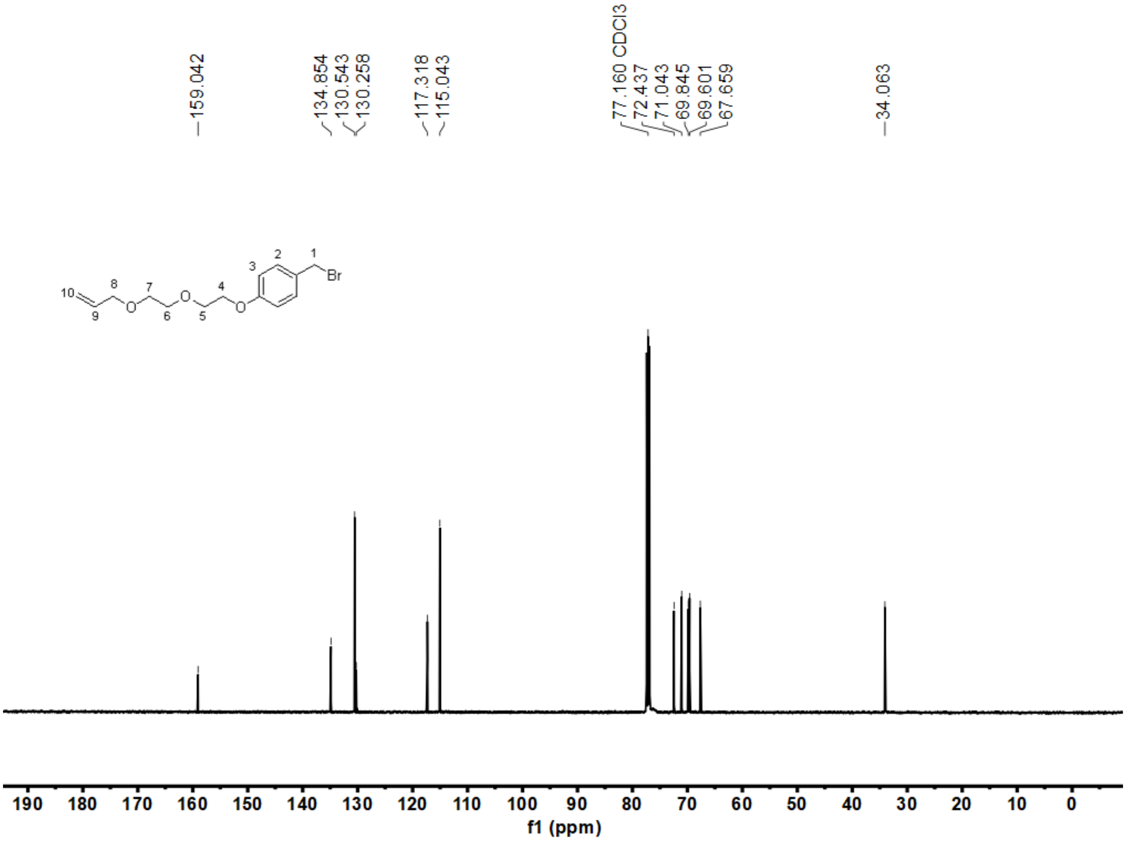

Spectrum S19. ^{13}C NMR spectrum (126 MHz, $CDCl_3$, 298 K) of **S4**.

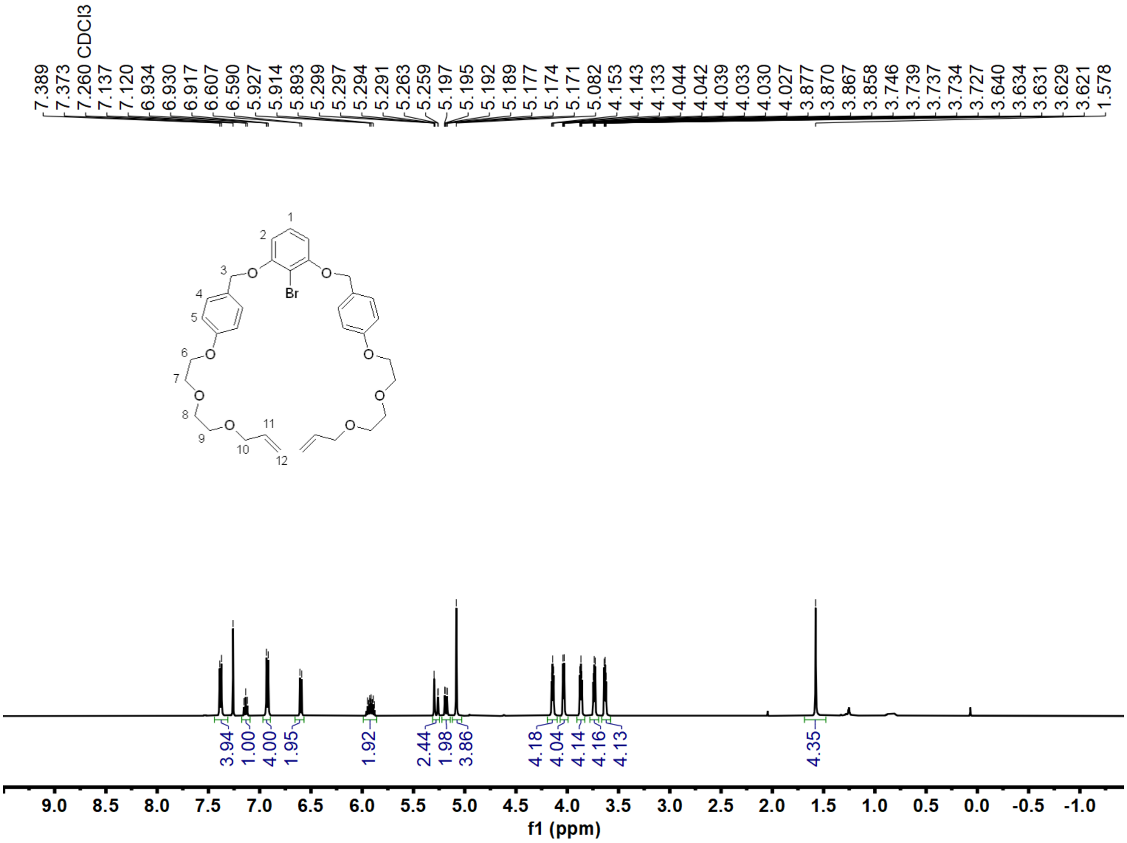

Spectrum S20. ^{1}H NMR spectrum (500 MHz, $CDCl_3$, 298 K) of **S5**.

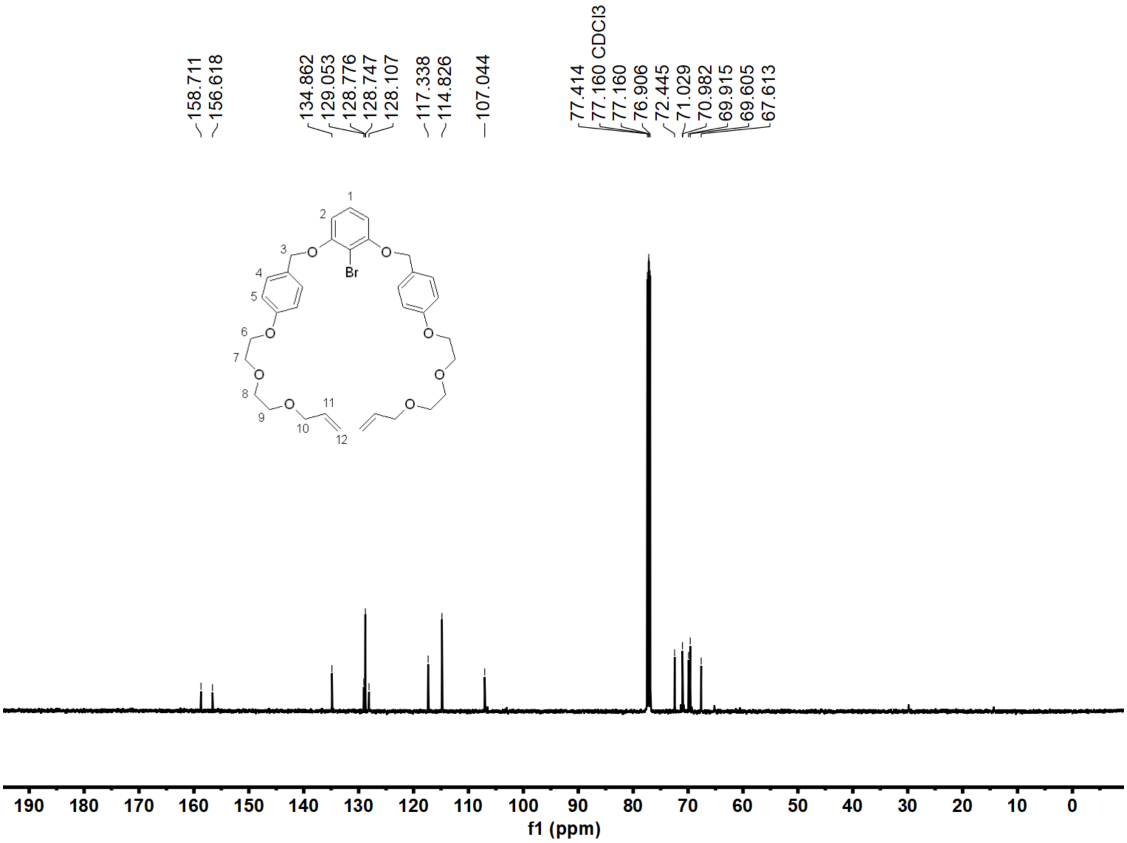

Spectrum S21. ^{13}C NMR spectrum (126 MHz, $CDCl_3$, 298 K) of **S5**.

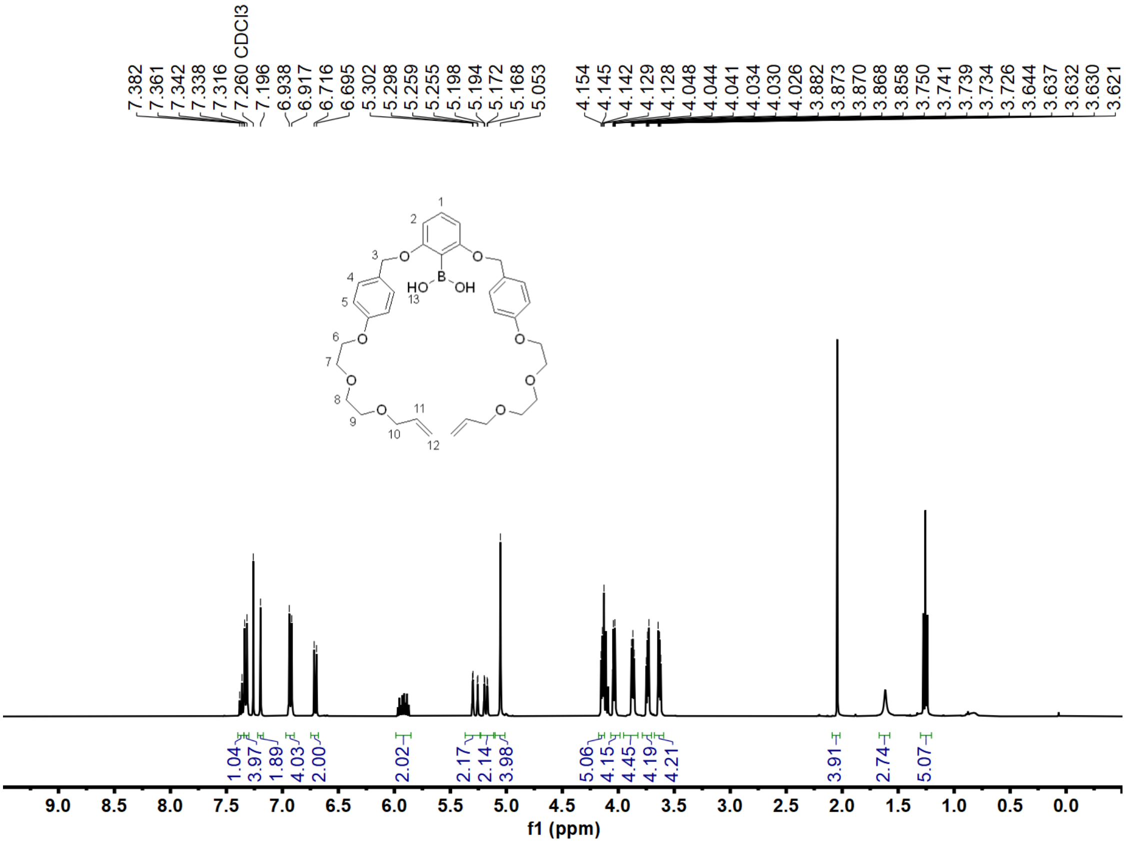

Spectrum S22. ^{1}H NMR spectrum (500 MHz, $CDCl_3$, 298 K) of **1**.

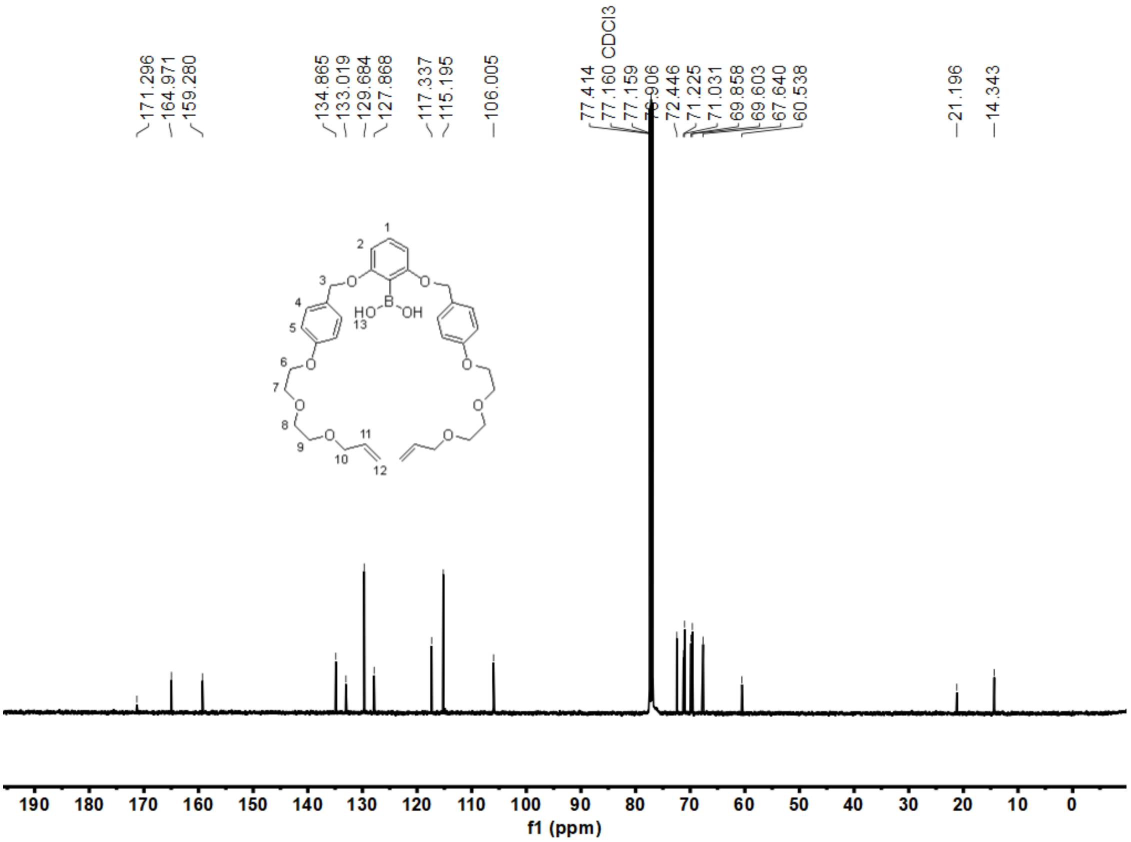

Spectrum S23. ^{13}C NMR spectrum (126 MHz, $CDCl_3$, 298 K) of **1**.

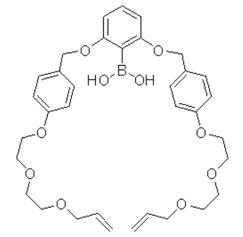

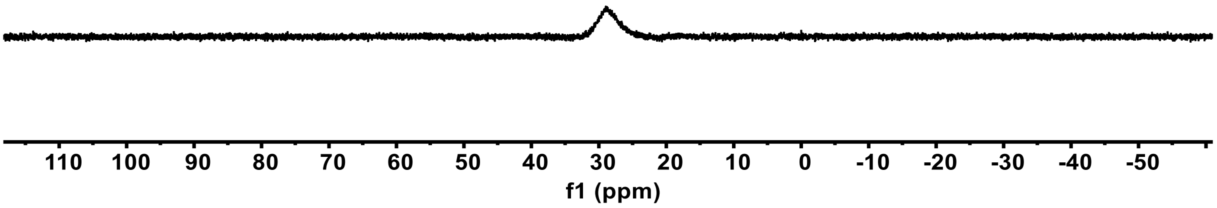

Spectrum S24. ^{11}B NMR spectrum (160 MHz, $CDCl_3$, 298 K) of **1**.

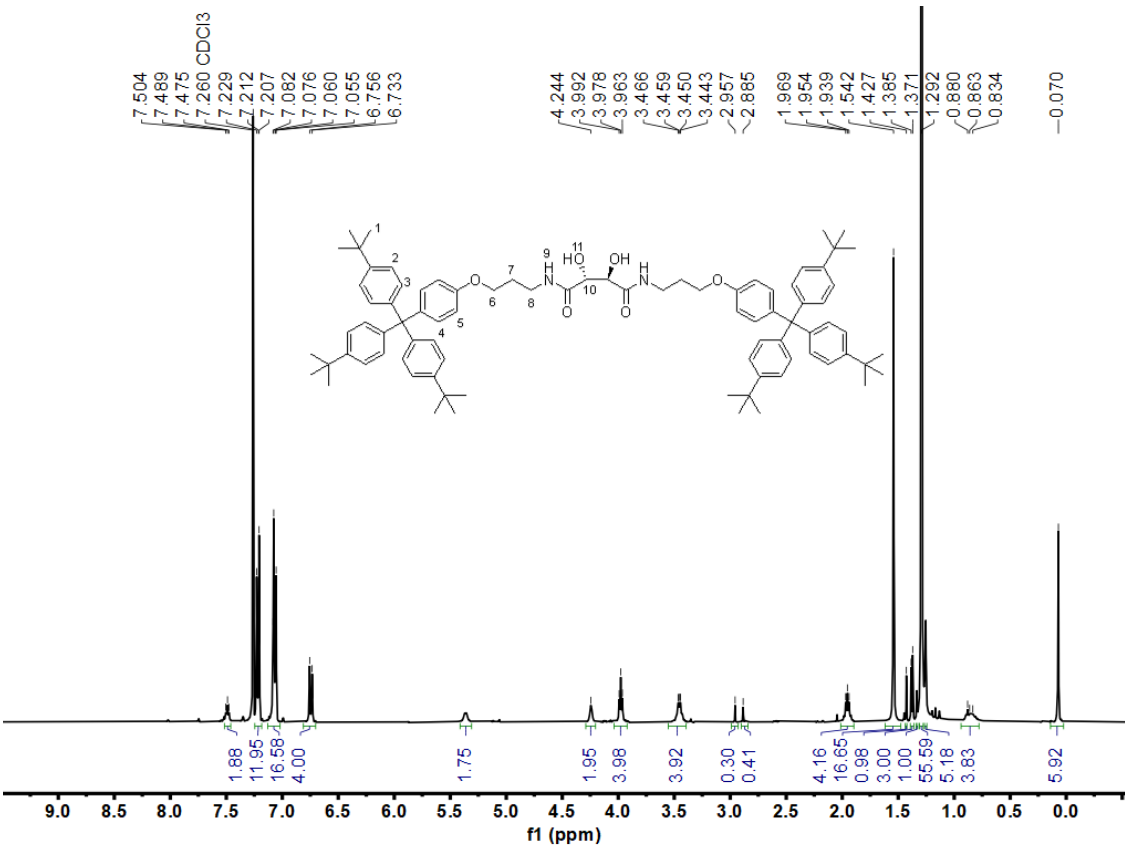

Spectrum S25. ^{1}H NMR spectrum (500 MHz, $CDCl_3$, 298 K) of **2**.

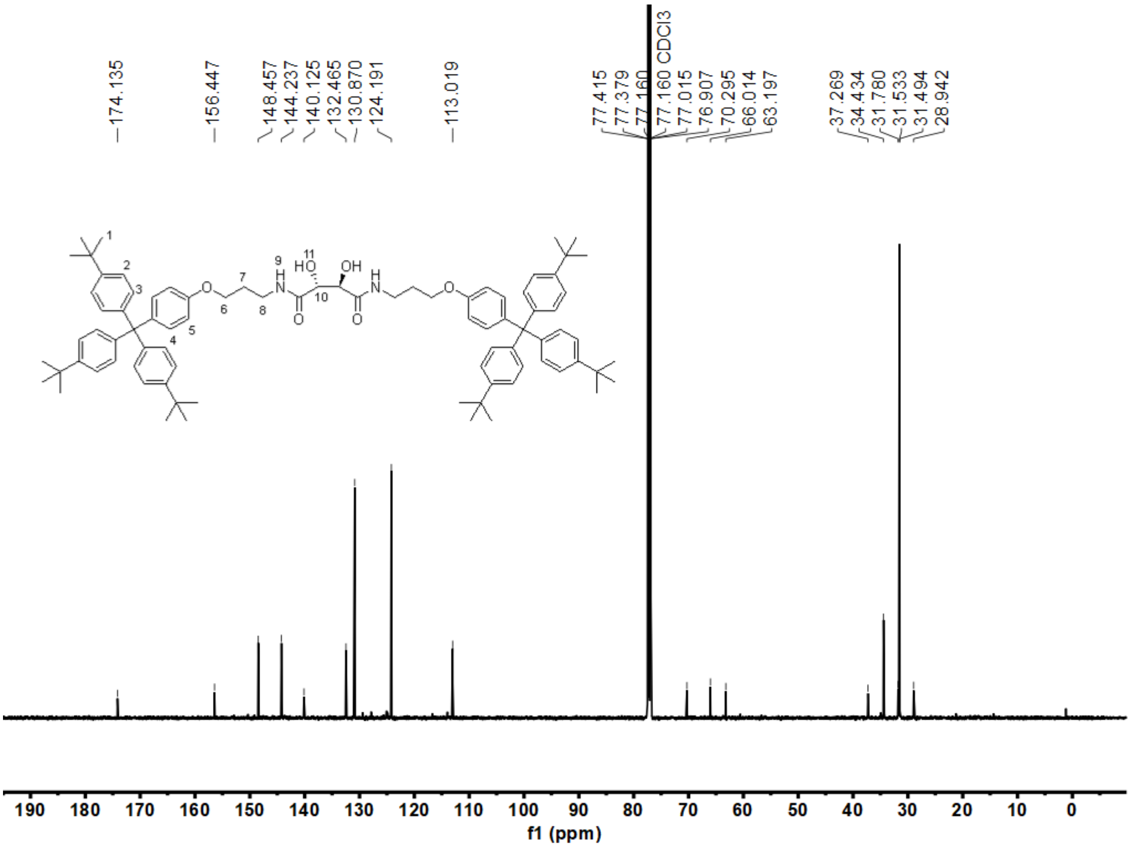

Spectrum S26. ^{13}C NMR spectrum (126 MHz, $CDCl_3$, 298 K) of **2**.

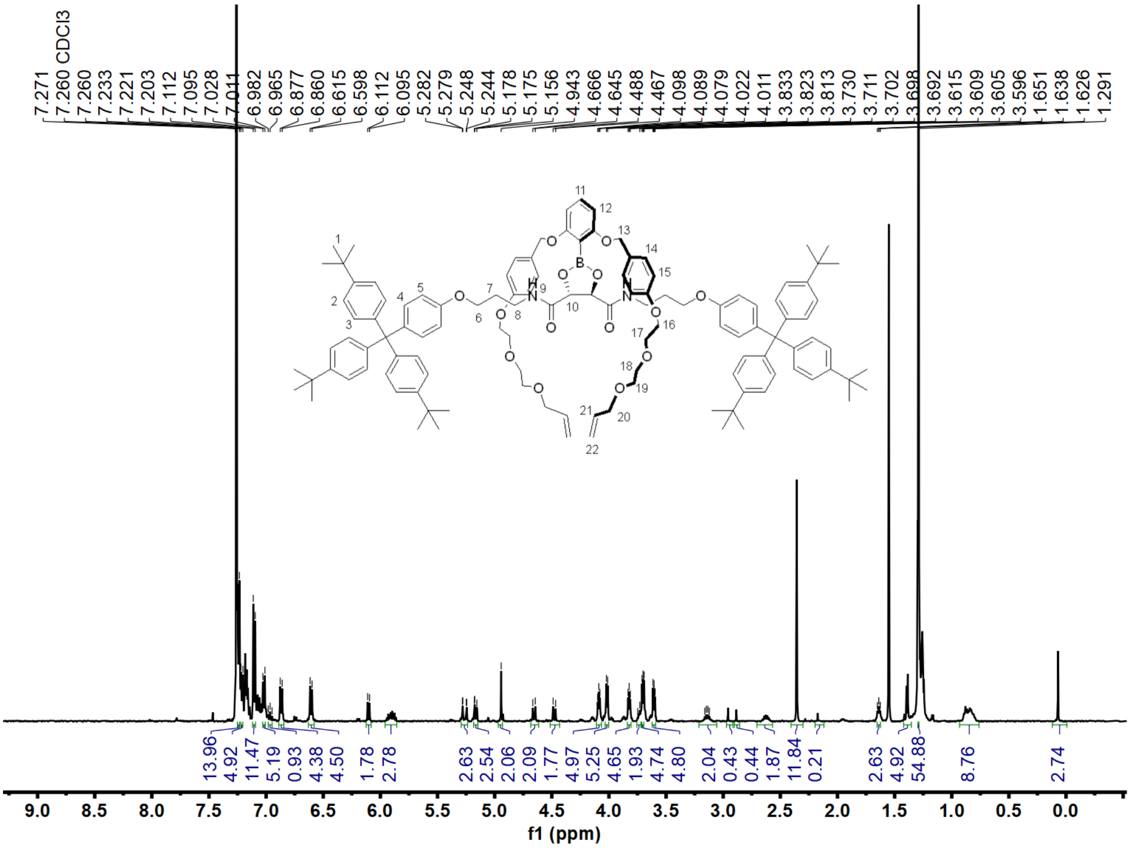

Spectrum S27. ^{1}H NMR spectrum (500 MHz, $CDCl_3$, 298 K) of **3**.

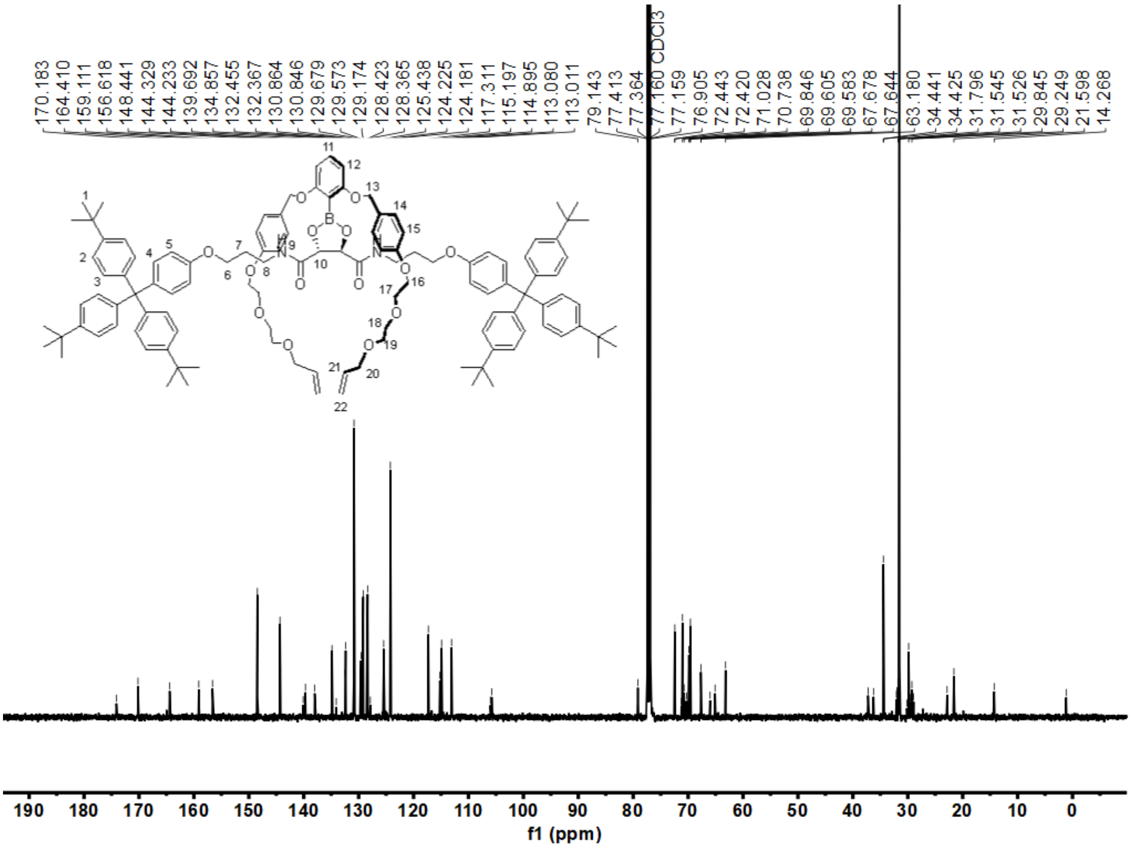

Spectrum S28. ^{13}C NMR spectrum (126 MHz, $CDCl_3$, 298 K) of **3**.

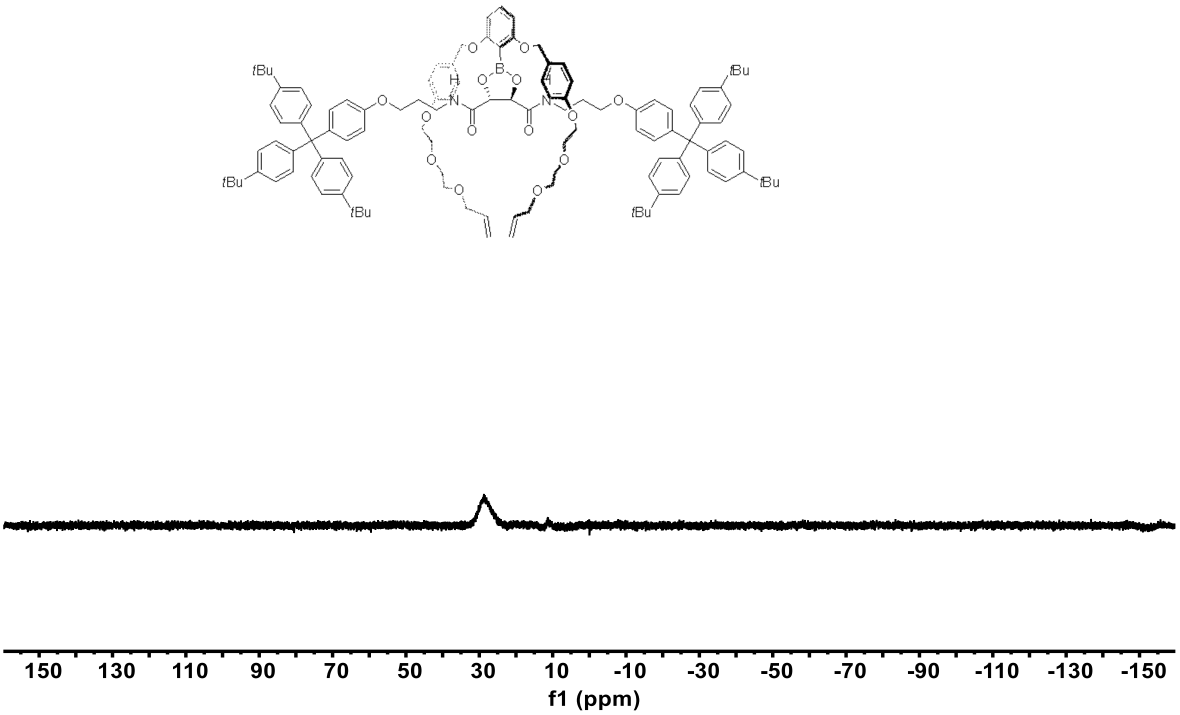

Spectrum S29. ^{11}B NMR spectrum (160 MHz, $CDCl_3$, 298 K) of **3**.

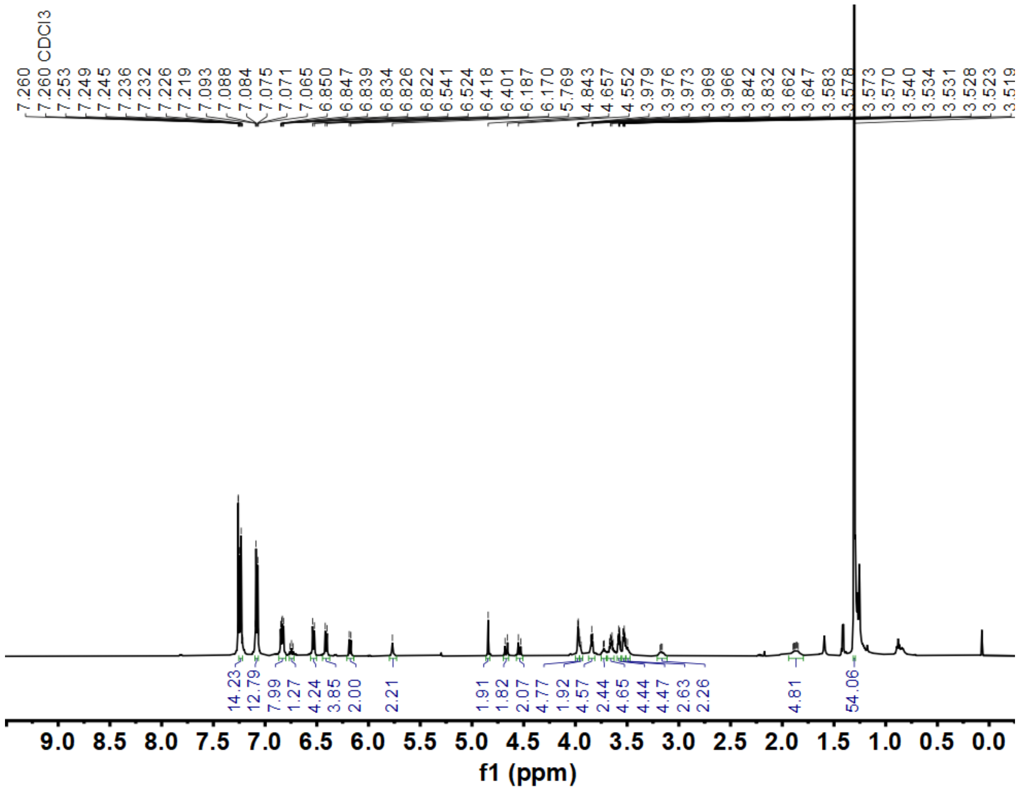

Spectrum S30. ^{1}H NMR spectrum (500 MHz, $CDCl_3$, 298 K) of **4**.

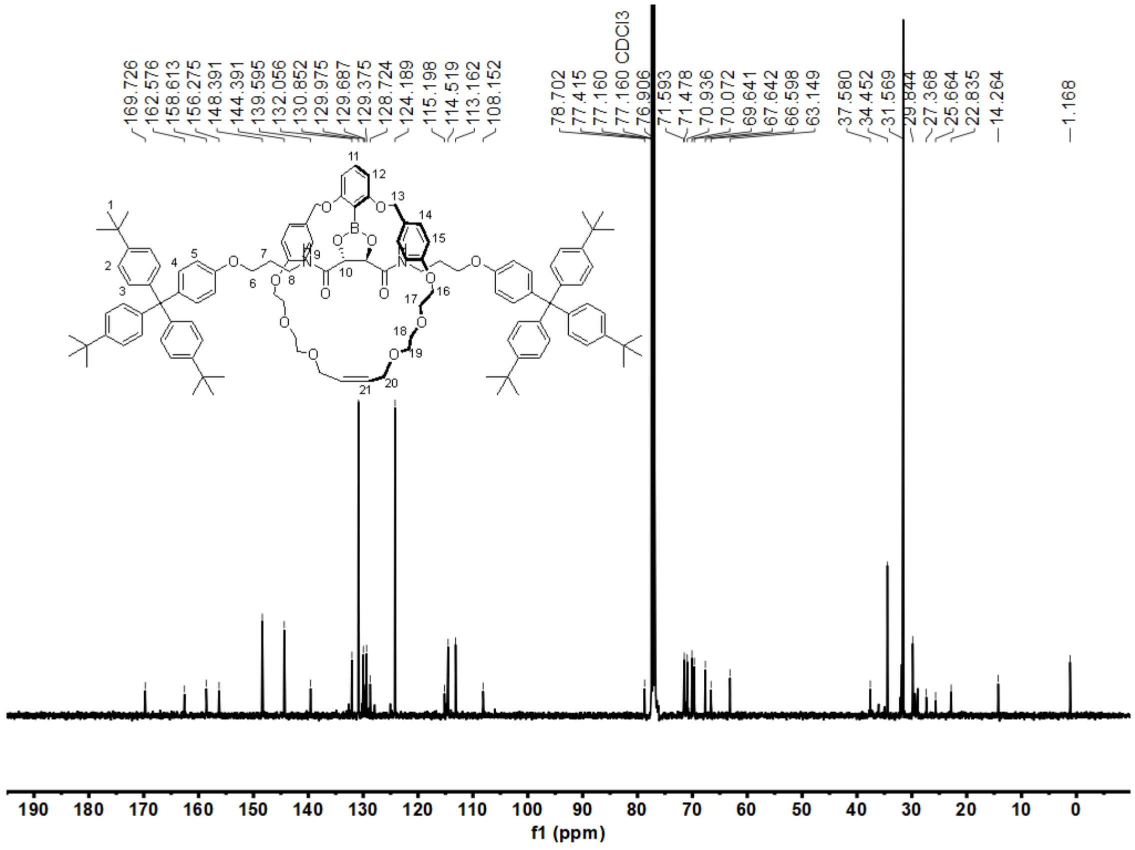

Spectrum S31. ^{13}C NMR spectrum (126 MHz, $CDCl_3$, 298 K) of **4**.

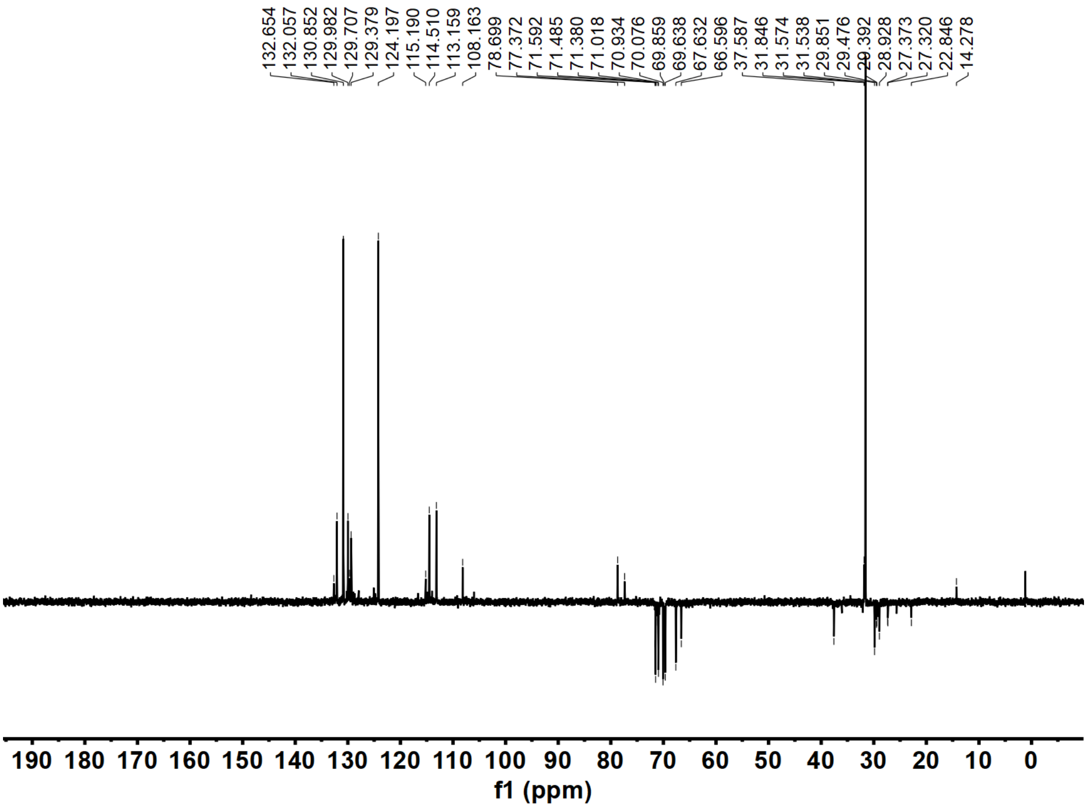

Spectrum S32. DEPT135 spectrum (126 MHz, $CDCl_3$, 298 K) of **4**

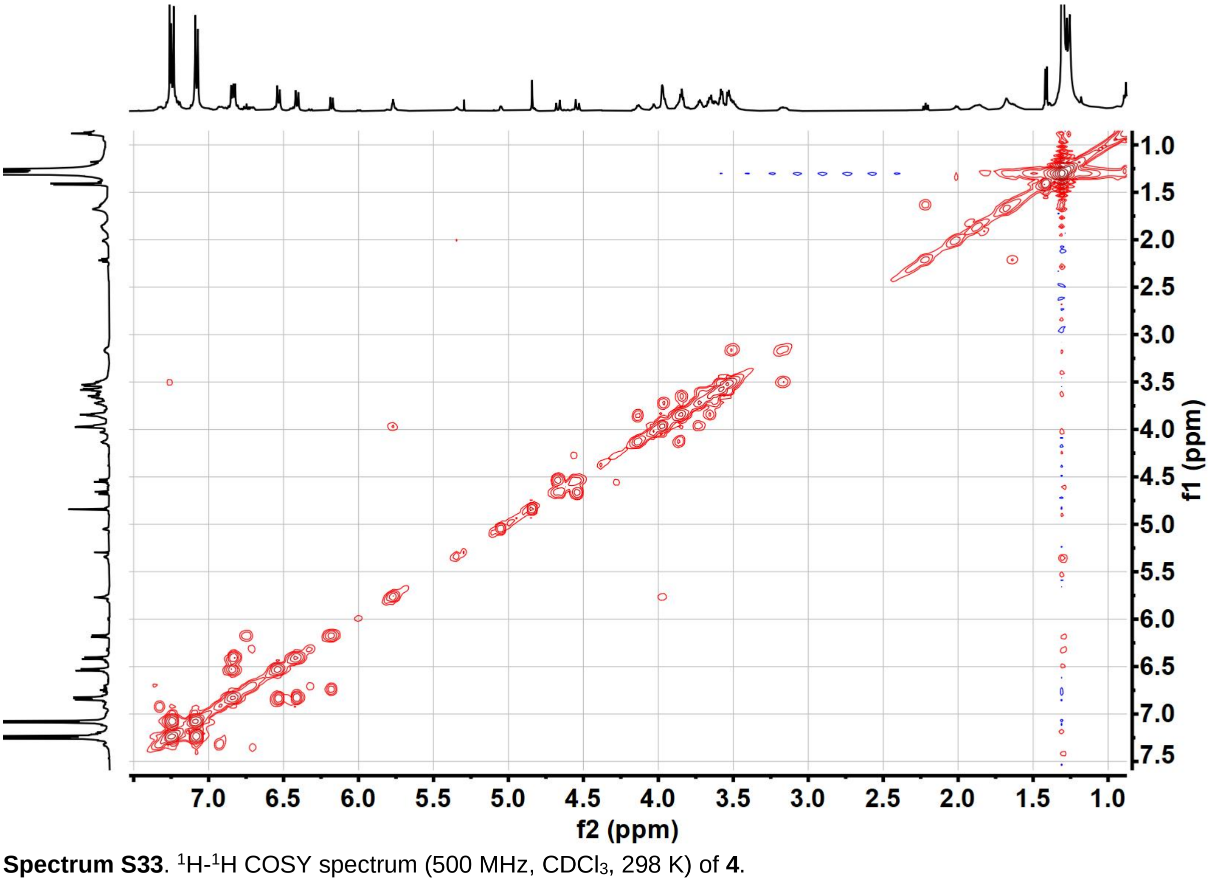

Spectrum S33. ^1H-^1H COSY spectrum (500 MHz, $CDCl_3$, 298 K) of **4**.

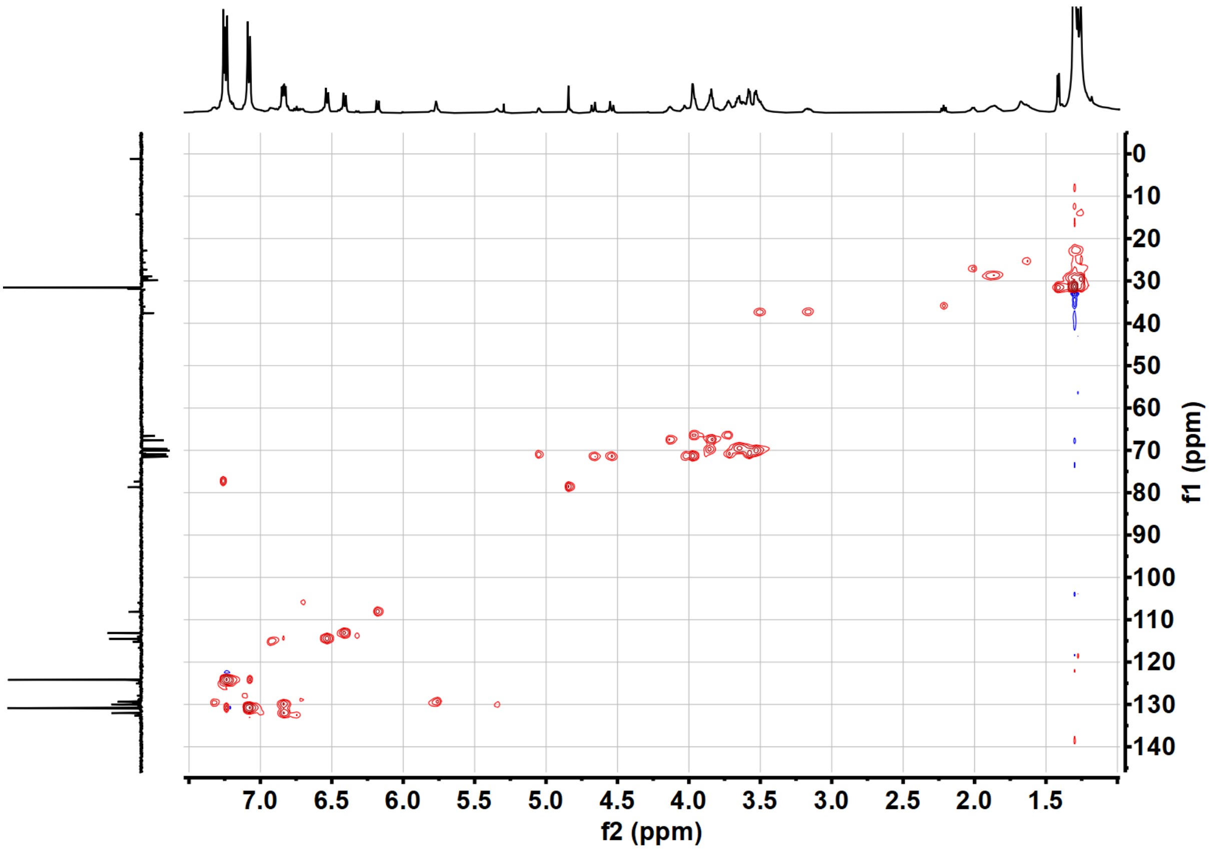

Spectrum S34. ^{1}H-^{13}C HSQC spectrum (500 MHz, $CDCl_3$, 298 K) of **4**.

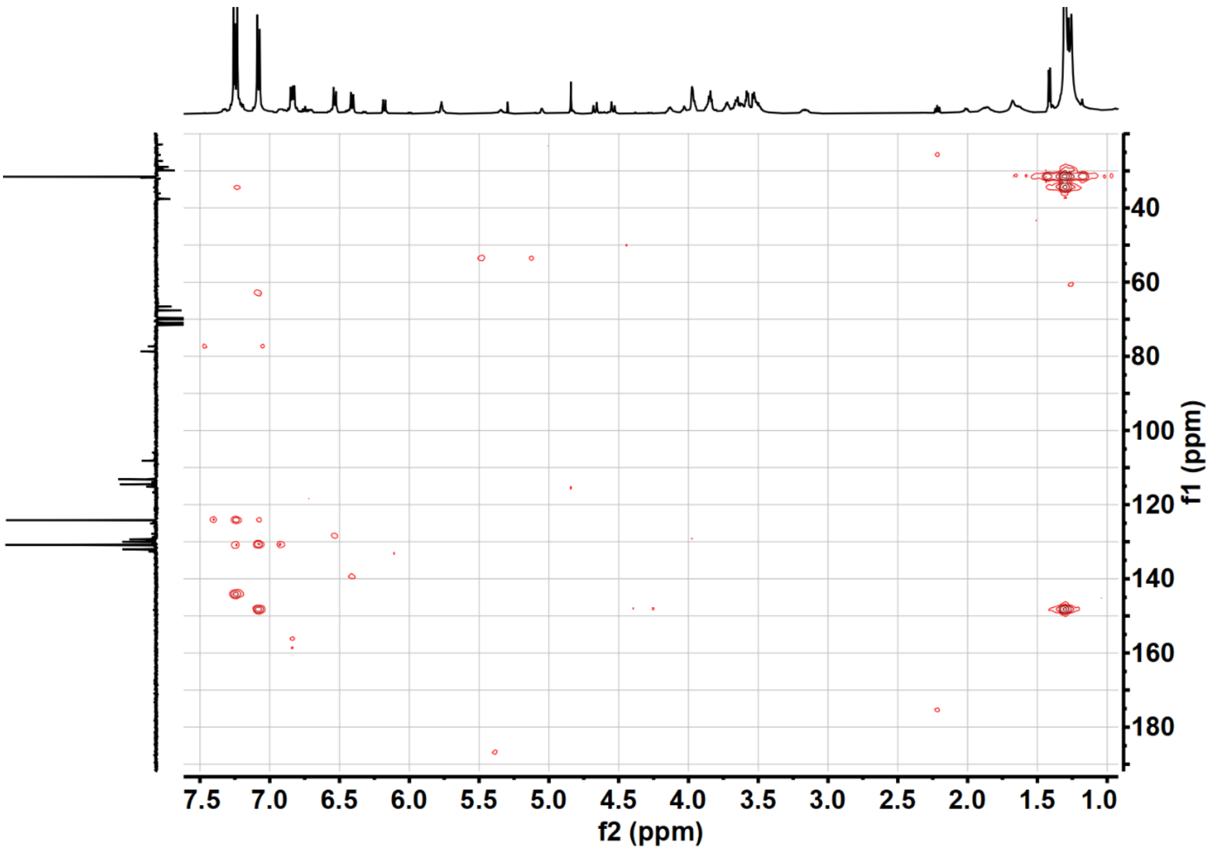

Spectrum S35. ^{1}H-^{13}C HMBC spectrum (500 MHz, $CDCl_3$, 298 K) of **4**.

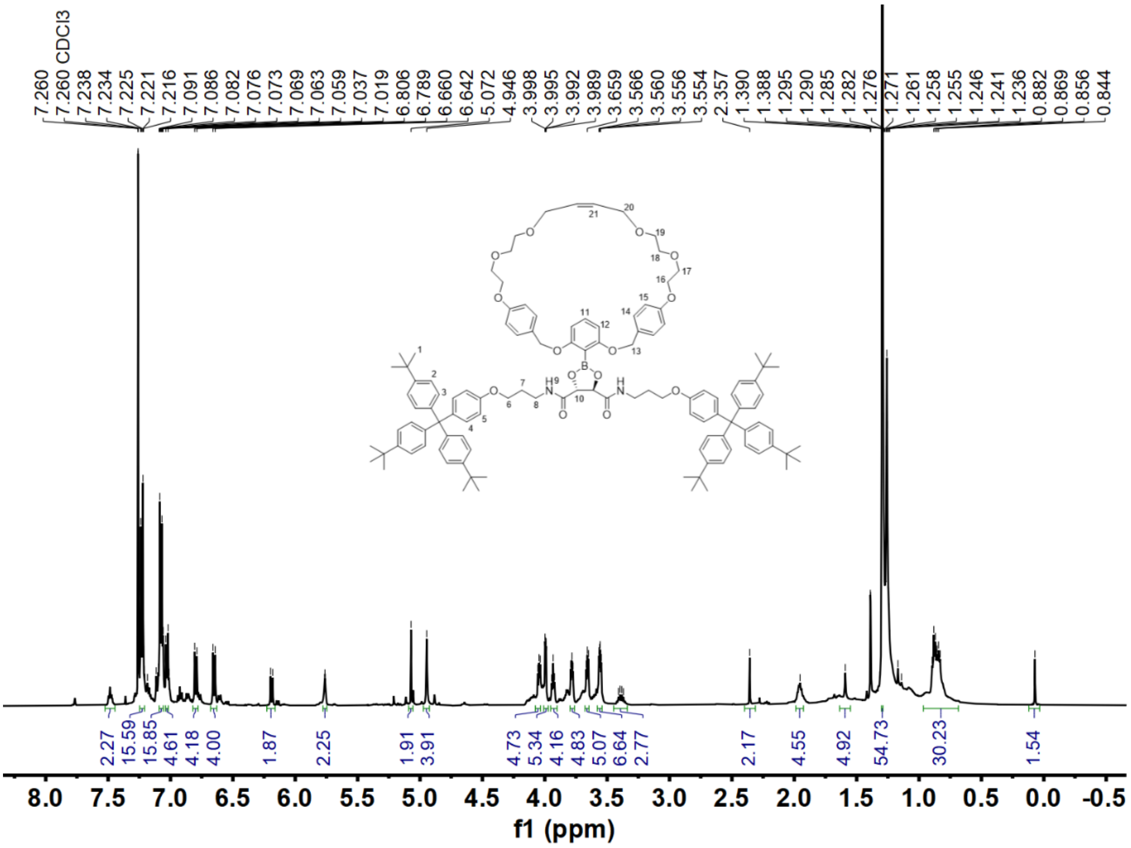

Spectrum S36. ^{1}H NMR spectrum (500 MHz, $CDCl_3$, 298 K) of **4'**.

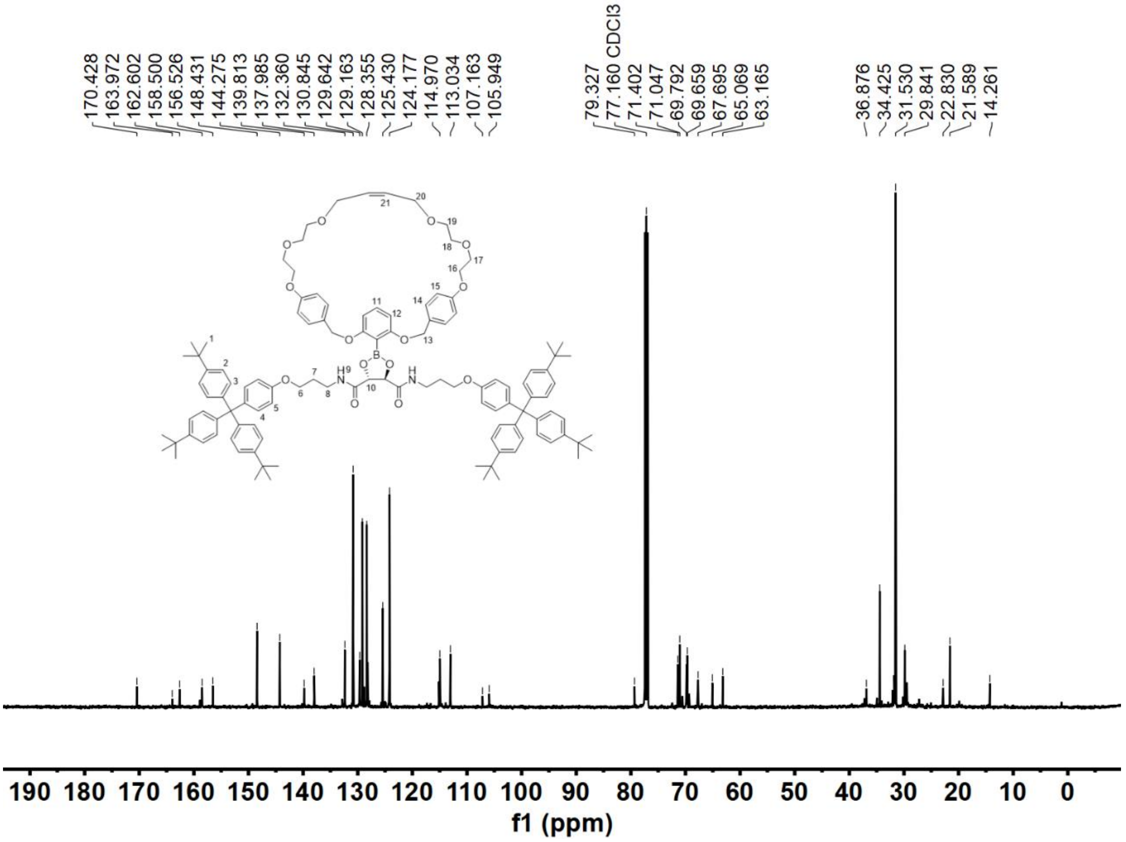

Spectrum S37. ^{13}C NMR spectrum (126 MHz, $CDCl_3$, 298 K) of **4'**.

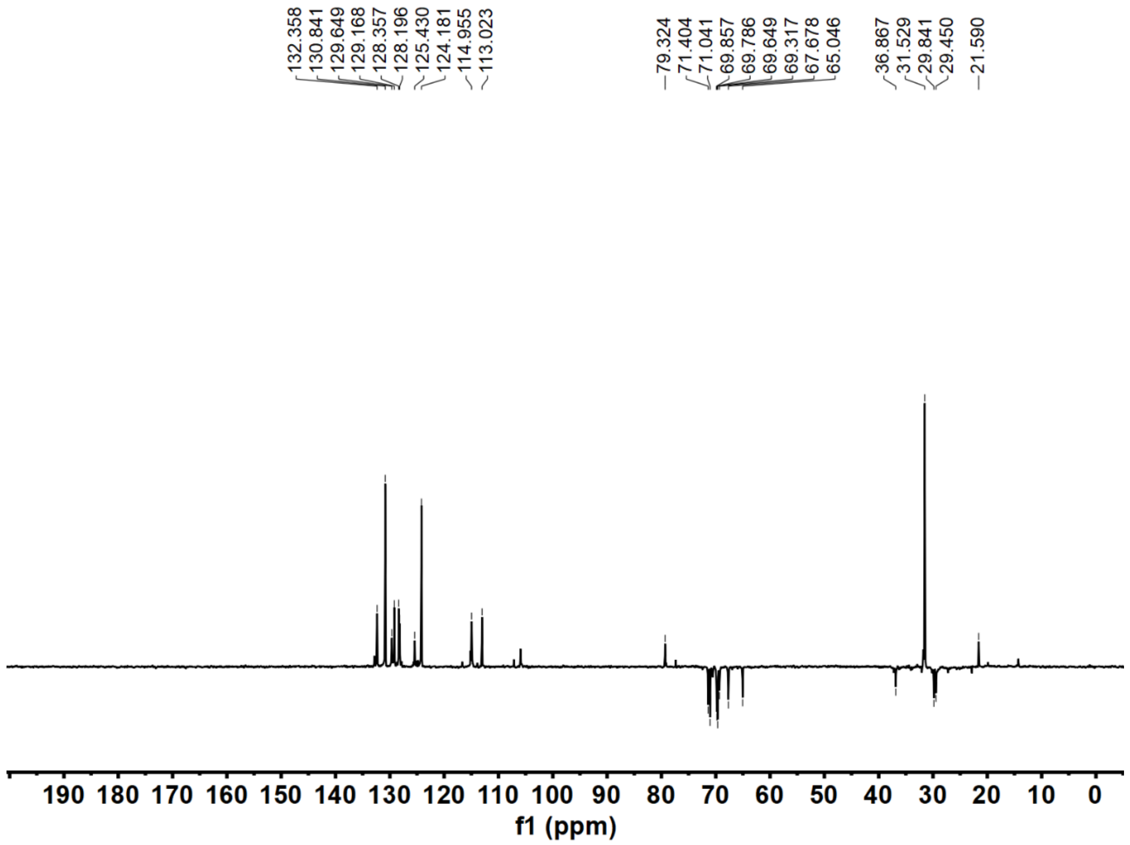

Spectrum S38. DEPT135 spectrum (126 MHz, $CDCl_3$, 298 K) of **4'**

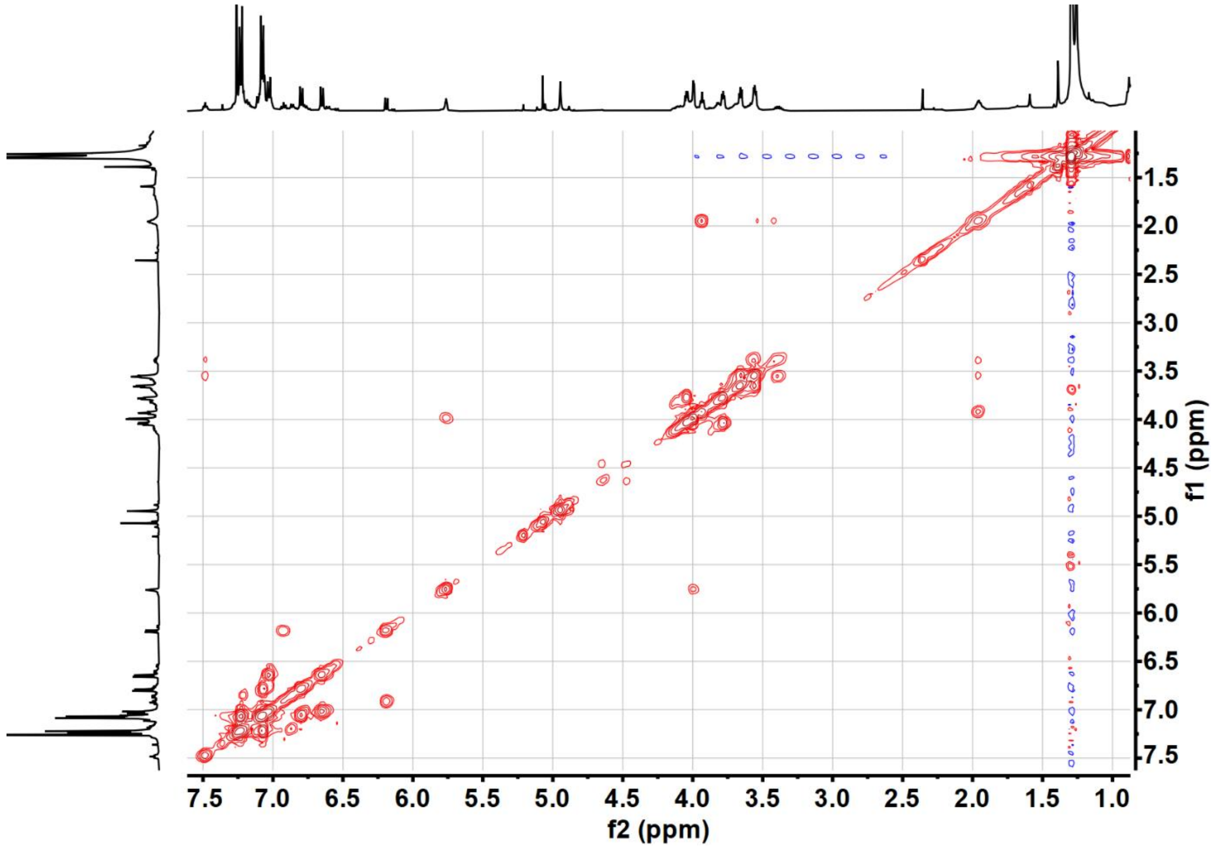

Spectrum S39. ^1H-^1H COSY spectrum (500 MHz, $CDCl_3$, 298 K) of **4'**.

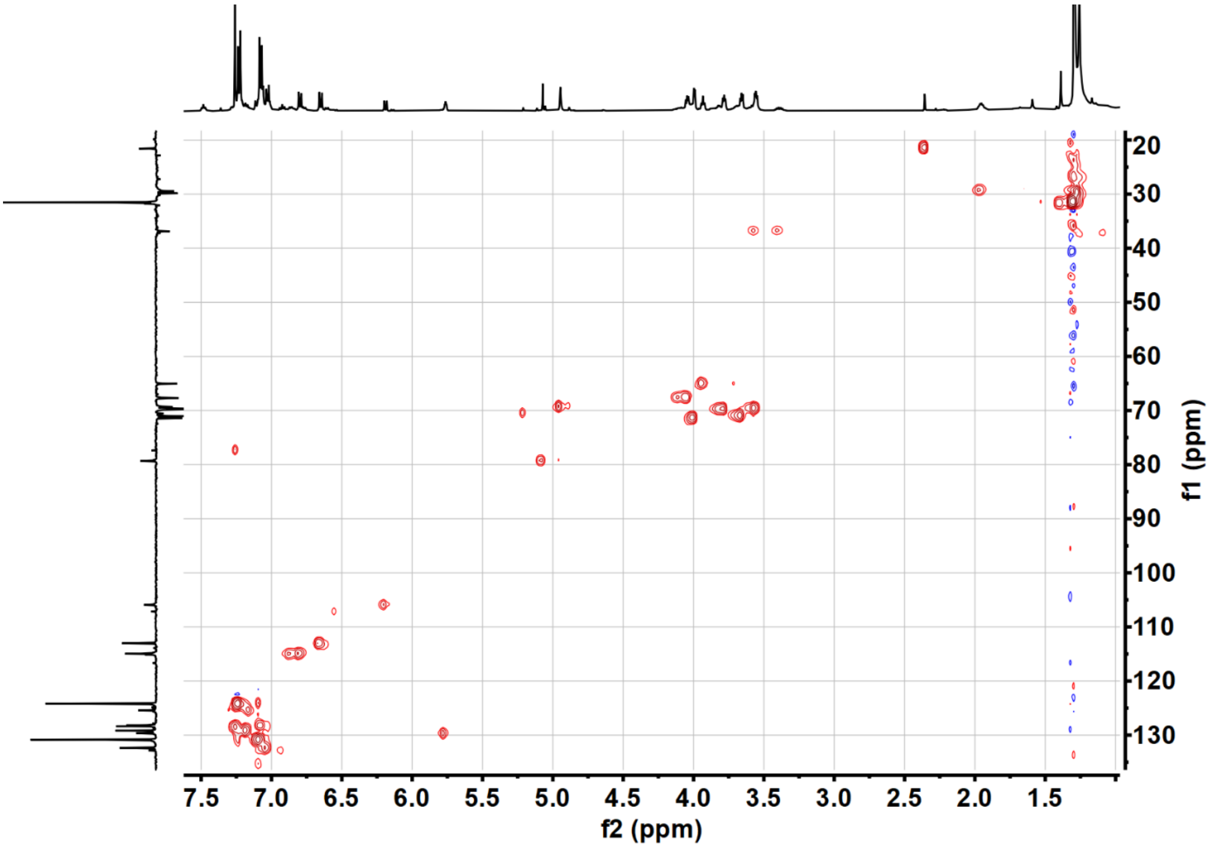

Spectrum S40. ^{1}H-^{13}C HSQC spectrum (500 MHz, $CDCl_3$, 298 K) of **4'**.

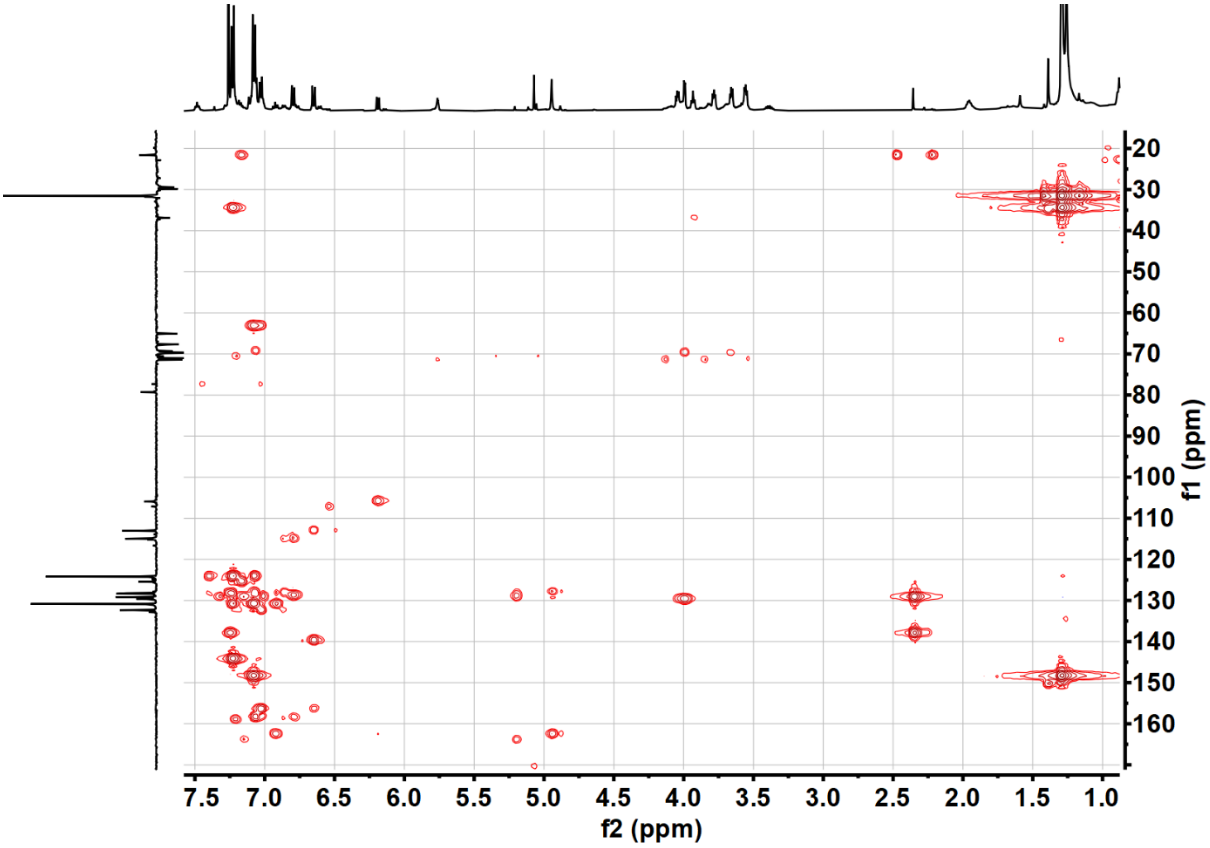

Spectrum S41. ^{1}H-^{13}C HMBC spectrum (500 MHz, $CDCl_3$, 298 K) of **4'**.

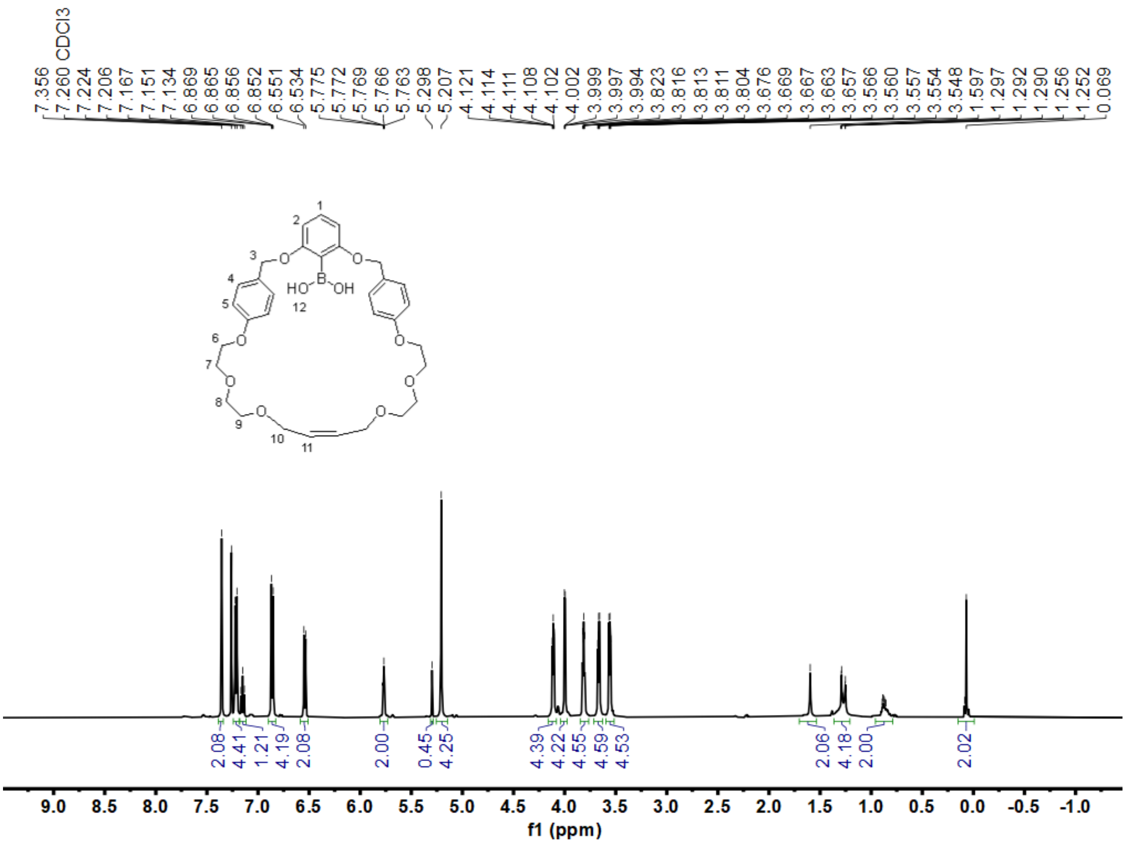

Spectrum S42. ^{1}H NMR spectrum (500 MHz, $CDCl_3$, 298 K) of **6**.

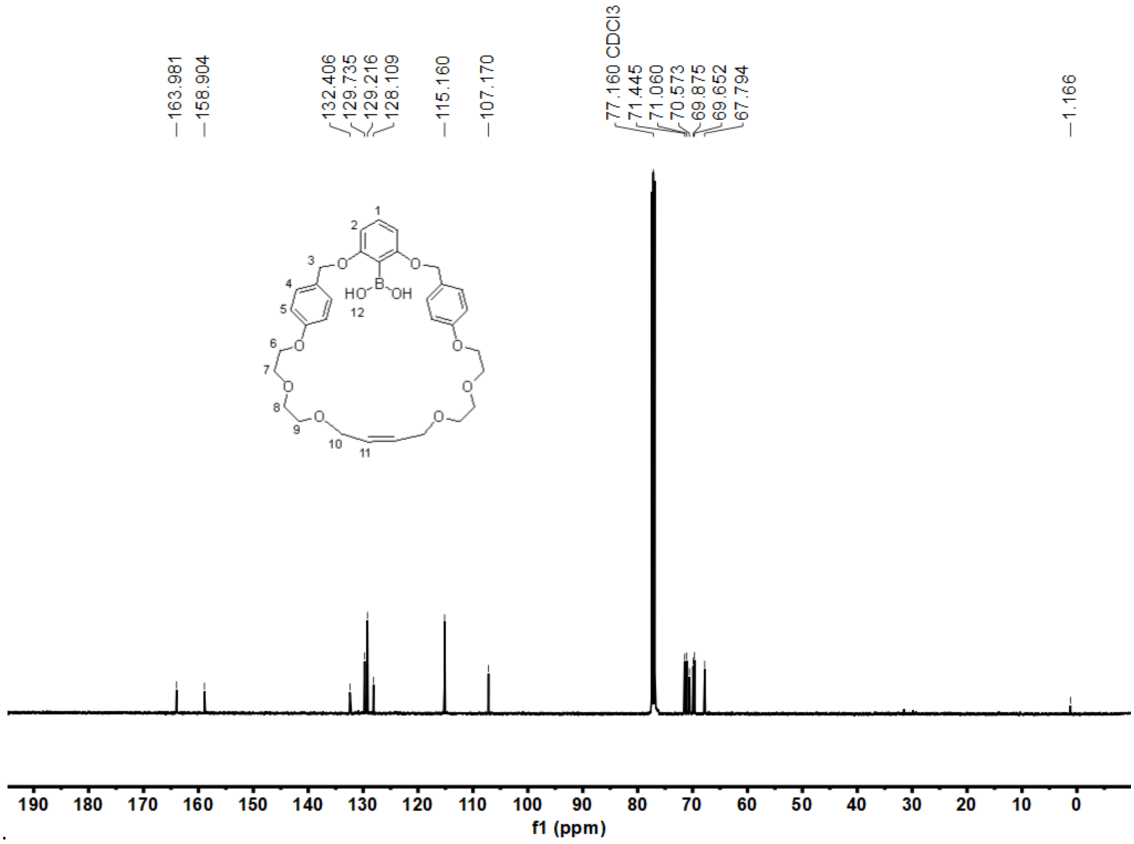

.

Spectrum S43. ^{13}C NMR spectrum (126 MHz, $CDCl_3$, 298 K) of **6**.

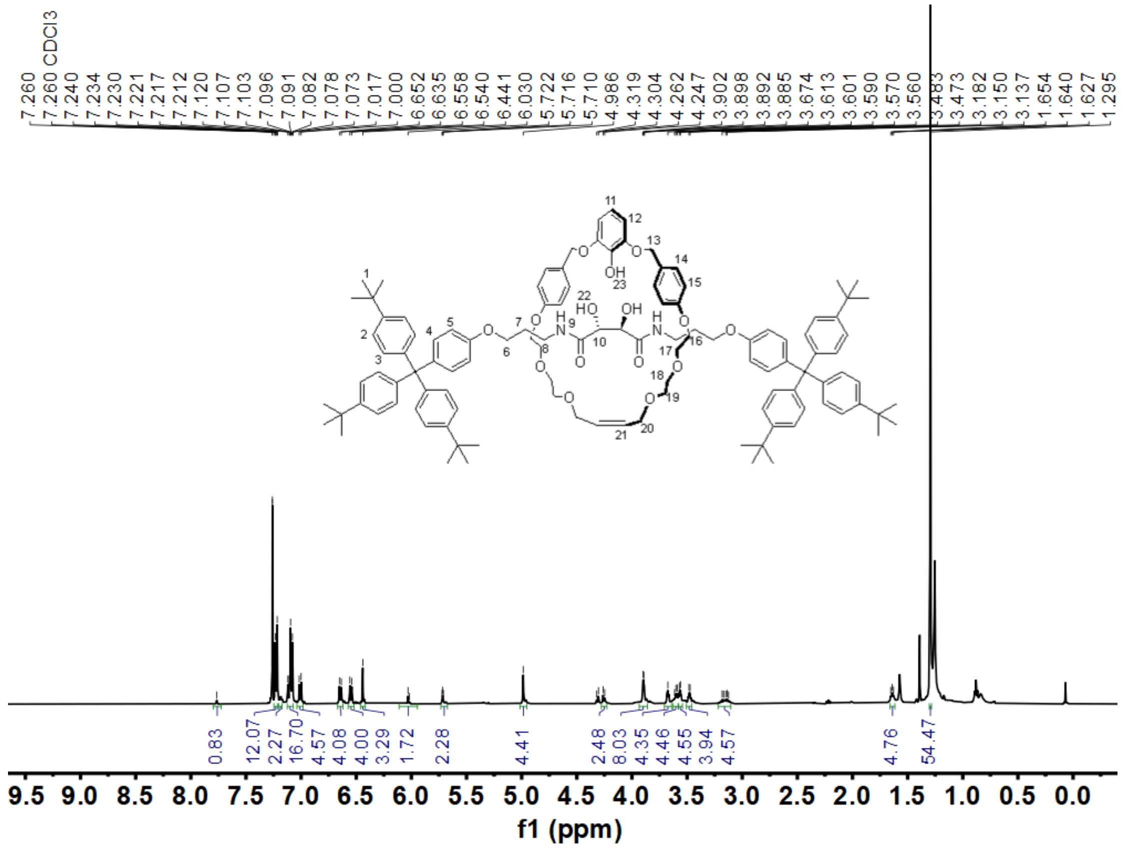

Spectrum S44. ^{1}H NMR spectrum (500 MHz, $CDCl_3$, 298 K) of **7**.

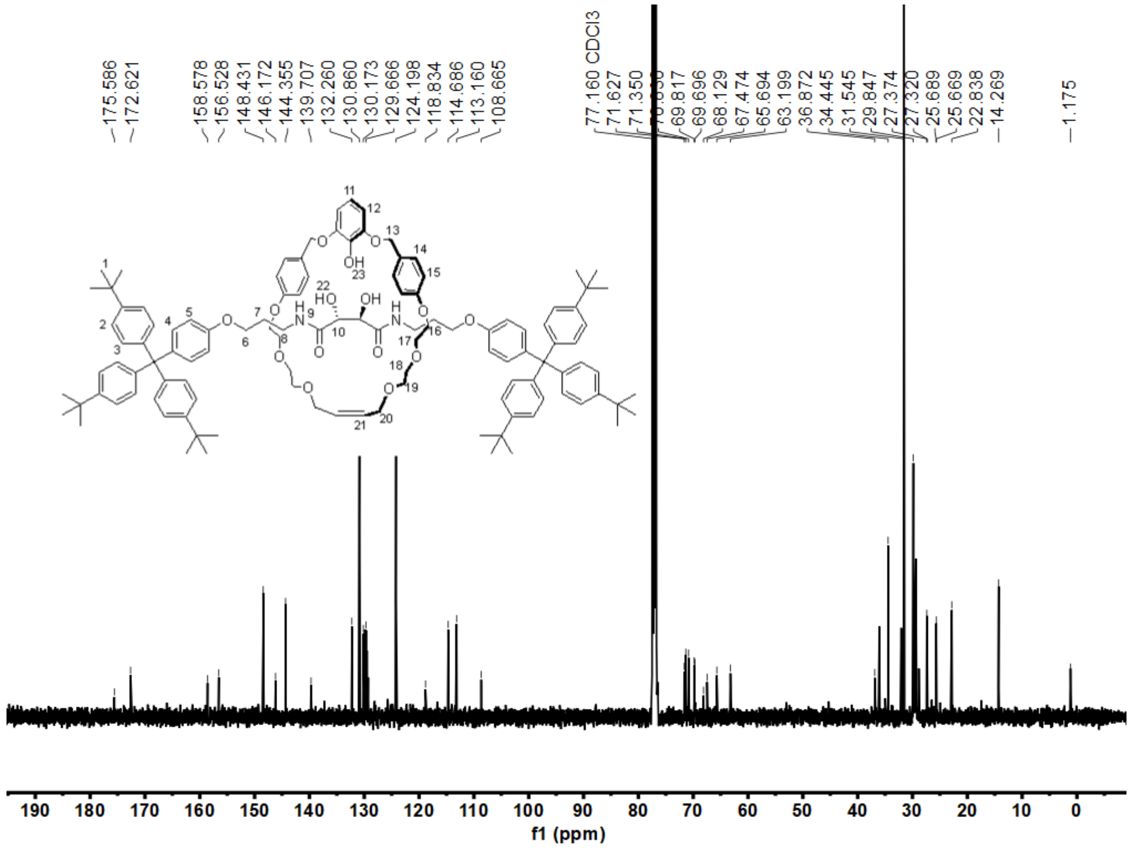

Spectrum S45. ^{13}C NMR spectrum (126 MHz, $CDCl_3$, 298 K) of **7**.

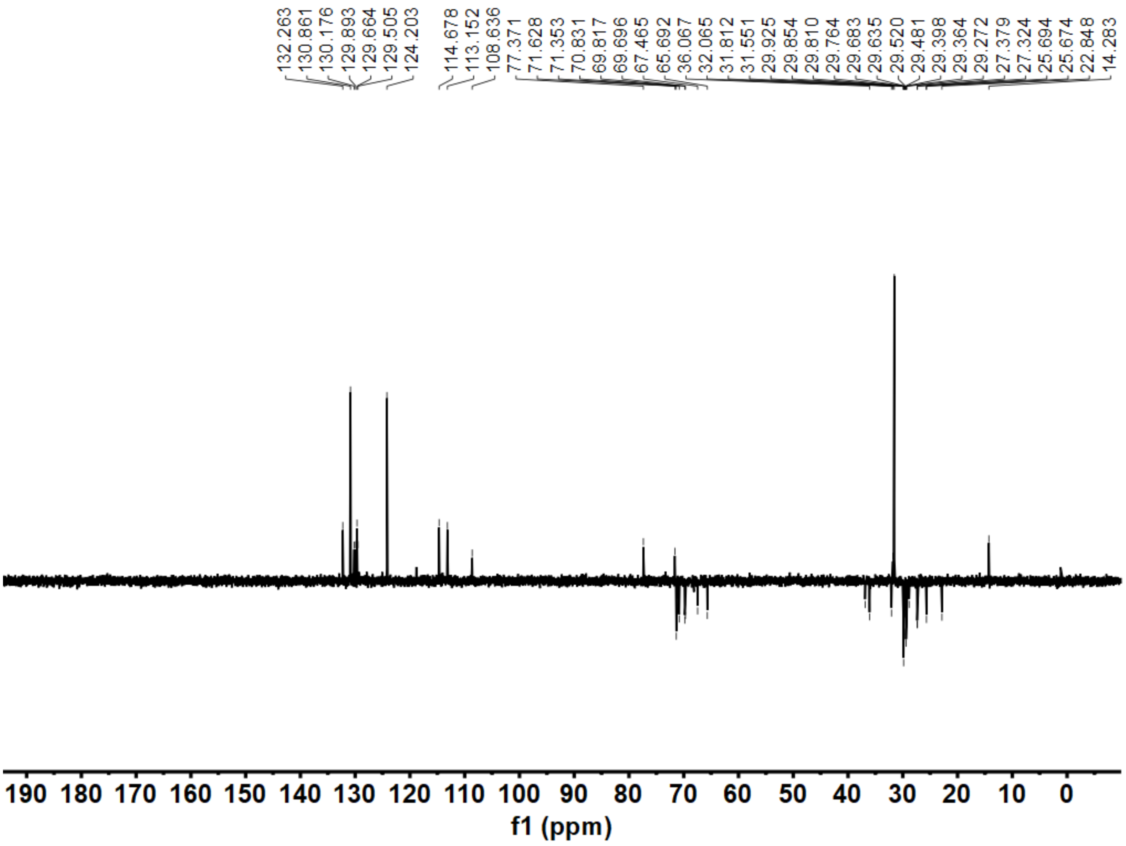

Spectrum S46. DEPT135 spectrum (126 MHz, $CDCl_3$, 298 K) of **7**

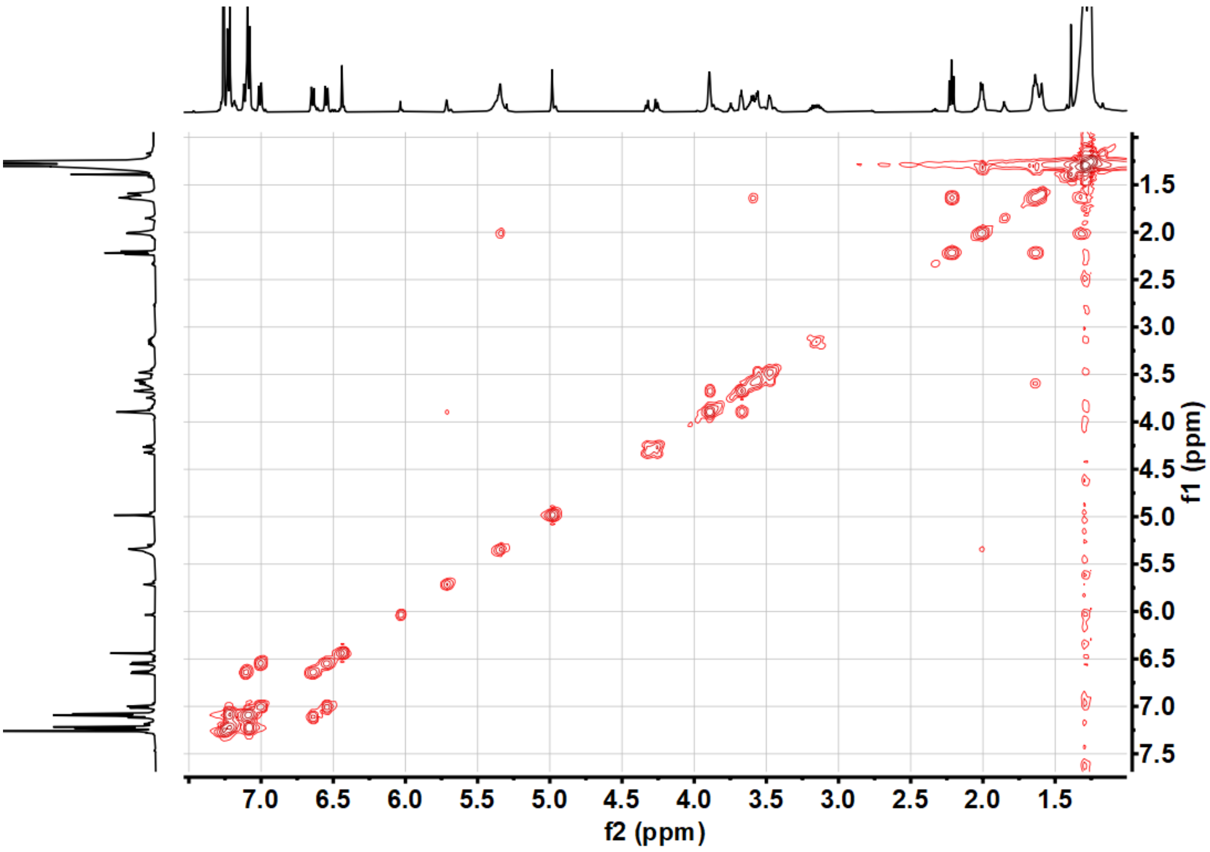

Spectrum S47. ^{1}H-^{1}H COSY spectrum (500 MHz, $CDCl_3$, 298 K) of **7**.

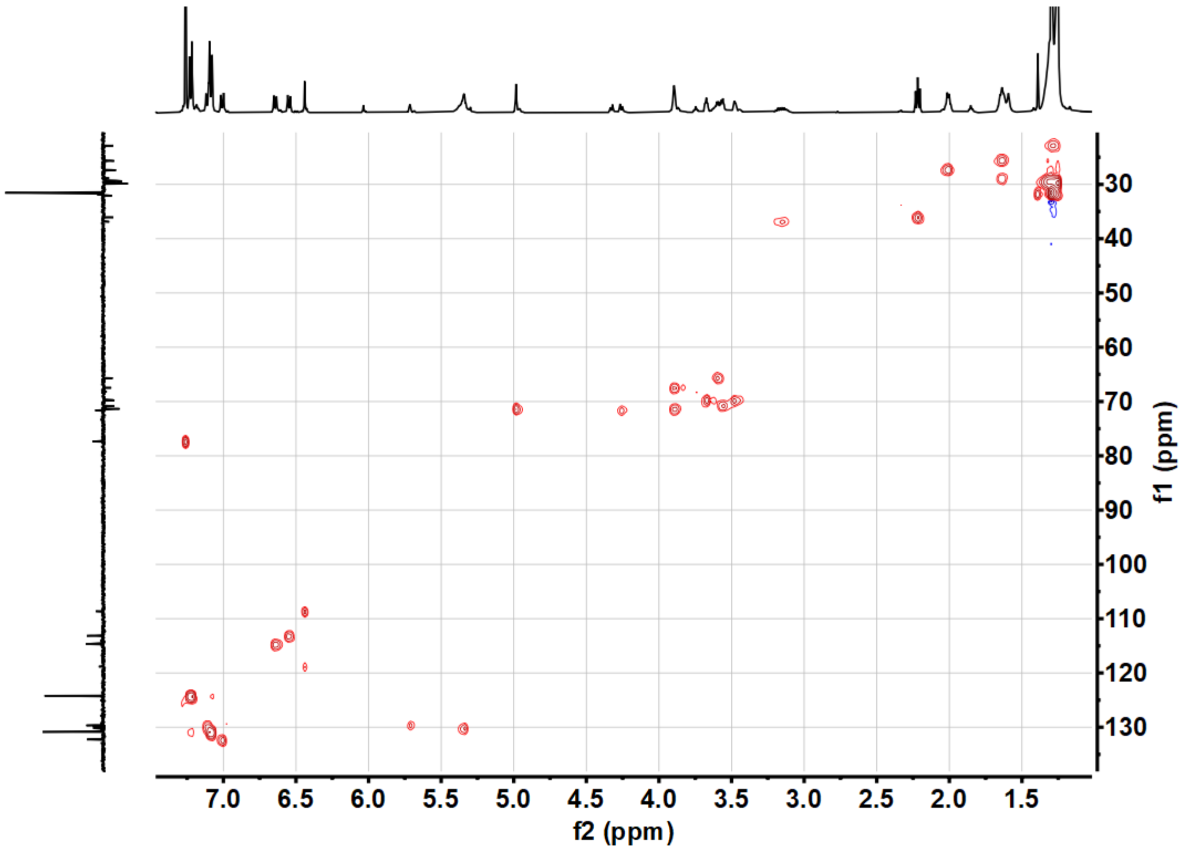

Spectrum S48. ^{1}H-^{13}C HSQC spectrum (500 MHz, $CDCl_3$, 298 K) of **7**.

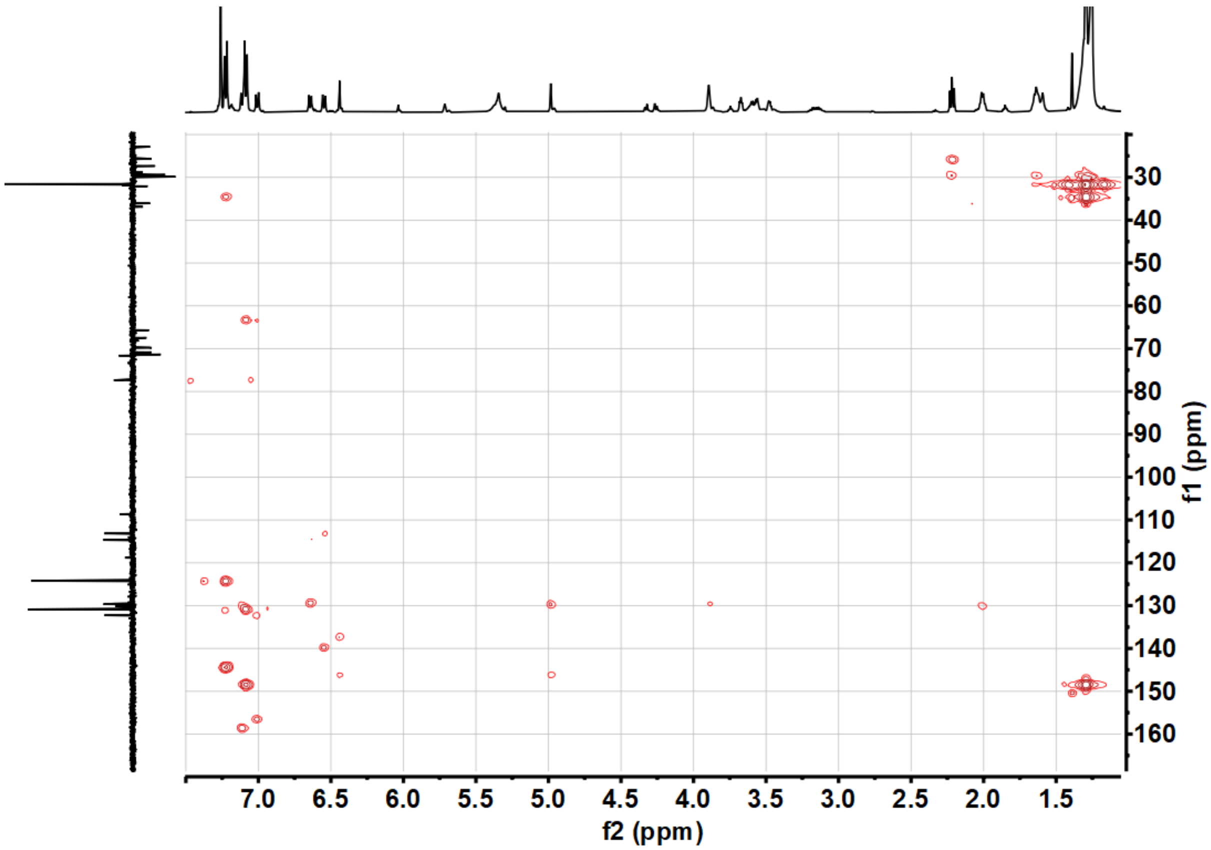

Spectrum S49. ^{1}H-^{13}C HMBC spectrum (500 MHz, $CDCl_3$, 298 K) of **7**.

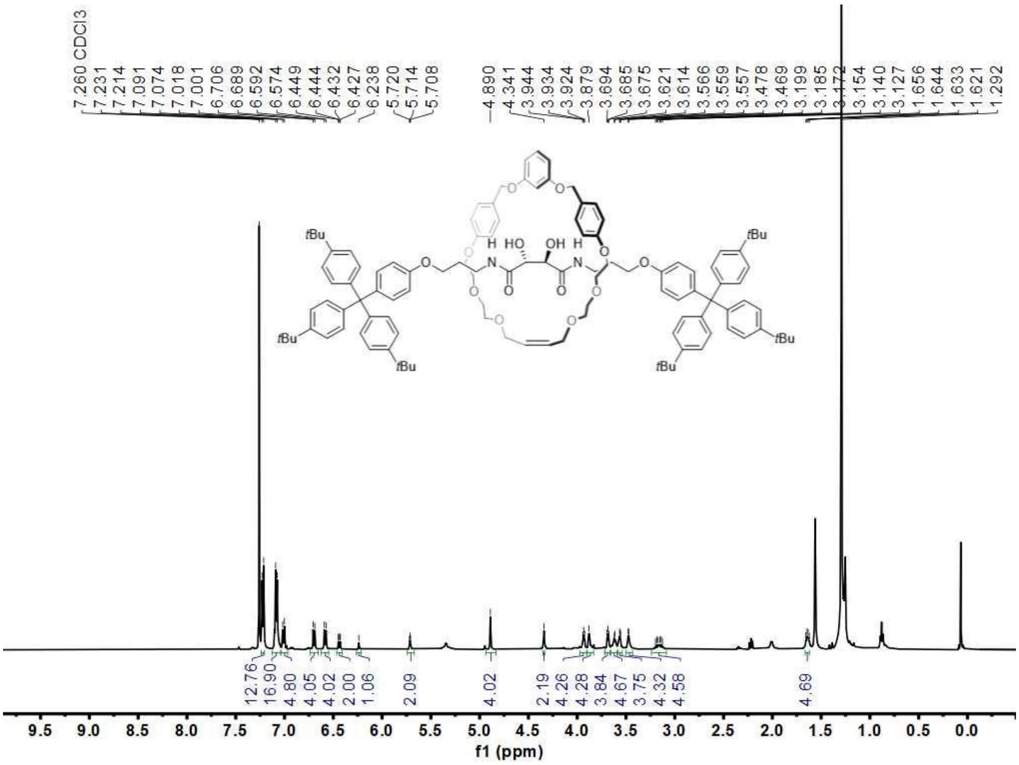

Spectrum S50. ^{1}H NMR spectrum (500 MHz, $CDCl_3$, 298 K) of **8**.

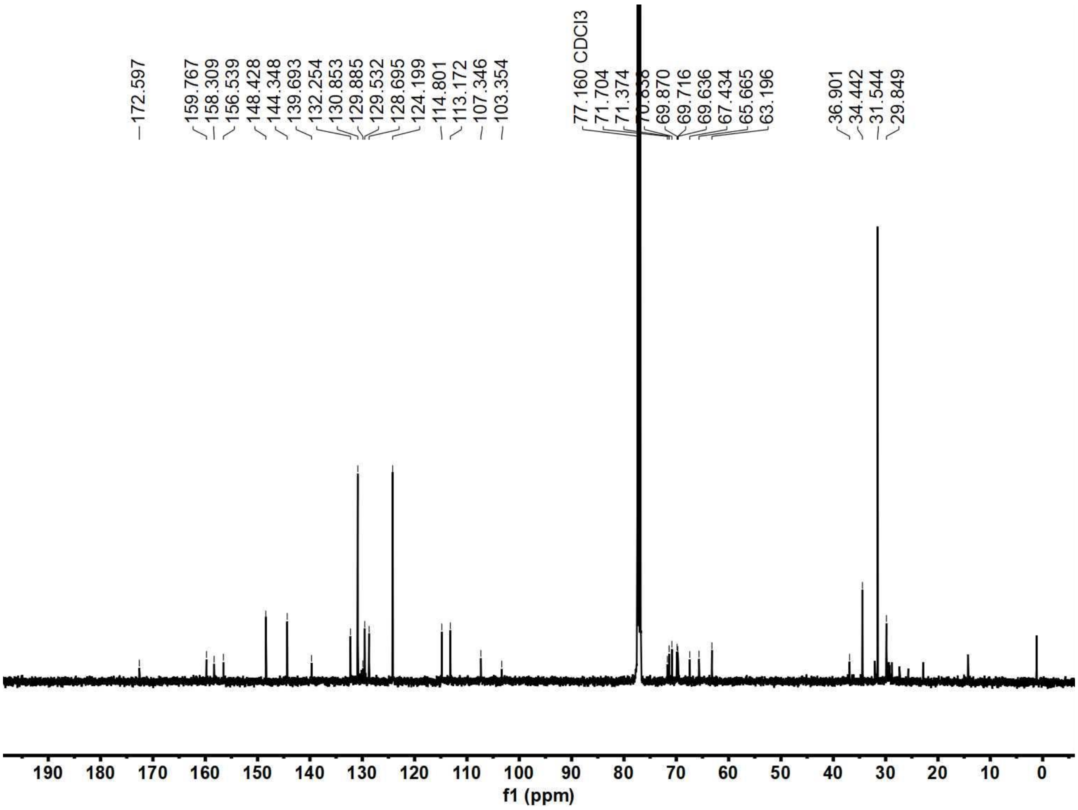

Spectrum S51. ^{13}C NMR spectrum (126 MHz, $CDCl_3$, 298 K) of **8**.

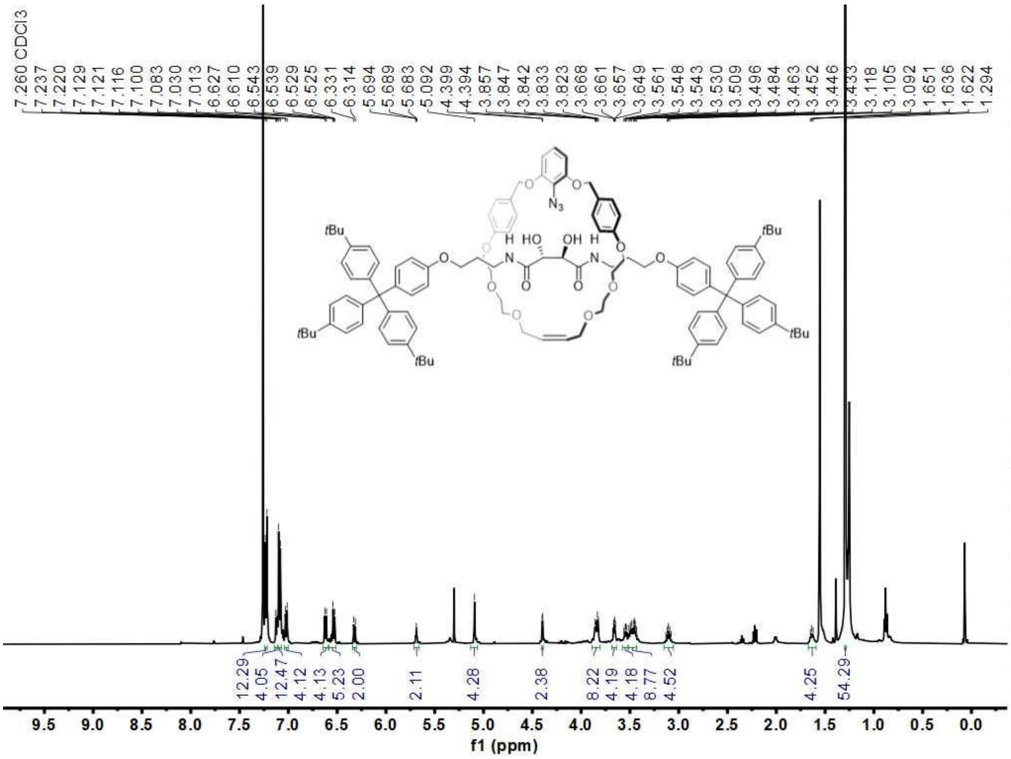

Spectrum S52. ^{1}H NMR spectrum (500 MHz, $CDCl_3$, 298 K) of **9**.

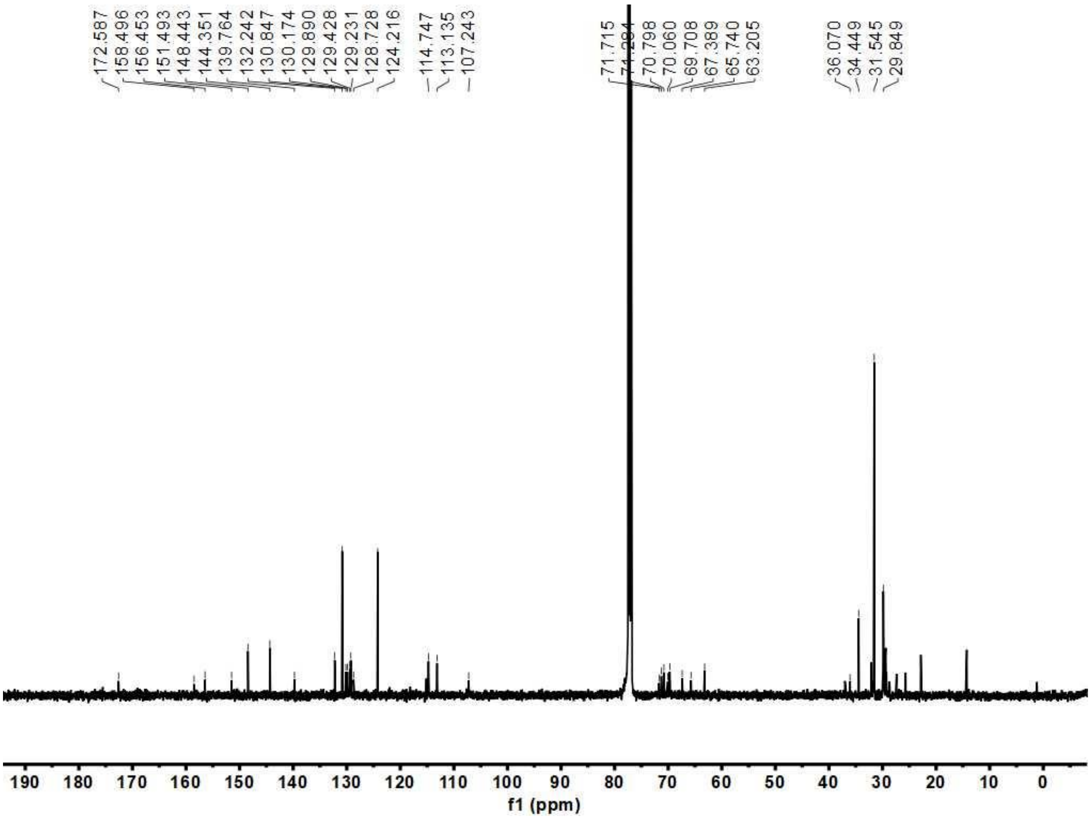

Spectrum S53. ^{13}C NMR spectrum (126 MHz, $CDCl_3$, 298 K) of **9**.

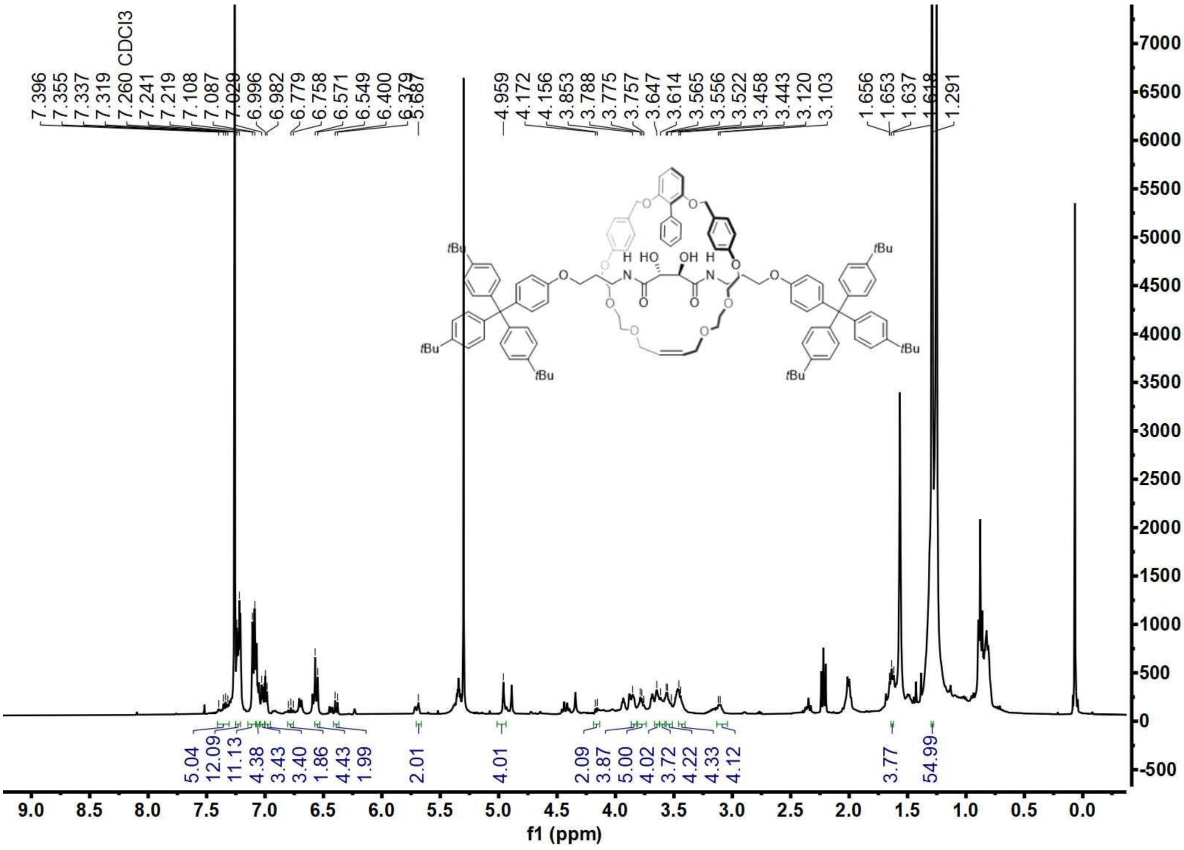

Spectrum S54. ^{1}H NMR spectrum (500 MHz, $CDCl_3$, 298 K) of **10** in mixture with **8** (ratio 55:45 **10**/**8**, integrals only marked for **10**).

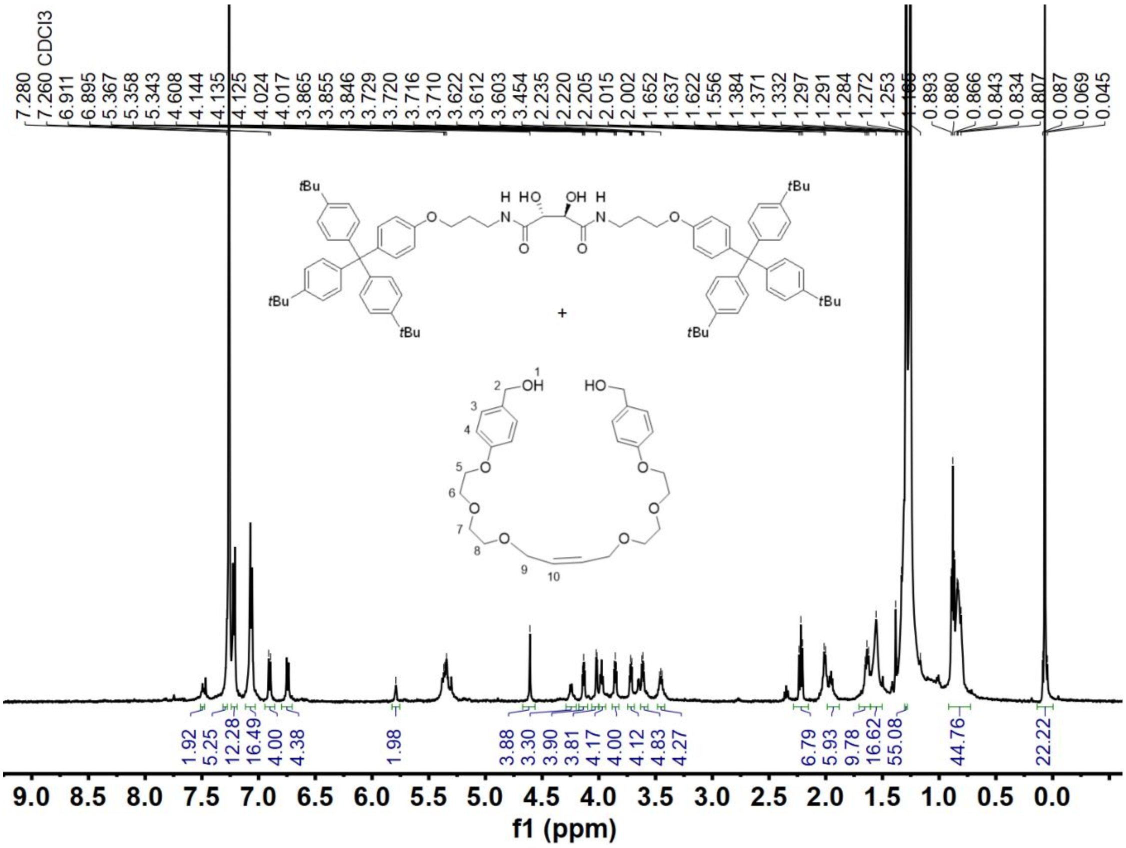

Spectrum S55. ^{1}H NMR spectrum (500 MHz, $CDCl_3$, 298 K) of crude **S11**.

S8. REFERENCES

[1] S. Sarkar, P. Sarkar, P. Ghosh, *Org. Lett.* **2018**, *20*, 6725−6729.

[2] S. Hoekman, M. O. Kitching, D. A. Leigh, M. Papmeyer, D. Roke, *J. Am. Chem. Soc*. **2015**, 137, 7656−7659.

[3] J. Beswick, V. Blanco, G. De Bo, D. A. Leigh, U. Lewandowska, B. Lewandowski, K. Mishiro, *Chem. Sci*. **2015**, 6, 140–143.

[4] S. Shinkaruk, B. Bennetau, P. Babin, J.-M. Schmitter, V. Lamothe, C. Bennetau-Pelissero, M. C. Urdaci, *Bioorg. Med. Chem*. **2008**, 16, 9383–9391.

[5] F. Neese, *WIREs: Comput. Mol. Sci*. **2012**, *2*, 73–78.

[6] F. Neese, *WIREs: Comput. Mol. Sci*. **2022**, *12*, e1606.

[7] R. Sure, S. Grimme, *J. Comput. Chem*. **2013**, *34*, 1672–1685.

[8] M. F. Bush, Z. Hall, K. Giles, J. Hoyes, C. V. Robinson, B. T. Ruotolo, *Anal. Chem*. **2010**, *82*, 9557–9565.

[9] V. Shrivastav, M. Nahin, C. J. Hogan, C. Larriba-Andaluz, *J. Am. Soc. Mass Spectrom*. **2017**, *28*, 1540–1551.
